# Multimodal protective and susceptibility clusters in paediatric atopic dermatitis: A machine learning-based, data-driven observational study

**DOI:** 10.1371/journal.pmed.1004917

**Published:** 2026-09-08

**Authors:** Damir Zhakparov, Nonhlanhla Lunjani, Marco Schmid, Kathleen Moriarty, Damian Roquero, Anita Dreher, Jeannette I. Heldstab-Kast, Kari C. Nadeau, Cezmi Akdis, Michael Levin, Carol Hlela, Milena Sokolowska, Liam O’Mahony, Katja Baerenfaller

**Affiliations:** 1 Swiss Institute of Allergy and Asthma Research (SIAF), University of Zurich, Davos, Switzerland; 2 Swiss Institute of Bioinformatics (SIB), Lausanne, Switzerland; 3 Division of Dermatology, University of Cape Town, Cape Town, South Africa; 4 APC Microbiome Ireland, University College Cork, Cork, Ireland; 5 University of Applied Sciences of the Grisons, Chur, Switzerland; 6 ETHZ, D-BSSE, Basel, Switzerland; 7 Christine Kühne-Center for Allergy Research and Education, Davos, Switzerland; 8 Department of Environmental Health, Harvard T.H. Chan School of Public Health, Boston, Massachusetts, United States of America; 9 Division of Paediatric Allergy, Department of Paediatrics and Child Health, University of Cape Town, Cape Town, South Africa; 10 Department of Medicine, University College Cork, Cork, Ireland; 11 School of Microbiology, University College Cork, Cork, Ireland; The Hospital for Sick Children, CANADA

## Abstract

**Background:**

Atopic dermatitis (AD) is a chronic inflammatory skin disease that typically develops in early childhood. Differences in AD prevalence and allergy sensitisation patterns have been observed in African populations, including the AmaXhosa population in South Africa, suggesting alternative pathogenic and immune mechanisms underlying AD. Differences in AD prevalence have also been documented between urban and rural communities, making AmaXhosa children, who share a common ethnogenetic background but differ in environmental exposures, a unique population in which to investigate environmental and immune mechanisms underlying AD. To address this, we performed a machine learning (ML)-based multimodal observational study to identify features associated with AD in 217 AmaXhosa children.

**Methods and findings:**

To gain deeper insights into AD pathogenesis, we re-analysed a previously established multimodal dataset comprising environmental, cytokine, antibody, and transcriptomic data from healthy AmaXhosa children and children with AD, aged 12–36 months, living in rural or urban settings. We applied ML to analyse each data modality individually and subsequently integrate them to identify multimodal signatures associated with AD. Specifically, we used the GeneSelectR workflow to identify informative genes, SHAP values to explain the ML outputs, and DIABLO to integrate the datasets and identify protective and susceptibility clusters.

In the environmental and antibody datasets, we found that the combined effects of environmental features and higher levels of allergen-specific and total IgE antibodies contributed to the prediction of AD. In the transcriptomic dataset, we identified a subset of 560 genes that discriminated between children with and without AD and used these for the subsequent analyses. In the integrated analysis, we identified three multimodal clusters associated with AD status. One cluster associated with the healthy phenotype comprised environmental features primarily found in the rural setting, which correlated with plasma cytokine levels and the expression of autophagy-related genes. Two additional clusters were characterised by correlations between allergen-specific and total IgE antibodies and the cytokines MCP-4 and TARC, and by a transcriptomic feature signature associated with the AD endotype.

Limitations of this study include the exploratory nature of the explainable ML framework and the lack of validation in an independent cohort.

**Conclusions:**

Complementary ML approaches enabled explainable analysis of a complex multimodal dataset. Integrated analyses identified a multimodal protective cluster comprising environmental, cytokine, and transcriptomic features, together with two AD susceptibility clusters, one dominated by transcriptomic features and the other by correlated cytokine and antibody levels. These findings improve our understanding of the factors associated with AD. Furthermore, the feature-selection and integration workflow provides a framework for analysing complex multimodal datasets in future biomedical research.

## Author summary

**Why was this study done?:**

• Epidemiological studies have shown that atopic dermatitis (AD) is generally less common in developing countries than in developed countries, and that filaggrin (FLG) gene mutations, the strongest known genetic risk factor for AD, are absent from African populations, including the AmaXhosa population with AD.
• The AmaXhosa community additionally shows distinct AD prevalence and allergy sensitisation patterns, pointing to robust alternative pathogenic and immune mechanisms underlying AD.
• Differences in AD prevalence have also been documented between urban and rural communities, suggesting that environmental exposures interact with immune mechanisms to influence disease risk.
• AmaXhosa children living in rural and urban environments share a common ethnogenetic background but experience different environmental conditions, providing a unique setting to investigate mechanisms associated with protection from or susceptibility to AD.

**What did the researchers do and find?:**

• We performed a data-driven, multimodal observational study by integrating previously collected questionnaire data on living and health conditions, plasma antibody and cytokine measurements, and RNA sequencing data from healthy AmaXhosa children and children with AD living in rural and urban South Africa.
• We applied explainable machine learning (ML) to identify the features most strongly associated with AD and found that allergen-specific and total IgE levels, together with environmental factors linked to rural or urban living, were among the strongest predictors distinguishing children with and without AD.
• Using a newly developed workflow, we identified a reduced set of biologically relevant transcripts with high predictive capacity, enabling the integration of RNA sequencing data with the other data modalities.
• Integrating all data types revealed multimodal clusters of correlated environmental, immune, and transcriptomic features associated with either healthy children or children with AD.

**What do these findings mean?:**

• Integrating environmental, immune, and gene expression data enabled the identification of three multimodal susceptibility and protective clusters associated with AD, capturing both established and newly identified AD-associated features. The protective cluster comprised environmental features primarily found in the rural setting that correlated with plasma cytokine levels and the expression of autophagy-related genes. In the first AD susceptibility cluster, allergen-specific and total IgE antibody levels correlated with the cytokines MCP-4 and TARC, while the second was characterised by an AD-specific transcriptomic signature.
• The findings show that explainable ML can help uncover meaningful relationships within complex biomedical datasets while providing transparent insights into how different features contribute to disease.
• Our approach demonstrates that selecting the most informative gene expression features before integration improves the analysis of multimodal datasets and provides a framework for integrating complex datasets that may support future research on AD and other diseases in which environmental, molecular, and clinical factors interact.
• Limitations of this study include that the ML analyses were designed to explain biological mechanisms in this observational study rather than to develop a predictive model for clinical use, and that validation in an independent cohort is needed to confirm the generalisability and robustness of the identified AD-associated features.

## Abstract 3

In this study, Damir Zhakparov and colleagues use machine learning to identify multimodal feature clusters associated with paediatric atopic dermatitis in AmaXhosa children in South Africa, highlighting a rural-environment-linked cluster associated with healthy skin and two susceptibility-related clusters.

## 1. Introduction

Atopictitisa derm (AD) is a complex chronic skin disease characterised by profound itching and eczematous lesions. It is caused by a complex interplay of several factors, including genetic predisposition, microbial dysbiosis, barrier dysfunction, immune dysregulation, and environmental exposures [1]. Typically, AD starts in early childhood, and children with AD often develop food allergies, asthma, or allergic rhinitis, a progression known as the atopic march [2,3]. AD affects around 22.6% of children worldwide [4]. In urban African centres, the prevalence of AD is rapidly increasing, approximating rates observed in North America and Europe, and it is one of the most common skin disorders in this region [5–7]. In South Africa, allergic diseases were shown to be less common in rural areas compared to urban settings [8]. Due to differences in environmental and genetic backgrounds, the pathogenesis and clinical presentation of AD vary considerably. For example, whilst AD patients from Tanzania have comparable disease severity as Swiss AD patients, they have different IgE sensitisation patterns and serological immune signatures [9]. Estimating disease prevalence can also be challenging in different countries. For example, the U.K. criteria for atopic eczema were found to perform poorly in AmaXhosa communities in South Africa, likely due to translation and cultural issues, and therefore cannot be recommended as a primary outcome measure. Instead, the single parameter of visible flexural eczema performed well for diagnosing AD [10]. Also, the number of multi-omic AD studies performed in Africa is lower than in other regions, leading to an underrepresentation bias in understanding the complexity of AD pathogenesis. Thus, we sought to identify factors associated with AD in early childhood in the South African population using systems biology approaches.

To do this, we used a variety of machine learning (ML) methods to analyse and integrate data previously published by our groups, including questionnaire data on health and living conditions, cytokine and antibody data, and RNA sequencing (RNA-Seq) data of peripheral blood mononuclear cells (PBMCs) from AmaXhosa children in South Africa with and without AD living in urban and rural areas with the aim to identify distinct features associated with AD. The analysis of the individual datasets, and particularly their integration, posed several challenges, as the data are complex, multidimensional, and influenced by a strong environmental factor, with substantial differences in the number of features across the datasets. To address this, we first used ML to identify the most important features in the clinical questionnaire and antibody data. This was followed by the calculation of SHapley Additive exPlanation (SHAP) values to understand the contributions of these features to the results [11]. For the RNA-Seq data, we applied the dedicated GeneSelectR workflow [12] to select a subset of genes with high discriminatory power and biological relevance. Finally, we integrated all four datasets using Data Integration Analysis for Biomarker discovery using Latent cOmponents (DIABLO) [13] to identify multimodal clusters of correlated environmental, cytokine, and RNA-Seq features. Using this workflow, we aimed to identify the environmental, immune, and transcriptomic features most strongly associated with AD and to determine whether multimodal data integration could reveal biologically meaningful susceptibility and protective clusters. AmaXhosa children, who share a common ethnogenetic background but differ in environmental exposures, provide a unique population in which to investigate the environmental and immune mechanisms underlying paediatric AD.

## 2. Materials and methods

### Ethics statement

The study received the University of Cape Town Faculty of Health Sciences Human Research Ethics Committee approval (HREC 451/2014) and was conducted in accordance with the Declaration of Helsinki [14]. Written informed consent was obtained from parents or guardians of all participants prior to inclusion in the study. Additional information regarding the ethical, cultural, and scientific considerations specific to inclusivity in global research is included in the Supporting Information (S1 Checklist: Inclusivity in Global Research).

### Study description and questionnaires

As detailed in [8,15] this study employed a single-visit, cross-sectional case-control design involving AmaXhosa children in South Africa who were between 12–36 months old, lived in either a rural or urban environment, and were either healthy or had a dermatologist’s diagnosis of moderate to severe AD, fulfilling the UK Working Party criteria for AD. Exclusion criteria included other considerable health conditions, immune-mediated diseases other than allergic comorbidities and positive stool and serum IgE tests for helminths. Urban children with moderate to severe AD were enrolled at the Paediatric Dermatology Clinic, Red Cross Children’s Hospital, Cape Town. Healthy nonallergic, nonfood sensitised (on skin prick test (SPT) screening) participants were enrolled from randomly selected crèches in the Cape Town metropole. Similarly, children from a rural setting with AD were enrolled at the dermatology clinic at the Nelson Mandela Academic Hospital, Umthatha. Their nonallergic healthy control counterparts were randomly selected at study sites based at village clinics in the Mqanduli district of Umthatha. All participants were seen at one time point for questionnaire, SPT, clinical assessment, and blood sample collection. In total, standard questionnaire data on environmental exposures and living conditions of the children were obtained for 217 AmaXhosa children (rural_AD: 60, rural_healthy control (HC): 52, urban_AD: 56, urban_HC: 49). For 152 children, we additionally had antibody data, for 149 children we had bulk RNA-Seq data, and for 159 children we had plasma cytokine data (Table A, Fig A in S1 Appendix).

### Allergen sensitisation, allergen antibody and cytokine measurements

To establish the antibody data that form part of the clinical dataset, SPTs were performed as previously reported in [8], using Alk-Abello (Alk-Abello, Denmark) reagents for sensitisation to cow’s milk, egg white, peanut, hazelnut, wheat, soy, fish, raw egg white, fresh cow’s milk, and fresh peanut. ImmunoCap assays (Thermo Fisher Scientific, Sweden) were used for quantifying the antibody levels of total IgE, specific IgE against *D. pteronyssinus*, milk, fish, wheat, peanut, soy, hazelnut, egg, and CCD, and specific IgG4 against *D. pteronyssinus*, fish, wheat, peanut, soy, hazelnut, casein, and egg.

The levels of plasma analytes and cytokines were assessed using the Meso Scale Discovery multi-spot assay system and QuickPlex SQ120 platform (Meso Scale Discovery, USA). The cytokine data were subjected to statistical analysis testing for differences between HC and AD groups using a Mann–Whitney test, followed by correction for multiple testing with Bonferroni (Table B in S1 Appendix).

### ML on questionnaire and antibody data

As reported previously, structured questionnaires were used to collect data on family history of atopy and allergy, medical history, household demographics, early-life exposures and various environmental factors [8] (S1 File). The questionnaire features were pre-processed to exclude features mainly consisting of missing values or limited information, as well as those that were highly imbalanced between the two groups. Features directly associated with AD, such as the SCoring Atopic Dermatitis (SCORAD) score or medication with antihistamines, were marked for exclusion from the analyses, while features with more than two possible entries, such as the kind of fuel used for heating or cooking, were one-hot encoded as separate features. The combined pre-processed questionnaire and antibody data were subjected to statistical analysis to identify differences between HC and AD groups. A hypergeometric test was used for categorical variables, and a Mann–Whitney test was used for numerical features. The results were corrected for multiple testing using the Bonferroni method (Table C in S1 Appendix).

In the ML classification, AD versus HC was set as the target variable. All data were included to build the models using cross-validation (CV) as the main aim was not to build a single best model, but to extract the features that contributed most to predictive performance. To conduct the hyperparameter search for the models, the Bayesian optimisation-based Optuna framework was used, which employs the Tree-structured Parzen Estimator (TPE) to navigate the hyperparameter search space in an informed manner. Unlike classical random or grid searches, Optuna samples combinations of hyperparameters, including algorithm choice, that yield better performance, while deprioritising less optimal ones. This approach enabled the selection of hyperparameter settings that were most likely to yield a high classification score [16]. As the stopping criterion, limits of n_trials = 150 and a timeout of 36,000 s were defined, and the timeout was reached first after 78 trials were executed. The hyperparameter search space also included selection of the ML algorithms RandomForestClassifier [17,18], Multi Level Perceptron (MLP) [18,19], Gaussian Naive Bayes (GNB) [18,20] or eXtreme Gradient Boosting (XGBOOST) [21]. In running the ML pipeline, missing data were initially imputed using the miceforest package [22], which employs RandomForest to estimate the missing values. This approach was selected because of the uneven distribution of missing values between AD and HC, which could otherwise influence the classification. The features were subsequently scaled, and quasi-constant features were removed using a variance threshold of 0.15. To avoid overfitting, the feature selection procedure was constrained to select a minimum of 5 and a maximum of 30 features. The pipeline was then evaluated for every hyperparameter combination in a 5-fold CV process, using mean accuracy as the classification performance measure, as the distribution between AD and HC in the data is nearly equal. As described in detail in S2 File, all data preprocessing steps were applied separately within each CV fold. After the thorough model building process, 78 models were trained and the best model performance was reached in Model 52 (M52). This best-performing Random Forest model had a CV mean accuracy score of 0.81. Other classification metrics were similarly high (approximately 0.80) for both CV and the test set (Fig B in S1 Appendix). After obtaining the CV accuracy scores, all models were refitted on the full dataset with their respective parameter settings to generate the final SHAP [11] values to interpret the outputs of the ML models. Inspecting the importance of the different hyperparameter search space features revealed that the choice of model had the greatest impact on the objective function, while scaling and imputation contributed little to the classification (Fig C in S1 Appendix).

### Feature selection in RNA-Seq data

As described previously in [15], total mRNA was isolated from PBMCs and sequenced using HiSeq 40,000. The FASTQ sequencing data were preprocessed using the ARMOR pipeline with integrated differential gene expression (DGE) analysis using edgeR [23]. From the original 150 RNA-Seq samples one outlier was identified in the Principal Component Analysis and excluded from analyses when rerunning the pipeline. The final RNA-Seq data were from PBMCs of 149 AmaXhosa children living in urban or rural environments and being healthy (HC) or suffering from AD (groups: urban_HC = 29; urban_AD = 31; rural_HC = 44; rural_AD = 45). These four groups were set as comparisons in the design matrix of edgeR. Transcripts were considered significantly changed between two conditions if they had a false discovery rate (FDR) < 0.05 and a fold change (FC) > 1.5.

To select relevant genes for subsequent data integration, we utilised the GeneSelectR R workflow that we recently developed [12], designed to overcome shortcomings associated with classical DGE analysis in complex datasets. Initially, the input transcript count data were normalised using a between-sample normalisation approach from the edgeR R package [23]. Subsequently, the workflow was used to select informative genes (features) based on ML metrics and their biological relevance across several steps:

(1) A standard ML pipeline for feature selection incorporating four ML methods (Logistic Regression with L1 Penalty (Lasso), Random Forest, Boruta and Univariate filtering (Univariate)), with hyperparameter adjustment was applied.
(2) Features were ranked based on their inbuilt method-specific feature importance scores. Additionally, feature ranking across different iterations was reported.
(3) Genes meeting the filtering criterion to appear in at least 2 out of 10 iterations were assembled into a gene list for every method.
(4) A GO enrichment analysis was conducted on each gene list. GO categories with an adjusted *p*-value < 0.05 were considered enriched and subsequently subjected to semantic similarity analysis [24].

The comprehensive breakdown of the analysis is available in S3 File.

### Weighted Gene Co-expression Network (WGCNA)

The weighted gene co-expression network analysis (WGCNA) was performed using the official R package, as described in [25]. In summary, a correlation matrix between genes in the dataset was calculated to detect co-expression patterns among the genes, and genes with similar patterns were clustered into modules. These gene modules were then correlated with the ML-selected questionnaire features using the cor() function.

### ML on integrated data

To combine different data modalities, we adopted a late data integration strategy [26,27]. In this approach, feature selection is performed on each sub-dataset separately to reduce dimensionality and avoid introducing redundant noise into the combined dataset. The sub-datasets were then matched using a complete-case approach, resulting in 132 samples with patient information, antibody data, RNA-Seq data, and quantitative cytokine data (Fig A in S1 Appendix).

To integrate the RF560 RNA-Seq, the combined antibody and questionnaire, and the cytokine datasets, the DIABLO method from the mixOmics package was used [13]. In brief, DIABLO is a supervised ML method aimed at decomposing the dataset into components projected into latent space and correlated with the label of interest. Using DIABLO, we observed that the datasets had high pairwise correlation scores, suggesting that there is a common pattern across the data modalities (Fig D in S1 Appendix). This is also reflected in the latent space projection sample plot, where samples are distinctly grouped based on the labels AD or HC (Fig E in S1 Appendix).

## 3. Results

### ML applied to questionnaire and antibody data reveals key variables associated with promotion of AD

The study cohort consisted of 217 AmaXhosa children for whom questionnaire data on environmental exposures and living conditions were available, divided into four groups according to whether they lived in rural or urban areas and whether they were healthy or had a dermatologist-confirmed diagnosis of moderate-to-severe AD (Table A in S1 Appendix). Statistical analysis of the pre-processed questionnaire data combined with the antibody data revealed that, among the environmental variables, the use of electricity or gas as fuel for cooking differed significantly between HC and AD (Table C in S1 Appendix). To go beyond these univariate tests, we employed various ML algorithms with HC versus AD as the target variable in a classification process to identify distinguishing features between these groups. By calculating Jaccard scores to assess the similarities between the feature lists selected by the top three models of each ML algorithm, we found that Random Forest achieved the highest feature stability (Fig F in S1 Appendix). Inspecting the features that were selected in these three best-performing Random Forest models revealed an overlap of 18 features. These features include total IgE antibody levels, as well as the levels of specific IgE and IgG4 antibodies against house dust mites (sIgE_Der and sIgG4_Der) and specific IgE antibodies against different food. Additional features include exposure to food such as eggs, the type of fuel used for heating or cooking, and the duration of sunlight exposure (Table D in S1 Appendix).

**Fig 1:**
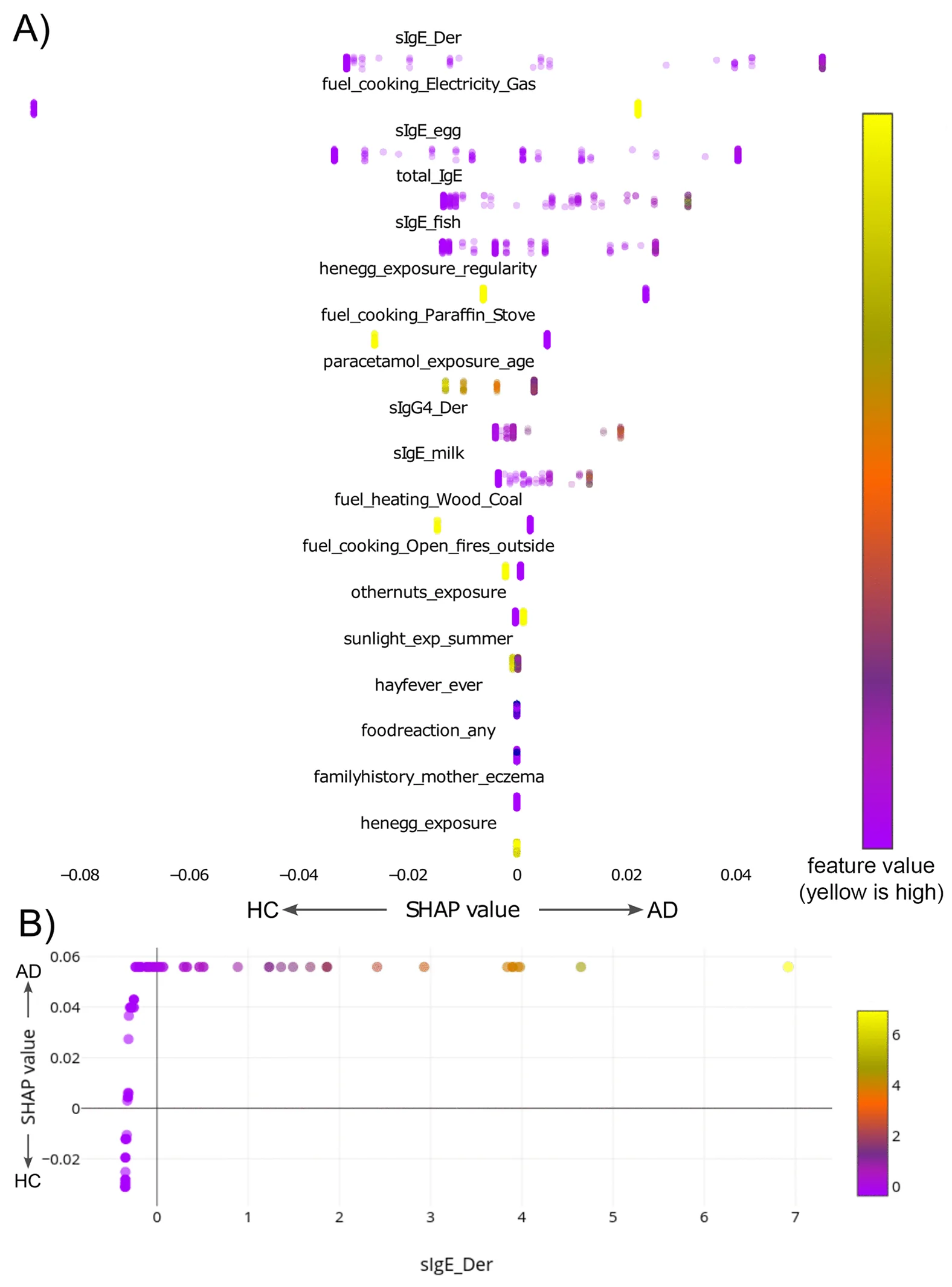
SHAP values of the importance and the impact of the individual features on the output of the best-performing model applied to the combined antibody and questionnaire data. **A)** The SHAP values of the 18 Random Forest-selected features with the highest impact in target prediction indicate the degree of change towards predicting AD in log odds. Each dot represents a row of data from the questionnaire or antibody data, with the colour of each point indicating the value of the corresponding feature through a colour gradient from yellow (high value), transitioning through red, to purple (low value). **B)** SHAP dependence plot for the levels of specific IgE antibodies against house dust mites.

To better explain the model output, SHAP values were calculated, which measure how much each of these features contributes to the prediction of the best-performing model. The SHAP values show that the levels of sIgE antibodies against house dust mites (sIgE_Der) and against egg (sIgE_egg), as well as using electricity or gas as fuel for cooking are the three most important features with the greatest influence (Fig G in S1 Appendix). While feature importance quantifies the contribution of each feature to the model prediction, it does not specify whether high or low feature values correlate with the prediction of AD. To address this, the influence of individual features on predicting AD is illustrated in Fig 1A. The findings reveal that higher levels of specific IgE against house dust mites, egg, fish, milk, and total IgE are associated with the prediction of AD, as shown in the SHAP dependence plots (Figs 1B, H–K in S1 Appendix). Similarly, higher levels of specific IgG4 antibodies against house dust mites were positively associated with the prediction of AD (Fig L in S1 Appendix), as well as no regular exposure to hen egg (Fig 1A). Of the living environment features, using electricity or gas for cooking positively influences the prediction of AD, whereas not using them contributes towards predicting HC. Conversely, using a paraffin stove or fires outside the house for cooking, or wood or coal for heating contributes towards predicting HC (Fig 1A). Finally, a higher age of first exposure to paracetamol is associated with the prediction of HC. These analyses demonstrate that, although individual features in the questionnaire data may not show significant differences in univariate statistical tests, their combined effect contributes to predicting AD.

### Adopting a novel ML-based RNA-Seq analysis pipeline revealed a transcript signature of AD

The dataset-wide normalised RNA-Seq data of 149 AmaXhosa children were first analysed with DGE analysis testing for statistical differences between HC and AD, and between rural and urban. As expected from the results of the principal component analysis (Fig M in S1 Appendix), significantly more transcripts changed between locations than between diagnoses (Table E in S1 Appendix), indicating a strong influence of the environment on the data distribution. For the differences between AD and HC, we found only one gene, Proline Rich Coiled-Coil 2A (PRRC2A), which was present in both the 36 DEGs for the rural samples and the 82 DEGs for the urban samples (Table E in S1 Appendix). In addition, we found no significant correlation in the FCs between AD and HC for these genes in urban and rural samples. (Fig N in S1 Appendix). Combined, these findings indicate the limited capability of DGE analysis to identify differential features within a complex dataset where two variables contribute differently to the data distribution.

Considering the univariate nature and limitations of DGE, we used the GeneSelectR workflow [12] to identify a subset of genes distinguishing between AD and HC using ML, as this approach also considers complex nonlinear relationships between features. We employed the four ML-based feature selection methods implemented in GeneSelectR, which are Random Forest (RF), Lasso, Boruta and Univariate Filtering (Univariate). Feature selection based on the inbuilt feature importance resulted in lists with varying numbers of transcripts (RF: 560, Lasso: 372, Univariate: 198, Boruta: 53). The classification performance according to different metrics was similar, with a CV mean score of around 0.7. However, RF scored slightly higher than the other ML methods (Fig O in S1 Appendix). Calculating three different overlap coefficient metrics implemented in GeneSelectR revealed that there was only minimal overlap and no consensus gene signature shared across the lists (Fig P in S1 Appendix).

**Fig 2:**
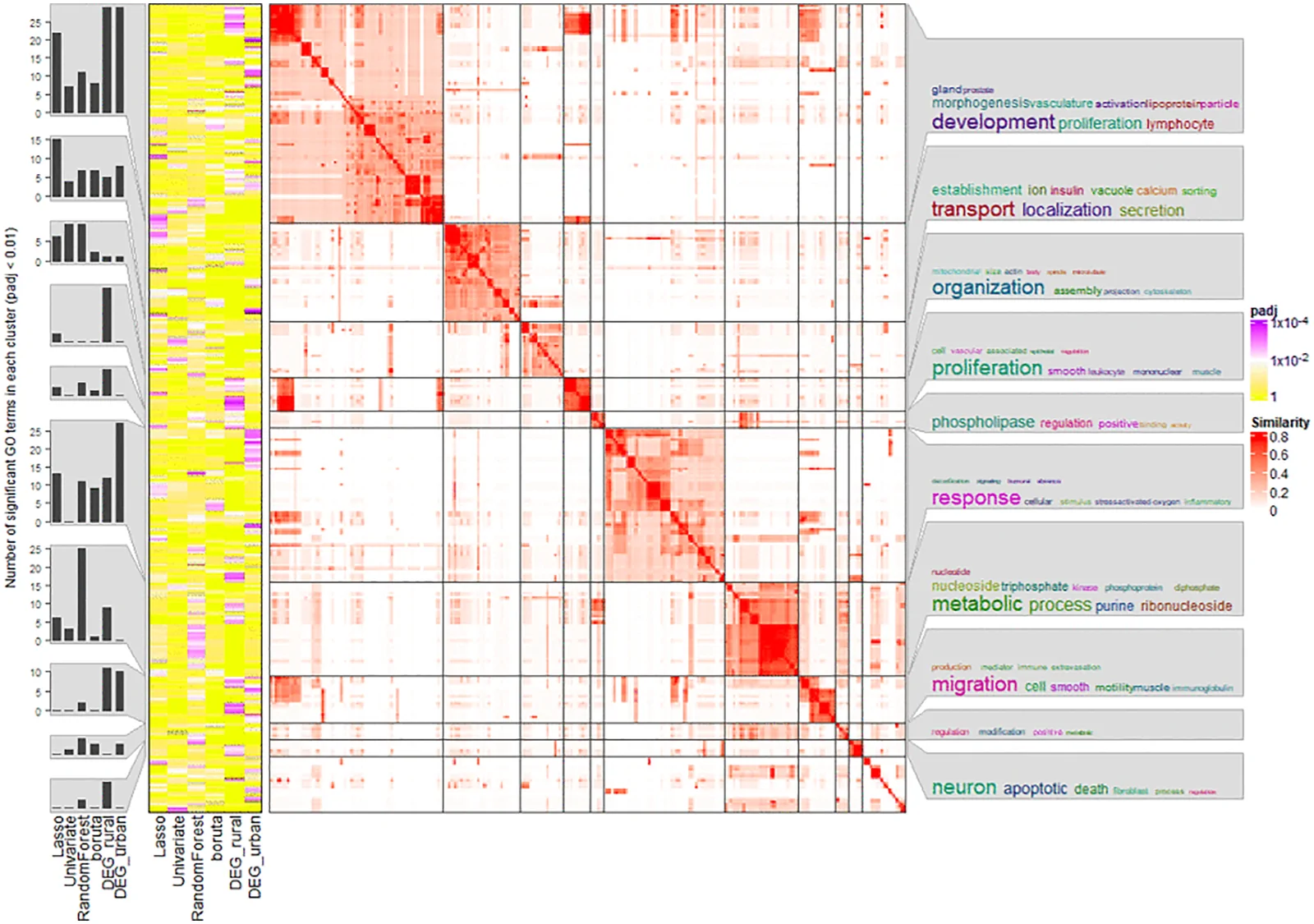
Gene lists associated with AD identified by semantic similarity clustering of enriched GOBP terms. Heatmap of the semantic similarity clustering of the 379 GOBP terms passing an unadjusted *p*-value threshold of 0.01 in a functional overrepresentation analysis of the feature lists selected by different ML methods and in the DEGs. The composition of each of the 10 clusters is described by the word clouds on the right. At the left, the smaller heatmap shows the significance of the enrichment of the different GOBP terms, with a gradient from yellow to purple, where purple indicates a low adjusted *p*-value (padj), and the histograms demonstrate the number of enriched GOBP terms in each of the clusters.

As neither the classification performance metrics nor the overlap coefficients allowed for determining which feature list should be used in downstream analyses, the next step was to evaluate their biological relevance through an overrepresentation analysis of Gene Ontology Biological Process (GOBP) terms. In all the lists, a total of 379 enriched GOBP terms were identified. These functional terms were clustered with binary cut based on their semantic similarity using the topology information from GO. Of the 10 identified clusters, three stood out as more specific to the diagnosis of AD because they were related to immune processes: cluster 1 (lymphocyte development and proliferation), cluster 6 (inflammatory response and oxidative stress), and cluster 7 (metabolism and immune cell processes) (Fig 2A). The 560 features selected with the RF method, termed RF560 list, prominently featured terms from clusters 6 and 7, establishing its significance in AD biomarker identification. In conclusion, the RF-derived list emerged as the most suitable for delineating AD characteristics in the cohort for the subsequent analyses.

### Weighted co-expression gene network analysis demonstrates a high correlation of the RF560 list with significant questionnaire and antibody features

**Fig 3:**
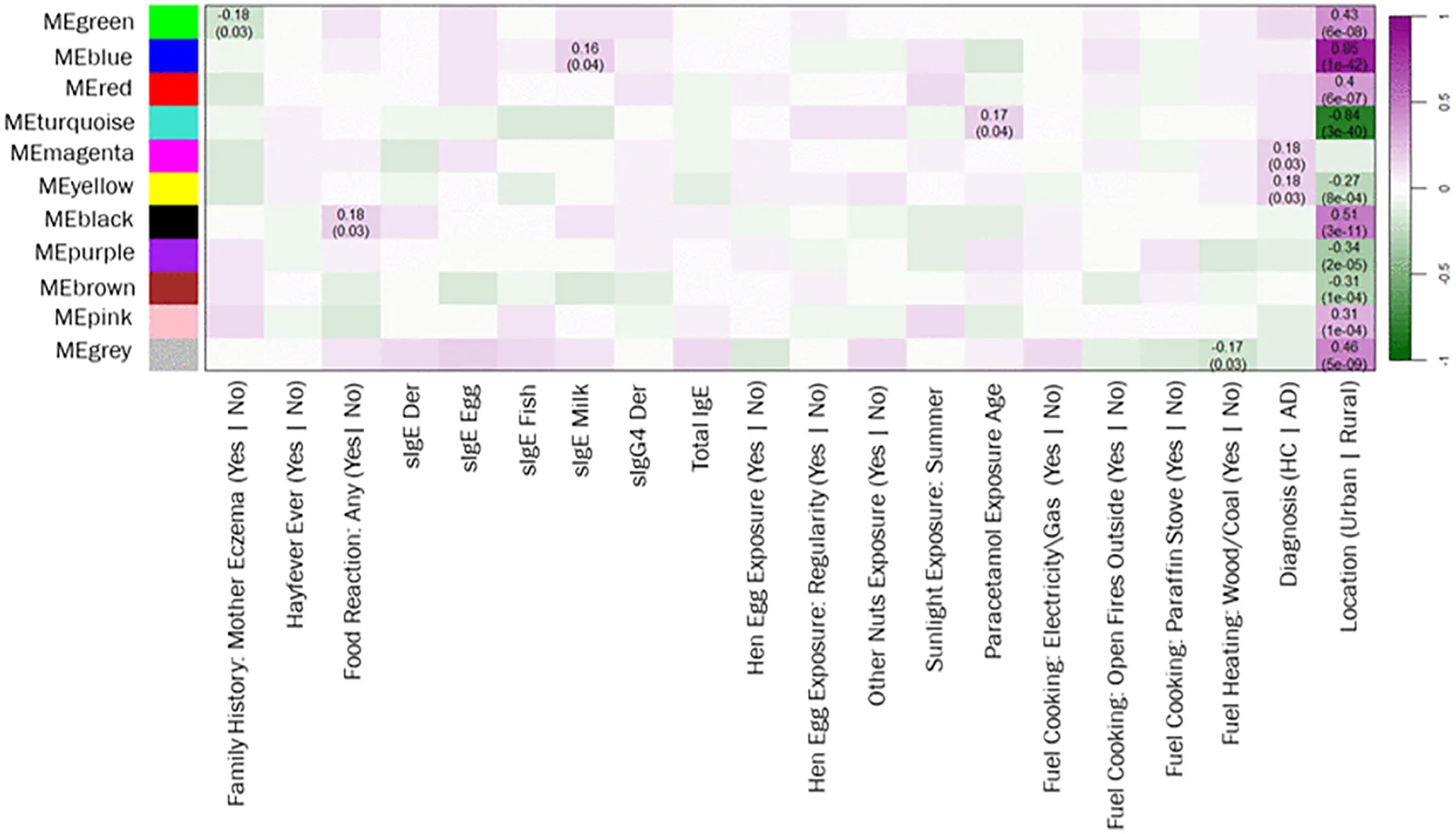
Correlations of co-expressed ML-selected gene clusters with significant questionnaire and antibody features. Heatmap of the correlation between the 11 co-expressed gene clusters identified using WGCNA applied to the RF560 gene list (y-axis) and the 18 questionnaire and antibody features present in the three best RF models plus the major experimental variables location and diagnosis (x-axis). The direction of the correlation is indicated in brackets. For example, Diagnosis (HC | AD) means that the positive correlation of a gene cluster with HC is > 0, while the correlation with AD is < 0. The value of the correlation is indicated with a colour gradient from green to purple, where green indicates a negative correlation and purple indicates a positive correlation. For correlations that passed a p-value threshold of 0.05, the correlation score is indicated along with the p-value in brackets.

The weighted co-expression network analysis (WGCNA) of the genes in the RF560 list revealed 11 clusters of co-expressed genes (S1 Table). These co-expressed gene clusters were correlated with the 18 previously RF-selected questionnaire and antibody features, as well as the two main experimental variables, location (urban versus rural) and diagnosis (HC versus AD), to identify significantly associated features. This association can indicate that the gene clusters play a role in influencing these features. Most gene clusters were significantly correlated with location, underscoring the strong influence of this variable on gene expression. In contrast, only the two clusters (the magenta and yellow clusters shown in Fig 3), which were associated with rural location, also showed a significant positive correlation with healthy status. Furthermore, one cluster (the blue cluster) was significantly positively correlated with levels of specific IgE antibodies against milk, one cluster (the black cluster) with any reaction to food, and one cluster (the grey cluster) was significantly negatively correlated with the use of wood or coal as fuel for heating. In summary, the correlation of specific questionnaire and antibody features with specific gene clusters again emphasises the complexity of the dataset.

### DIABLO integration of all datasets identifies key features related with AD

**Fig 4:**
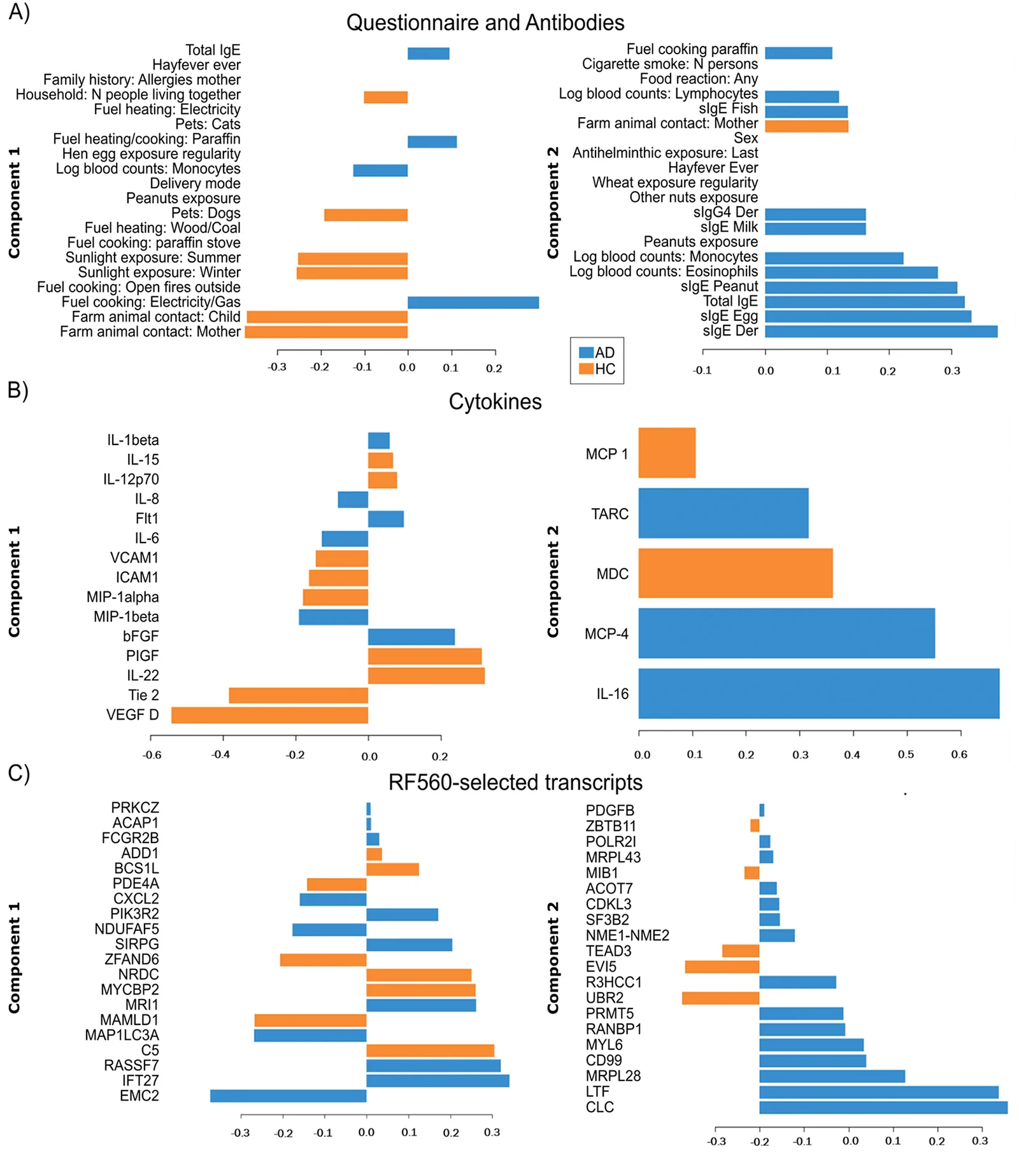
Contributions of variables from the A) combined questionnaire and antibody data, B) the cytokine data, and C) the RF560 selected transcripts to the prediction of AD (blue) or HC (orange) on the first two components of the DIABLO model. The contribution scores of the components are on the *x* axis, and the feature names are on the y- axis. The higher the absolute value of the contribution score, the more important a feature is. Features without a component score in components 1 or 2 have a tie between the two groups, indicating equal contribution in AD and HC.

To further explore features of the four datasets significantly associated with AD, we applied the DIABLO data integration framework [13] to investigate relationships among the combined questionnaire and antibody data, the cytokine data, and the RF560-selected transcripts. To determine which samples drive the separation of HC and AD in the latent space projection plot (Fig E in S1 Appendix), the contributions of variables from the different datasets were visualised on the first two components of the DIABLO model. Since the positive or negative sign of the contribution scores is relevant only in the context of the projection plot, the absolute values of the component contribution scores reflect the importance of each feature (Fig 4).

In the questionnaire and antibody data, the use of electricity, gas, or paraffin as fuel for cooking or heating, higher counts of monocytes, eosinophils, and lymphocytes, as well as elevated levels of specific and total IgE antibodies contributed to the prediction of AD in the first two components (Fig 4A). The questionnaire features contributing most to the prediction of HC were contact with farm animals of the mother or the child, higher exposure to sunlight in summer and winter, and keeping a dog (Fig 4A). These features are all significantly more prevalent in rural living conditions [8].

In the cytokine data, the major contributors to predicting AD in the first two components were IL-1β, IL-8, IL-6, Flt1, MIP-1β, bFGF, TARC, MCP-4, and IL-16 (Fig 1B). The DIABLO model therefore enabled the identification of a greater number of cytokines associated with the diagnosis of AD compared to the univariate statistical test, which identified only Eotaxin-3 and TARC as significantly increased in AD patients compared to HC individuals (Table B in S1 Appendix). Furthermore, DIABLO allowed the identification of transcripts in the RF560 list that contributed most to the prediction of AD or HC. In both components 1 and 2, the major contributors were transcripts predicting AD, including EMC2, IFT27, RASSF7, MAP1LC3A, MRI1, CLC, LTF, MRPL28, CD99, MYL6, RANBP1, and PRMT5. By contrast, the transcripts contributing most to the prediction of HC included C5, MAMLD1, MYCBP2, NRDC, ZFAND6, UBR2, EVI5, and TEAD3 (Fig 4C).

**Fig 5:**
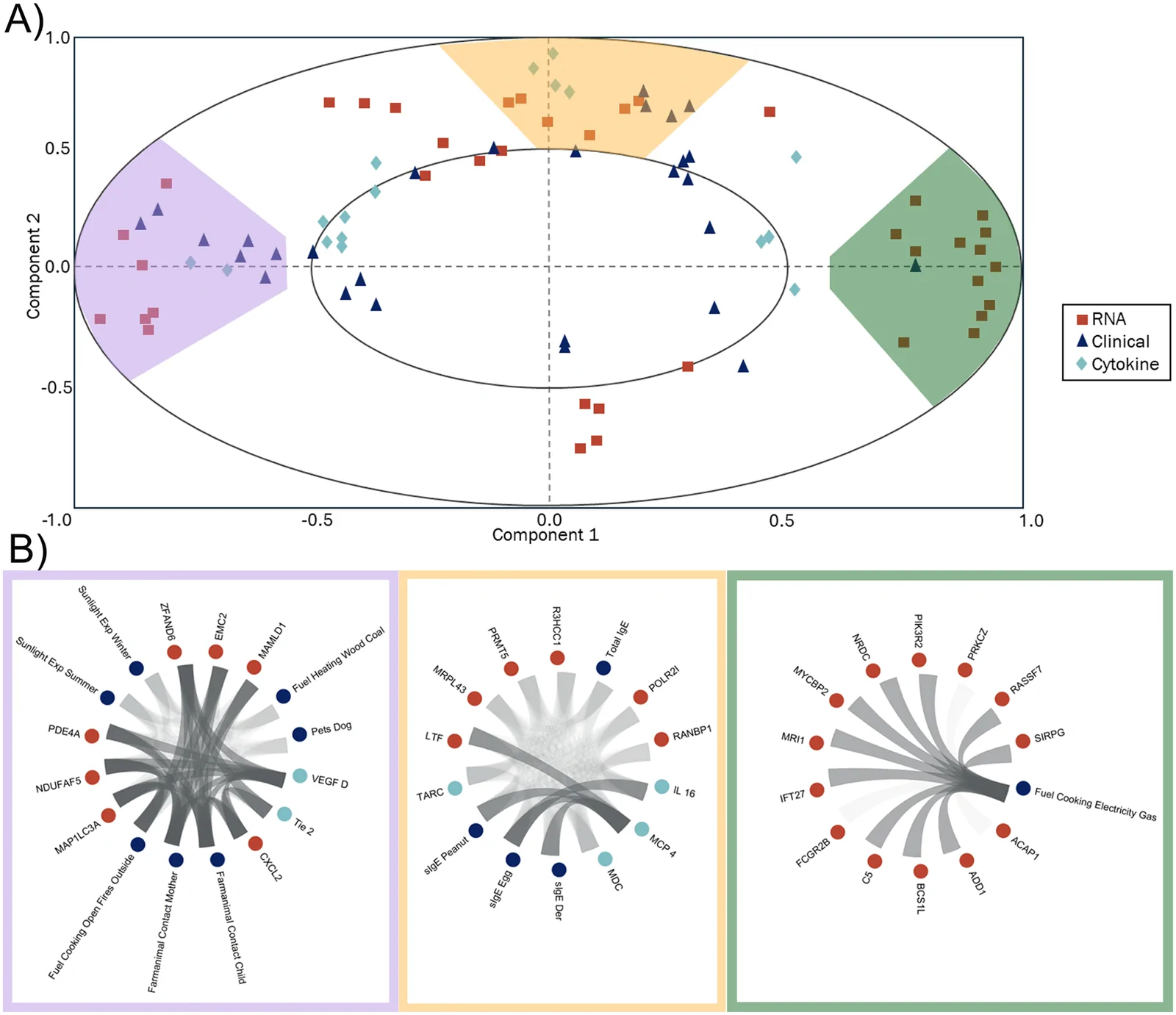
Correlations among features across datasets displayed in a circle plot reveal three distinct clusters. **A)** Circle plot showing correlations between variables from the RF560-selected transcripts (red squares), the questionnaire (blue triangles), the cytokine (cyan diamonds), and the antibody (black circles) datasets. The angles between two vectors drawn to the variable point from the origin show the correlation score (<90° = positive correlation, >90° = negative correlation). The distance from the origin indicates the importance of a variable for the respective component, with the inner circle indicating a correlation score threshold of 0.5. The areas highlighted in violet, orange, or green indicate clusters of correlated variables. **B)** Circos plots of the correlated features in the violet, orange, or green clusters, respectively.

Finally, we used the DIABLO model to investigate how the variables from the four different datasets interact with each other. As depicted in the correlation circle plot in Fig 5A, three distinct clusters of correlated features can be identified, highlighted by the violet, orange, and green areas. The violet cluster (Fig 5B) represents a HC protective cluster. It features the cytokines VGEF D and Tie 2, which contributed most to the prediction of HC in the first component of the DIABLO model for the cytokine data (Fig 4B). These cytokines were correlated with the clinical questionnaire features exposure to sunlight, contact with farm animals, and keeping a dog, which contributed most to the prediction of HC in the first component of the DIABLO model (Fig 4A) and are reported to be protective [29–32]. A network analysis of the six correlated features from the RNA-Seq dataset using STRING [33] resulted in the identification of a subcluster comprising ZFAND6 and MAP1LC3A, which was associated with the UniProt keywords autophagy, protein transport and Ubl conjugation pathway (Fig Q in S1 Appendix).

In contrast, the orange cluster was associated with AD, as it contains the cytokines TARC, MCP4, and IL-16, which are the cytokines contributing to the prediction of AD in the second component of the cytokine DIABLO model (Fig 4B), as well as the levels of total IgE antibodies and of specific IgE antibodies against peanuts, egg and house dust mites (Fig 4A). Submitting the genes of the orange cluster to a network analysis in STRING [33] resulted in three different sub-clusters). One of these sub-clusters primarily comprises nuclear proteins involved in transcription and chromatin organisation, while another sub-cluster, centred around LTF, shows enrichment for the Reactome pathways *Antimicrobioal peptides* and *Innate immune system* (Fig R in S1 Appendix)*.*

The green cluster highlighted in the circle plot in Fig 5A was also correlated with AD. It contains the environmental variable to use electricity or gas as fuel for cooking (Fig 5B), which is the variable that contributes most to the prediction of AD in the first component of the DIABLO model of the clinical questionnaire data (Fig 4A). Of the 13 transcript features in the green cluster, the 9 with a FC of at least 1.08 were considered a signature and subjected to a similarity search against the human mRNASeq dataset in Genevestigator [34] to identify comparisons between conditions or treatments that yield similar transcript signatures. Interestingly, the highest similarity was observed in peripheral blood samples from patients with moderate to severe AD, either untreated or after three months of treatment with Dupilumab, a monoclonal antibody that blocks IL-4 and IL-13 and is used to treat AD. In that study, clinical improvement following Dupilumab administration was accompanied by a decrease in innate immune responses and an increase in B cell and natural killer cell activation [35]. The dataset with the second-highest similarity score was from an asthma study that compared peripheral blood samples collected at baseline and between four and six days after the onset of cold symptoms, without progression to exacerbation [36]. These findings underscore the importance of this gene signature in allergic diseases.

## 4. Discussion

In this study, we performed a data-driven, multimodal analysis integrating questionnaire data on living and health conditions, plasma antibody and cytokine measurements, and transcriptome data from healthy AmaXhosa children and children with AD living in rural and urban South Africa. Explainable ML was applied to the combined questionnaire and antibody data to identify the features most strongly associated with AD. The most prominent predictors of AD in the combined questionnaire and antibody data were the levels of total and allergen-specific IgE antibodies. It is well established that patients with AD often exhibit elevated total and specific IgE levels as a consequence of sensitisation to multiple allergens through a defective epidermal barrier [37]. In addition, it was recently shown that skin damage-derived signals alone can be sufficient to initiate humoral immune responses to spatially unlinked antigens [38]. Therefore, total and specific IgE are useful biomarkers of sensitisation patterns that may reflect specific AD subtypes associated with atopic comorbidities and could potentially predict the response to targeted therapies, such as treatment with Omalizumab [39–43]. This sensitisation pattern might be linked to the eczematous skin lesions that allow easier penetration of allergens [44,45].

Beyond the identification of individual features associated with AD, integration of the questionnaire and antibody data with the selected set of relevant transcripts and cytokine data enabled the identification of three multimodal susceptibility and protective clusters. One AD susceptibility cluster linked allergen-specific and total IgE antibody levels with increased expression of LTF. LTF encodes Lactotransferrin, a multifunctional glycoprotein that plays a prominent role in immune regulation and in antimicrobial defence. Increased LTF expression may therefore be associated with epithelial barrier damage and the altered skin microbiome in AD [42,46]. The cytokines associated with this susceptibility cluster interestingly comprise TARC and MCP-4, which both contribute to the prediction of AD in component 1 of the clinical questionnaire DIABLO model. Increased levels of TARC and of MCP-4 in the blood of paediatric AD patients has been reported previously [47,48]. MCP-4, also known as CCL13, can bind to the receptors CCR1, CCR2, CCR3, CCR5, and CCR11, and is a chemoattractant for monocytes, T cells, immature dendritic cells and eosinophils [49]. Corresponding with the increased levels of MCP-4, also the blood monocyte counts were found to be increased in AD and contributing to the prediction of AD in the first component of the clinical questionnaire DIABLO model. The other susceptibility cluster consisted of 13 transcript features and one environmental variable, namely the use of electricity or gas as fuel for cooking. In general, the risk of skin diseases, particularly AD in infants and schoolchildren, increases with higher concentrations of particulate matter [50]. Moreover, smoke exposure has been associated with epigenetic modifications at 133 disease-relevant gene loci, increased memory CD8+ T cells, and elevated levels of activation and chemokine receptor biomarkers [51]. The identification of this feature in the susceptibility cluster was therefore contrary to expectations, as the use of gas or electricity is generally assumed to generate lower levels of particulate matter than fuels such as wood, coal, or paraffin. However, this finding may reflect underlying differences between the rural cohorts: the rural HC children were from a coastal area, whereas the rural AD children were from a more inland rural region with different lifestyle factors that may predispose individuals to AD. Interestingly, a search of the Genevestigator database [34] for experiments with a similar transcript signature to that found in this susceptibility cluster identified studies investigating AD or asthma. This finding further corroborates the significance and specificity of the identified features for AD.

In contrast, the protective DIABLO cluster includes several environmental exposure variables that are significantly higher in rural areas compared to urban conditions and are protective against AD. These variables include keeping dogs, contact with farm animals, and exposure to sunlight, which may indicate longer periods of time outdoors and exposure to biodiverse environments and increased vitamin D levels [8,15,29–32]. The genes in the protective DIABLO cluster that are correlated with these environmental variables comprise ZFAND6, which is a subunit of a TRAF2-cIAP E3 ubiquitin ligase complex and thereby links to the regulation of tumour necrosis factor (TNF)-induced NF-κB signalling [52], and MAP1LC3A that plays a role in the ubiquitin conjugation system and in autophagy, which was shown to influence the pathogenesis of a variety of inflammatory diseases [53]. These findings underscore the significant interactions between the immune system and the living environment, which play a crucial role in modulating the risk of atopic sensitisation and AD.

Several limitations of this study should be noted. First, the ML approaches used here were not intended to produce a deployable predictive model for clinical use. Rather, they were applied as an explainable AI framework to explore the underlying structure of the data and to generate mechanistic insights into the pathogenesis of AD. Second, although our findings are based on a well-characterised paediatric AD cohort, further validation in an independent cohort would be a valuable next step to confirm the generalisability of the identified disease markers and to support the findings of the current study.

In summary, the use of ML in comprehensively analysing complex datasets including the clinical questionnaire and transcriptomic datasets, as well as in integrated analysis using DIABLO, allowed for the identification of both known and novel susceptibility and protective factors for AD, as well as unknown interactions between these factors. In analysing the clinical and transcriptomic data, ML was more efficient in uncovering significant features that differentiate AD from HC, despite the predominant environmental variable. Calculating SHAP values for the questionnaire and antibody data further enhanced the explainability of the ML output. For the transcriptomics data, identifying the most biologically relevant ML-derived feature list facilitated downstream integration with the questionnaire, antibody and cytokine datasets, which contained considerably fewer features. Correlation analyses ultimately enabled the identification of features contributing to the prediction of HC or AD. This aligns with previous reports showing that multi-omics data integration can uncover more information about implicated molecules and pathways than single-omics approaches [3,54]. It also corresponds with the assessment of the 4th Davos Declaration, which emphasises that multidimensional analyses of environmental factors, genetic predisposition, and changes in the skin microbiome are crucial for developing preventive strategies, designing personalised therapies, and understanding geographical variations in AD [1]. The strength of ML in analysing complex, multi-modular datasets lies therefore in its ability to reveal intricate patterns and relationships that would otherwise remain undetected.

## Supporting information

S1 Appendix Supporting information file comprising the following supporting tables and figures. **Table A.** Description of the data. HC: healthy control, AD: atopic dermatitis. **Table B.** List of the cytokines included in the analyses. The columns ‘Values AD’ and ‘Values HC’ indicate the number of non-NA entries and columns ‘Distribution AD’ and ‘Distribution HC’ give the median value with the interquartile range (IQR) for AD and HC, respectively. Statistical significance for the difference between AD and HC was calculated with a Mann–Whitney test, and the resulting *p*-values were corrected for multiple testing with Bonferroni. **Table C.** List of features in the pre-processed clinical dataset corresponding to the combined questionnaire and antibody data. The respective data type (num = numerical, cat = categorical) and whether the feature was used in the machine learning (exclude = 0) or not (exclude = 1) are indicated. The columns ‘Values AD’ and ‘Values HC’ indicate the number of non-NA entries for numerical features, and the number of positive entries over the number of non-NA entries for categorical features for AD and HC, respectively. Columns ‘Distribution AD’ and ‘Distribution HC’ give the median value with the interquartile range (IQR) for numerical features, and the percentage of positive entries over the number of non-NA entries for categorical features. For categorical features, statistical significance for the difference between AD and HC was calculated with a hypergeometric test, while a Mann–Whitney test was used for numerical features. The resulting *p*-values were corrected for multiple testing with Bonferroni. **Table D.** The occurrence of different features in the top three models of each ML algorithm for the 18 features identified in all three best-performing Random Forest models. **Table E.** Number of significantly changing transcripts in the two comparisons: rural versus urban, and AD versus HC. Number of transcripts with FDR < 0.05 and abs(fold change) > 1.5 in the respective comparison; HC: healthy control, AD: atopic dermatitis. **Fig A.** Venn diagram showing the overlap of samples with patient information, antibody data, RNA-Seq data, and quantitative cytokine data. **Fig B.** Classification metrics of retrained Model 52 (M52) on the entire dataset, showing cross-validation (CV) and test set performance. **Fig C.** Importance of the hyperparameter space variables (model, scaler, number of selected features, imputation using MICE (initial and after iteration), and quasi-constant features) for the objective function. **Fig D.** Correlation scores between the RNA-Seq, the clinical questionnaire and antibody data, and the cytokine datasets (HC samples in orange, AD samples in blue). The clinical dataset corresponds to the combined questionnaire and antibody data. **Fig E.** Projections of samples in the first and second DIABLO components (HC samples in orange, AD samples in blue). **Fig F.** Jaccard score for the overlap between the feature lists selected by the best 3 models of each ML algorithm. **Fig G.** Mean SHAP values for the 18 features selected by the Random Forest model M52, applied to the combined questionnaire and antibody data, showing their impact on the target prediction. **Fig H**. SHAP dependence plot for the levels of specific IgE antibodies against hen egg. **Fig I.** SHAP dependence plot for the levels of specific IgE antibodies against fish. **Fig J.** SHAP dependence plot for the levels of specific IgE antibodies against milk. **Fig K.** SHAP dependence plot for the total levels of IgE antibodies. **Fig L.** SHAP dependence plot for the levels of specific IgG4 antibodies against house dust mites. **Fig M.** PCA analysis of the RNA-Seq data with the Principal Components (PCs) 1–3 (green: rural – AD; grey: rural – HC; red: urban – AD; blue: urban – HC). **Fig N.** Correlation of the fold changes of the DEGs identified in one comparison with the fold changes of these genes in the respective other comparison. A) Correlation of the fold changes of the genes identified with FDR < 0.05 and abs(fold change)> log2(1.5) in the comparison AD versus HC in urban samples. B) Correlation of the fold changes of the genes identified with FDR < 0.05 and abs(fold change)> log2(1.5) in the comparison AD versus HC in rural samples. **Fig O.** Classification performance metrics of the four different ML methods. **Fig P.** Assessment of the overlap between selected feature lists from the four different ML Methods and the differential gene expression (DGE) lists using Overlap Coefficient, Jaccard Coefficient, and Sørensen-Dice Coefficient. **Fig Q.** Genes in the violet DIABLO cluster subjected to network analysis and k-means subclustering in STRING, highlighting the red subcluster. A) The genes from the violet DIABLO cluster subjected to a network analysis in STRING (Szklarczyk and colleagues, 2023), followed by a k-means clustering with the distinct subclusters coloured in red, violet, cyan, yellow, and green. B) The red subcluster from A) features ZFAND6 and MAP1LC3A. The nodes are coloured according to their UniProt annotated keywords *Autophagy* (green), *Protein transport* (blue), and *Ubl conjugation pathway* (red). **Fig R.** Genes in the orange DIABLO cluster subjected to network analysis and k-means subclustering in STRING, highlighting the red and green subclusters. A) The genes from the orange DIABLO cluster subjected to a network analysis in STRING (Szklarczyk and colleagues, 2023) with the interaction sources text mining, experiments, databases and co-expression with a maximum of 20 interactors in the 1st shell, followed by a k-means clustering with the distinct subclusters coloured in red, blue, and green. B) The nodes in the red subcluster from A) are coloured according to the functional enrichment terms: Subcellular localisation: *Nucleus* (yellow); GO Biological Process: *Transcription, DNA-templated* (red) and *Chromatin organisation* (purple); Local network cluster: *Methylosome* (green). C) The nodes in the green subcluster from A) are coloured according to the functional enrichment terms: GO Biological Process: *defense response to bacterium* (green); Reactome pathways: *Antimicrobial peptides* (red) and *Innate immune system* (purple).
(PDF)

S1 File Questionnaire (Appendix IV of the study protocol). (PDF)

S2 File SOS-ALL Machine Learning methods used to analyse the clinical dataset corresponding to the combined questionnaire and antibody data. (PDF)

S3 File Markdown document providing an overview of the data analysis carried out on the SOS-ALL project data. (PDF)

S1 Table Transcripts contained in the 11 co-expressed gene clusters shown in Fig 3, identified using weighted gene co-expression network analysis (WGCNA). For each transcript, its ENSEMBL ID and gene name are provided, and a “1” in the column corresponding to the cluster colour used in Fig 3 indicates that the transcript belongs to that cluster.
(CSV)

S1 Checklist Inclusivity in Global Research. (PDF)

S2 Checklist TRIPOD+AI [28]; the checklist was downloaded from the TRIPOD Statement website (https://www.tripod-statement.org/wp-content/uploads/2019/12/TRIPODAI_checklist.pdf) and is distributed under the Creative Commons Attribution 4.0 International (CC BY 4.0) licence (https://creativecommons.org/licenses/by/4.0/). (PDF)

## Abbreviations

AD: atopic dermatitis
CV: cross-validation
DIABLO: Data Integration Analysis for Biomarker discovery using Latent cOmponents
DGE: differential gene expression
FC: fold change
FDR: false discovery rate
GNB: Gaussian Naive Bayes
GOBP: Gene Ontology Biological Process
HC: healthy control
ML: machine learning
MLP: Multi Level Perceptron
PBMCs: peripheral blood mononuclear cells
PCs: Principal Components
RF: Random Forest
RNA-Seq: RNA sequencing
SCORAD: SCoring Atopic Dermatitis
SHAP: SHapley Additive exPlanation
SPT: skin prick test
TNF: tumour necrosis factor
TPE: Tree-structured Parzen Estimator
WGCNA: weighted co-expression network analysis
XGBOOST: eXtreme Gradient Boosting.
