# Supplementary material for: Multimodal protective and susceptibility clusters in paediatric atopic dermatitis: A machine learning-based, data-driven observational study
Source: PLoS Med. 2026 Sep 8;23(9):e1004917. doi: 10.1371/journal.pmed.1004917 (PMC13552787; doi:10.1371/journal.pmed.1004917)
Supplement: Supplementary file 1 [file pmed.1004917.s001.pdf]

Supporting Information S1 Appendix

**Table A.** Description of the data.	Page 1
**Table B.** List of the cytokines included in the analyses.	Page 2
**Table C.** List of features in the pre-processed clinical dataset corresponding to the combined questionnaire and antibody data.	Pages 3-5
**Table D.** The occurrence of different features in the top three models of each ML algorithm for the 18 features identified in all three best-performing Random Forest models.	Page 6
**Table E**. Number of significantly changing transcripts in the two comparisons: rural versus urban, and AD versus HC.	Page 7
**Fig. A** Venn diagram showing the overlap of samples with patient information, antibody data, RNA-Seq data, and quantitative cytokine data.	Page 8
**Fig. B** Classification metrics of retrained Model 52 (M52) on the entire dataset, Showing cross-validation (CV) and test set performance.	Page 9
**Fig. C** Importance of the hyperparameter space variables (model, scaler, number of selected features, imputation using MICE (initial and after iteration), and quasi-constant features) for the objective function.	Page 10
**Fig. D** Correlation scores between the RNA-Seq, the clinical, and the cytokine datasets.	Page 11
**Fig. E** Projections of samples in the first and second DIABLO components.	Page 12
**Fig. F** Jaccard score for the overlap between the feature lists selected by the best 3 models of each ML algorithm.	Page 13
**Fig. G** Mean SHAP values for the 18 features selected by the Random Forest model M52, applied to the combined questionnaire and antibody data, showing their impact on the target prediction.	Page 14
**Fig. H** SHAP dependence plot for the levels of specific IgE antibodies against hen egg.	Page 15
**Fig. I** SHAP dependence plot for the levels of specific IgE antibodies against fish.	Page 16
**Fig. J** SHAP dependence plot for the levels of specific IgE antibodies against milk.	Page 17
**Fig. K** SHAP dependence plot for the total levels of IgE antibodies.	Page 18
**Fig. L** SHAP dependence plot for the levels of specific IgG4 antibodies against house dust mites.	Page 19
**Fig. M** PCA analysis of the RNA-Seq data with the Principal Components (PCs) 1-3.	Page 20
**Fig. N** Correlation of the fold changes of the DEGs identified in one comparison with the fold changes of these genes in the respective other comparison.	Page 21
**Fig. O** Classification performance metrics of the four different ML methods.	Page 22
**Fig. P** Assessment of the overlap between selected feature lists from the four different ML Methods and the DGE lists using Overlap Coefficient, Jaccard Coefficient, and Sørensen-Dice Coefficient.	Page 23
**Fig. Q** Genes in the violet DIABLO cluster subjected to network analysis and k-means subclustering in STRING, highlighting the red subcluster.	Page 24
**Fig. R** Genes in the orange DIABLO cluster subjected to network analysis and k-means subclustering in STRING, highlighting the red and green subclusters.	Page 25

Table A. Description of the data. HC: healthy control, AD: atopic dermatitis

Feature	samples AD	samples HC	Total
Location rural	60	52	112
Location urban	56	49	105
**Total questionnaire**	116	101	**217**
Antibody rural	46	39	85
Antibody urban	32	35	67
**Total Antibody**	78	74	**152**
RNA-Seq rural	45	44	89
RNA-Seq urban	31	29	60
**Total RNA-Seq**	76	73	**149**
Cytokine rural	46	43	89
Cytokine urban	32	38	70
**Total Cytokine**	78	81	**159**

Table B. List of the cytokines included in the analyses. The columns 'Values AD' and 'Values HC' indicate the number of non-NA entries and columns 'Distribution AD' and 'Distribution HC' give the median value with the interquartile range (IQR) for AD and HC, respectively. Statistical significance for the difference between AD and HC was calculated with a Mann-Whitney test, and the resulting p-values were corrected for multiple testing with Bonferroni.

Cytokine	Values AD	Values HC	Distribution AD median [IQR]	Distribution HC median [IQR]	pvalue	Adjusted pvalue
CRP	78	81	4617[1413,16096]	4725 [1560,13352]	0.971	1
ICAM.1	78	81	2061 [1693,2566]	2097 [1881,2375]	0.314	1
SAA	78	81	6545 [1772,37614]	5041 [1579,29672]	0.703	1
VCAM.1	78	81	2501 [2122,3128]	2627 [2207,3115]	0.34	1
Eotaxin	78	81	29.9 [15.4,72.1]	22.3 [13.9,59.8]	0.193	1
**Eotaxin.3**	78	80	10.0 [6.13,25.4]	4.65 [2.45,12.6]	6.634E-05	3.052E-03
IP.10	77	81	74.1 [49.0,124]	64.0 [46.5,112]	0.267	1
MCP.1	78	81	35.0 [23.6,67.6]	38.7 [22.9,54.0]	0.56	1
MDC	78	81	361 [288,493]	355 [295,419]	0.488	1
MIP.1alpha	77	81	20.4 [8.33,52.6]	17.9 [8.25,46.2]	0.671	1
MIP.1beta	78	81	75.7 [28.9,206]	45.2 [26.1,115]	5.519E-02	1
**TARC**	78	81	56.4 [36.8,104]	34.4 [20.7,51.8]	2.336E-06	1.075E-04
MCP.4	78	81	44.6 [30.7,65.1]	33.6 [26.3,44.6]	5.202E-03	0.239
GM.CSF	70	78	0.139 [0.0973,0.197]	0.136 [0.0883,0.179]	0.608	1
IL.12.IL.23p40	78	81	71.7 [54.3,85.0]	70.3 [55.7,89.9]	0.44	1
IL.15	78	81	0.432 [0.321,0.562]	0.447 [0.343,0.580]	0.486	1
IL.16	78	81	74.8 [60.0,96.8]	66.1 [54.4,78.0]	2.480E-02	1
IL.17A	78	81	3.76 [2.83,6.02]	5.32 [3.36,8.49]	3.220E-03	0.148
IL.1alpha	68	69	0.250 [0.121,0.332]	0.250 [0.155,0.351]	0.363	1
IL.5	76	79	0.349 [0.2209,0.552]	0.334 [0.233,0.624]	0.892	1
IL.7	78	81	3.09 [2.57,3.86]	3.05 [2.62,3.72]	0.76	1
TNF.beta	78	81	0.127 [0.092,0.182]	0.156 [0.102,0.212]	9.270E-02	1
VEGF_1	78	81	25.8 [19.8,37.6]	25.1 [15.5,37.5]	0.26	1
IFN.gamma	78	81	4.97 [2.50,11.9]	3.82 [2.24,6.44]	0.115	1
IL.10	78	81	0.370 [0.216,0.757]	0.322 [0.233,0.516]	0.162	1
IL.12p70	63	73	0.0638 [0.0355,0.160]	0.0702 [0.0422,0.123]	0.724	1
IL.13	71	71	3.23 [1.46,6.74]	2.25 [1.69,3.05]	2.010E-02	0.927
IL.1beta	65	69	0.169 [0.0442,1.13]	0.0512 [0.0247,0.177]	2.160E-03	9.920E-02
IL.2	32	46	0.132 [0.0491,4.50]	0.0986 [0.0596,0.197]	0.366	1
IL.4	12	8	2.02 [0.0639,3.79]	0.0304 [0.0106,0.0819]	7.303E-03	0.336
IL.6	73	69	2.95 [0.849,9.33]	2.26 [0.869,5.932]	0.321	1
IL.8	78	81	32.7 [9.31,171]	24.6 [5.37,81.5]	0.0697	1
TNF.alpha	78	80	2.10 [1.42,3.56]	1.71 [1.31,2.51]	0.0316	1
bFGF	78	81	3.58 [1.73,32.0]	3.2 [1.69,19.5]	0.711	1
Flt.1	78	81	31.3 [19.2,45.1]	28.4 [19.3,47.0]	0.61	1
PlGF	78	81	1.42 [0.954,32.6]	1.61 [1.06,27.2]	0.677	1
Tie.2	77	79	145 [39.6,185]	146 [25.9,182]	0.743	1
VEGF_2	78	81	72.6 [28.3,125]	57.3 [24.1,101]	0.0951	1
VEGF.C	78	81	35.6 [20.8,69.3]	31.0 [16.6,59.9]	0.318	1
VEGF.D	77	79	123 [30.1,163]	129 [30.7,178]	0.439	1
IL.21	18	27	0.229 [0.141,0.346]	0.307 [0.233,0.438]	0.129	1
IL.22	78	81	1.37 [0.723,2.21]	1.66 [0.923,2.23]	0.321	1
IL.23	31	46	0.208 [0.148,0.533]	0.270 [0.0918,0.435]	0.655	1
IL.27	78	81	285 [230,365]	314 [272,419]	0.0169	0.776
IL.31	74	79	0.0684 [0.0472,0.1]	0.0733 [0.0612,0.105]	0.169	1
MIP.3alpha	77	81	9.87 [6.93,14.3]	9.89 [6.98,13.8]	0.746	1

Table C. **List of features in the pre-processed clinical dataset corresponding to the combined questionnaire and antibody data.** The respective data type (num = numerical, cat = categorical) and whether the feature was used in the machine learning (exclude = 0) or not (exclude = 1) are indicated. The columns 'Values AD' and 'Values HC' indicate the number of non-NA entries for numerical features, and the number of positive entries over the number of non-NA entries for categorical features for AD and HC, respectively. Columns 'Distribution AD' and 'Distribution HC' give the median value with the interquartile range (IQR) for numerical features, and the percentage of positive entries over the number of non-NA entries for categorical features. For categorical features, statistical significance for the difference between AD and HC was calculated with a hypergeometric test, while a Mann-Whitney test was used for numerical features. The resulting p-values were corrected for multiple testing with Bonferroni.

Feature	Type	Exclude	Values AD	Values HC	Distribution AD median [IQR] or %	Distribution HC median [IQR] or %	pvalue	Adjusted pvalue
enrolment_age	num	0	116	101	21.5 [16,27]	22 [17,29]	0.44	1
gender	cat	0	64 / 115	56 / 101	55.65%	55.45%	0.54	1
vaccination_status	cat	1	104 / 114	97 / 101	91.23%	96.04%	0.96	1
paracetamol_exposure	cat	0	110 / 114	94 / 101	96.49%	93.07%	0.2	1
paracetamol_exposure_age	num	0	109	92	4 [3,6]	6 [3,7.5]	0.043	1
paracetamol_exposure_time	cat	0	74 / 114	67 / 100	64.91%	67%	0.68	1
childhood_inf_mumps	cat	0	0 / 114	2 / 101	0%	1.98%	1	1
childhood_inf_chickenpox	cat	0	4 / 114	7 / 101	3.51%	6.93%	0.93	1
childhood_inf_giandular_fever	cat	0	0 / 114	2 / 101	0%	1.98%	1	1
childhood_inf_tuberculosis	cat	0	2 / 114	3 / 101	1.75%	2.97%	0.85	1
childhood_inf_other	cat	0	1 / 102	2 / 101	0.98%	1.98%	0.88	1
antibiotic_exposure	cat	0	87 / 113	75 / 101	76.99%	74.26%	0.38	1
antibiotic_exposure_age	num	1	85	75	6 [3,7]	6 [3.5,8]	0.28	1
antibiotic_exposure_courses	cat	0	24 / 109	25 / 99	22.02%	25.25%	0.76	1
antibiotic_exposure_last2months	cat	0	42 / 112	33 / 101	37.50%	32.67%	0.28	1
anti_helminthic_exposure	cat	0	95 / 111	88 / 101	85.59%	87.13%	0.7	1
anti_helminthic_exposure_age	num	0	94	88	12 [12,12]	12 [12,12]	0.14	1
anti_helminthic_exposure_last	num	0	95	88	18 [12,24]	18 [12,24]	0.76	1
anti_helminthic_exposure_yearly	cat	0	76 / 111	75 / 101	68.47%	74.26%	0.86	1
delivery_mode vaginal	cat	0	75 / 114	70 / 101	65.79%	69.31%	0.76	1
sunlight_exp_winter	num	0	76	62	2 [1,4]	3.5 [2,5]	0.0049	0.61
sunlight_exp_summer	num	0	73	55	3 [2,6]	6 [3.5,8]	0.0014	0.18
peanuts_mother_exposure_regularity	cat	0	73 / 111	55 / 100	65.77%	55%	0.072	1
peanuts_mother_exposure_avoidance	cat	0	12 / 111	16 / 99	10.81%	16.16%	0.91	1
breastfeeding_ever	cat	0	92 / 115	86 / 101	80%	85.15%	0.88	1
breastfeeding_uptoage_age_complete	num	0	104	89	3 [0.5,6]	3 [1,6]	0.61	1
breastfeeding_uptoage_age_stop	num	0	106	92	6 [1,12]	6 [2,12]	0.92	1
formula_age_introduction	num	0	106	99	3 [0,6]	3 [0,6]	0.92	1
solidfood_age_introduction	num	0	115	101	5 [4,6]	6 [4,6]	0.46	1
solidfood_which_first	cat	1	NA	NA	NA	NA	NA	NA
peanuts_exposure	cat	0	71 / 114	60 / 101	62.28%	59.41%	0.39	1
peanuts_exposure_age_first	num	1	70	60	12 [10,18]	12 [9,19]	0.84	1
peanuts_exposure_regularity	cat	1	60 / 71	45 / 61	84.51%	73.77%	0.095	1
othernuts_exposure	cat	0	36 / 114	14 / 101	31.58%	13.86%	0.0016	0.2
othernuts_exposure_age_first	num	1	36	14	12 [11,23.5]	12 [12,14]	0.37	1
othernuts_exposure_regularity	cat	1	32 / 36	Dez 14	88.89%	85.71%	0.55	1
dairyproducts_exposure	cat	0	112 / 114	101 / 101	98.25%	100%	1	1
dairyproducts_exposure_age_first	num	0	112	101	7 [6,9.5]	7 [6,9]	0.61	1
dairyproducts_exposure_regularity	cat	0	109 / 112	100 / 101	97.32%	99.01%	0.93	1
cowmilkformula_exposure	cat	0	108 / 114	97 / 101	94.74%	96.04%	0.78	1
cowmilkformula_exposure_age_first	num	0	99	86	3 [0,6]	3 [1,6]	0.63	1
cowmilkformula_exposure_regularity	cat	0	53 / 107	41 / 97	49.53%	42.27%	0.18	1
henegg_exposure	cat	0	94 / 114	94 / 101	82.46%	93.07%	0.1	1
henegg_exposure_age_first	num	0	92	94	12 [8,12.5]	12 [8,12]	0.74	1
henegg_exposure_regularity	cat	0	63 / 93	88 / 95	67.74%	92.63%	1	1

Feature	Type	Exclude	Values AD	Values HC	Distribution AD median [IQR] or %	Distribution HC median [IQR] or %	pvalue	Adjusted pvalue
wheat_exposure	cat	0	108 / 114	100 / 101	94.74%	99.01%	0.98	1
wheat_exposure_age_first	num	0	108	100	9 [6,12]	10 [6,12]	0.33	1
wheat_exposure_regularity	cat	0	99 / 108	98 / 100	91.67%	98%	0.99	1
fish_exposure	cat	0	77 / 114	80 / 101	67.54%	79.21%	0.98	1
fish_exposure_age_first	num	1	78	80	12 [12,18]	12 [12,17]	0.83	1
fish_exposure_regularity	cat	1	76 / 78	77 / 81	97.44%	95.06%	0.36	1
asthma_ever	cat	0	1 / 116	1 / 101	0.86%	0.99%	0.78	1
hayfever_ever	cat	0	20 / 116	4 / 101	17.24%	3.96%	0.0014	0.17
eczema_ever	cat	1	116 / 116	4 / 101	100%	3.96%	1.21E-57	1.49E-55
eczema_age	num	1	116	4	5 [3,7]	2 [0.75,13.5]	0.28	1
eczema_whodiagnosed	cat	1	NA	NA	NA	NA	NA	NA
medication_any	cat	1	1 / 114	1 / 101	0.88%	0.99%	0.78	1
medication_nasalcorticosteroid	cat	1	5 / 114	1 / 101	4.39%	0.99%	0.14	1
medication_antihistamines	cat	1	99 / 114	14 / 101	86.84%	13.86%	5.06E-29	6.23E-27
medication_steroidcreams	cat	1	109 / 114	2 / 101	95.61%	1.98%	2.90E-52	3.57E-50
medication_oral_which	cat	1	NA	NA	NA	NA	NA	NA
medication_antihistamines_last	cat	1	NA	NA	NA	NA	NA	NA
medicalhistory_comorbidities	cat	1	1 / 114	1 / 101	0.88%	0.99%	0.78	1
household_peoplelivingtogether	num	0	114	101	5 [4,7]	5 [4,8]	0.13	1
household_otherolderchildren	num	0	113	101	1 [0,2]	1 [0,2]	0.2	1
household_otheryoungerchildren	num	0	114	101	0 [0,0]	0 [0,0]	0.61	1
parental_education_level	cat	1	NA	NA	NA	NA	NA	NA
parental_income	cat	1	NA	NA	NA	NA	NA	NA
pets_cat	cat	0	18 / 114	26 / 101	15.79%	25.74%	0.975911	1
pets_dog	cat	0	50 / 114	57 / 101	43.86%	56.44%	0.976121	1
farmanimal_contact_child	cat	0	44 / 113	52 / 101	38.94%	51.49%	0.976238	1
farmanimal_contact_mother	cat	0	49 / 113	51 / 100	43.36%	51%	0.894775	1
cigarettesmoke_numberofpersons	num	0	111	101	0 [0,0.5]	0 [0,1]	0.12	1
cigarettesmoke_duringpregnancy	cat	0	1 / 112	4 / 101	0.89%	3.96%	0.98	1
am_height	num	1	116	101	81 [77,85.5]	84 [79,88]	0.055	0.67
am_weight	num	1	116	101	11.9 [10.5,13.1]	12.2 [10.8,13.6]	0.34	1
am_abd_girth	num	1	113	97	45 [43,48]	44 [41,46]	0.0057	0.7
am_skinfold	num	1	112	98	11 [9,12.5]	10 [8,11]	2.79E-05	0.0034
sp_egg	num	0	87	101	0 [0,0]	0 [0,0]	1.49E-05	0.0018
sp_milk	num	0	109	101	0 [0,0]	0 [0,0]	0.095	1
sp_soy	num	0	109	101	0 [0,0]	0 [0,0]	0.095	1
sp_wheat	num	0	110	101	0 [0,0]	0 [0,0]	0.018	1
sp_fish	num	0	109	101	0 [0,0]	0 [0,0]	NA	NaN
sp_peanut	num	0	96	101	0 [0,0]	0 [0,0]	0.00026	0.032
sp_hazelnut	num	0	107	101	0 [0,0]	0 [0,0]	0.092	1
sp_fresh_peanut	num	0	98	100	0 [0,0]	0 [0,0]	0.0011	0.13
sp_fresh_egg	num	0	88	101	0 [0,10]	0 [0,0]	3.58E-12	4.40E-10
sp_fresh_milk	num	0	105	101	0 [0,0]	0 [0,0]	0.027	1

Feature	Type	Exclude	Values AD	Values HC	Distribution AD median [IQR] or %	Distribution HC median [IQR] or %	pvalue	Adjusted pvalue
sIgE_Der	num	0	78	74	7.9 [0.24,31]	0.086 [0.072,0.12]	8.64E-15	1.06E-12
sIgE_milk	num	0	78	74	0.31 [0.11,1.66]	0.15 [0.051,0.30]	0.0003	0.037
sIgE_fish	num	0	78	74	0.031 [0.022- 0.059]	0.020 [0.011,0.028]	1.58E-07	1.95E-05
sIgE_wheat	num	0	78	74	0.15 [0.096,0.43]	0.090 [0.080,0.11]	2.02E-09	2.49E-07
sIgE_peanut	num	0	78	74	0.24 [0.073,0.83]	0.061 [0.049,0.073]	1.56E-13	1.92E-11
sIgE_soy	num	0	78	74	0.084 [0.042,0.29]	0.036 [0.027- 0.044]	8.17E-10	1.00E-07
sIgE_hazelnut	num	0	78	74	0.051 [0.032,0.10]	0.028 [0.022,0.034]	5.82E-10	7.15E-08
sIgE_egg	num	0	78	74	1.02 [0.17,4.13]	0.045 [0.030,0.11]	6.44E-17	7.92E-15
sIgE_CCD	num	0	78	74	0.028 [0.018,0.054]	0.017 [0.011,0.027]	3.40E-06	0.00042
sIgG4_Der	num	0	78	74	17.1 [11.5,31.9]	11.2 [8.08- 15.8]	5.64E-06	0.00069
sIgG4_fish	num	0	78	74	0.14 [0.001,1.03]	0.001 [0.001,1.20]	0.84	1
sIgG4_wheat	num	0	78	74	16.2 [12.1,32.2]	13.1 [8.98,27.3]	0.02	1
sIgG4_peanut	num	0	78	74	5.79 [3.48,14.6]	4.51 [2.17,6.83]	0.012	1
sIgG4_soy	num	0	78	74	5.36 [3.19,12.3]	4.26 [2.40,6.13]	0.042	1
sIgG4_hazelnut	num	0	78	74	4.30 [3.02,6.34]	3.27 [1.69,5.03]	0.027	1
sIgG4_casein	num	0	78	74	93.7 [51.1,339]	47.6 [23.8,182]	0.0084	1
sIgG4_egg	num	0	78	74	76.7 [18.4,508]	13.40 [5.11,407]	0.0097	1
total_IgE	num	0	78	74	257 [83.9,760]	51.2 [24.7,133]	1.98E-09	2.44E-07
scorad	num	1	15	0	44 [39,56]	NA	NA	NA
bsa	num	1	113	0	11.5 [6,22.5]	NA	NA	NA
stool_katokatz	cat	0	NA	NA	NA	NA	NA	NA
country	cat	0	NA	NA	NA	NA	NA	NA
foodreaction_any	cat	0	15 / 114	2 / 101	13.16%	1.98%	0.0018	0.22
immun_number	num	0	113	100	17 [15,17]	17 [15,17]	0.25	1
familyhistory_mother_eczema	cat	0	9 / 113	1 / 101	7.96%	0.99%	0.015	1
familyhistory_mother_other_allergic_disease	cat	0	20 / 113	9 / 101	17.70%	8.91%	0.046	1
familyhistory_father_eczema	cat	0	5 / 112	0 / 97	4.46%	0%	0.042	1
familyhistory_father_other_allergic_disease	cat	0	16 / 112	3 / 97	14.29%	3.09%	0.0039	0.48
familyhistory_sibling_eczema	cat	1	6 / 45	0 / 45	13.33%	0%	0.013	1
familyhistory_sibling_other_allergic_disease	cat	1	6 / 45	2 / 45	13.33%	4.44%	0.13	1
fuel_cooking_Electricity_Gas	cat	0	112 / 114	60 / 100	98.25%	60%	1.26E-13	1.55E-11
fuel_cooking_Paraffin_Stove	cat	0	6 / 114	31 / 100	5.26%	31%	1	1
fuel_cooking_Open_fires_inside	cat	0	2 / 114	0 / 100	1.75%	0%	0.28	1
fuel_cooking_Open_fires_outside	cat	0	15 / 114	35 / 100	13.16%	35%	1	1
fuel_heating_Electricity	cat	0	25 / 113	14 / 101	22.12%	13.86%	0.082	1
fuel_heating_Gas	cat	0	4 / 113	3 / 101	3.54%	2.97%	0.56	1
fuel_cooking_heating_Paraffin	cat	0	80 / 113	57 / 101	70.80%	56.44%	0.021	1
fuel_heating_Wood_Coal	cat	0	4 / 113	25 / 101	3.54%	24.75%	1	1
fuel_heating_Other	cat	0	3 / 113	7 / 101	2.65%	6.93%	0.97	1
log_blood_counts_wcc	num	0	100	87	2.40 [2.22,2.54]	2.31 [2.13- 2.55]	0.093	1
log_blood_counts_hb	num	0	97	82	2.40 [2.36- 2.45]	2.40 [2.33,2.46]	0.89	1
log_blood_counts_plts	num	0	96	82	5.97 [5.77- 6.18]	5.97 [5.78- 6.16]	0.55	1
log_blood_counts_neutrophils	num	0	94	79	1.24 [0.97,1.65]	1.26 [0.88,1.59]	0.96	1
log_blood_counts_monocytes	num	0	95	79	0.039 [-0.27,0.30]	-0.051[-0.44,0.17]	0.026	1
log_blood_counts_lymphocytes	num	0	94	79	1.76 [1.52- 1.96]	1.63 [1.42,1.91]	0.08	1
log_blood_counts_eosinophils	num	0	3	7	-0.58 [-1.08,-0.10]	-0.92 [-1.50,-0.34]	0.022	1

Table D. The occurrence of different features in the top three models of each ML algorithm for the 18 features identified in all three best-performing Random Forest models.

Feature	Sum	GNB first_3 trial 20	GNB first_3 trial 28	GNB first_3 trial 46	MLP first_3 trial 0	MLP first_3 trial 11	MLP first_3 trial 14	RF first_3 trial 52	RF first_3 trial 73	RF first_3 trial 77	XGBoost first_3 trial 29	XGBoost first_3 trial 34	XGBoost first_3 trial 48
fuel_heating_Wood_Coal	12	1	1	1	1	1	1	1	1	1	1	1	1
total_IgE	12	1	1	1	1	1	1	1	1	1	1	1	1
fuel_cooking_Electricity_Gas	12	1	1	1	1	1	1	1	1	1	1	1	1
fuel_cooking_Paraffin_Stove	12	1	1	1	1	1	1	1	1	1	1	1	1
fuel_cooking_Open_fires_outside	12	1	1	1	1	1	1	1	1	1	1	1	1
sIgE_Der	12	1	1	1	1	1	1	1	1	1	1	1	1
sIgE_fish	12	1	1	1	1	1	1	1	1	1	1	1	1
sIgG4_Der	12	1	1	1	1	1	1	1	1	1	1	1	1
henegg_exposure_regularity	12	1	1	1	1	1	1	1	1	1	1	1	1
hayfever_ever	12	1	1	1	1	1	1	1	1	1	1	1	1
sIgE_milk	11	1	1	1	1	1	1	1	1	1	1	1	0
paracetamol_exposure_age	11	1	1	1	1	1	1	1	1	1	1	1	0
othernuts_exposure	11	1	1	1	1	1	1	1	1	1	1	1	0
foodreaction_any	11	1	1	1	1	1	1	1	1	1	1	1	0
sunlight_exp_summer	10	1	0	1	1	1	1	1	1	1	1	1	0
familyhistory_mother_eczema	10	1	0	1	1	1	1	1	1	1	1	1	0
henegg_exposure	10	1	0	1	1	1	1	1	1	1	1	1	0
sIgE_egg	10	1	0	1	1	1	1	1	1	1	1	1	0

Table E. Number of significantly changing transcripts in the two comparisons: rural versus urban, and AD versus HC. Number of transcripts with FDR < 0.05 and abs(fold change) > 1.5 in the respective comparison; HC: healthy control, AD: atopic dermatitis

	Higher in rural	Higher in urban	Total
Rural-AD vs Urban-AD	2293	2406	4699
Rural-HC vs Urban-HC	1898	1933	3831
Overlap	1568	1571	3139
	Higher in AD	Higher in HC	
Rural-AD vs Rural-HC	9	27	36
Urban-AD vs Urban-HC	54	28	82
Overlap	1	0	1

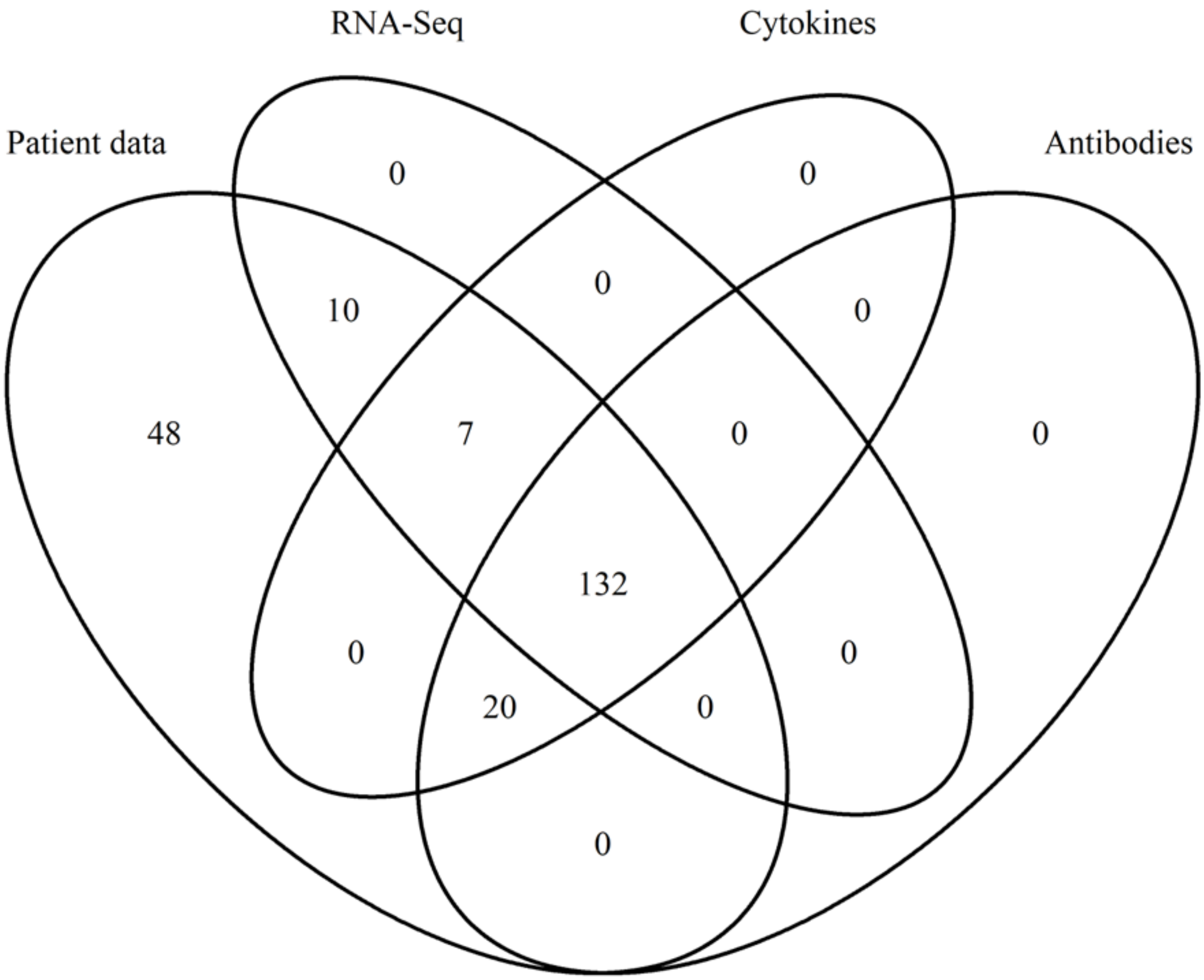

Fig. A Venn diagram showing the overlap of samples with patient information, antibody data, RNA-Seq data, and quantitative cytokine data.

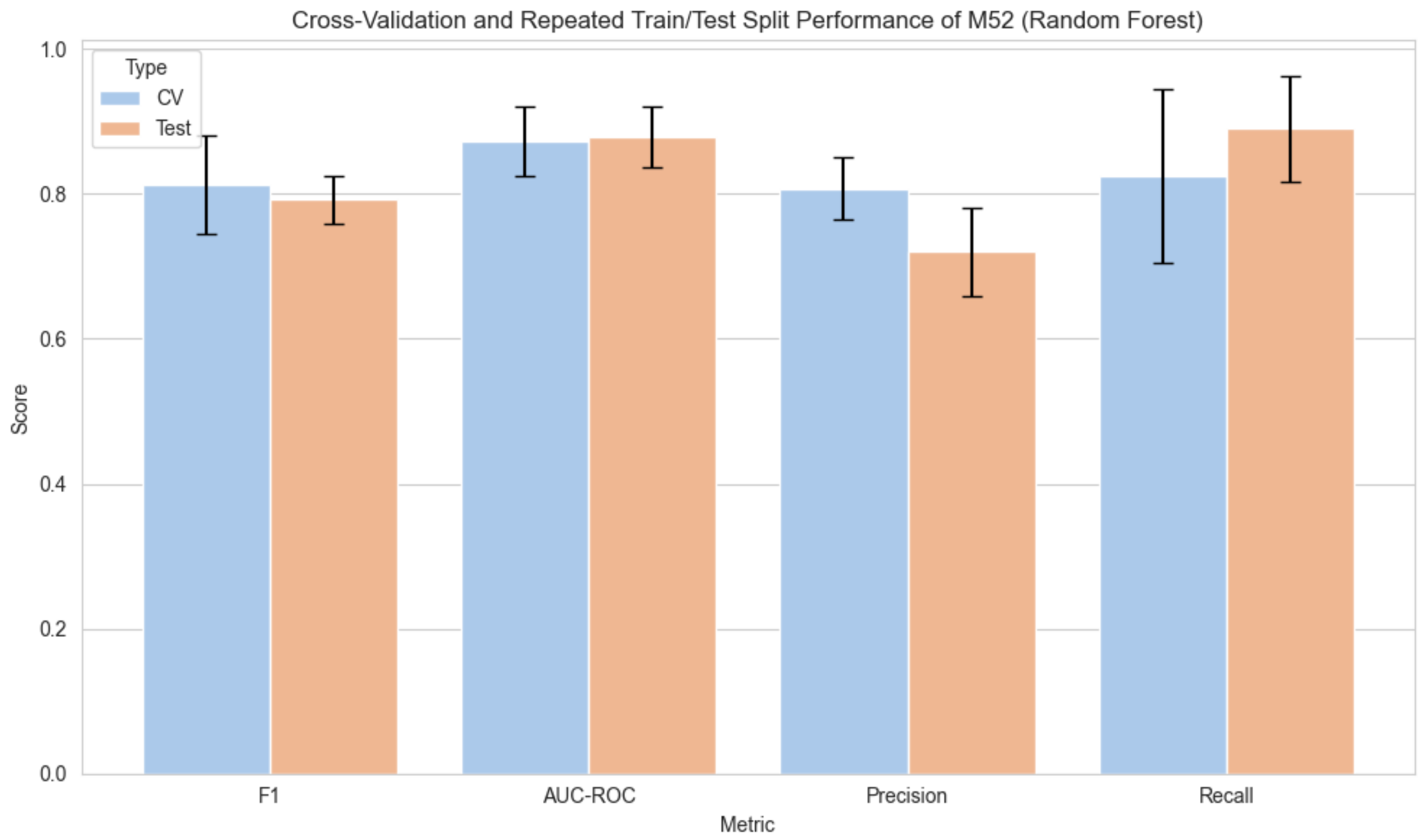

Fig. B Classification metrics of retrained Model 52 (M52) on the entire dataset, showing cross-validation (CV) and test set performance.

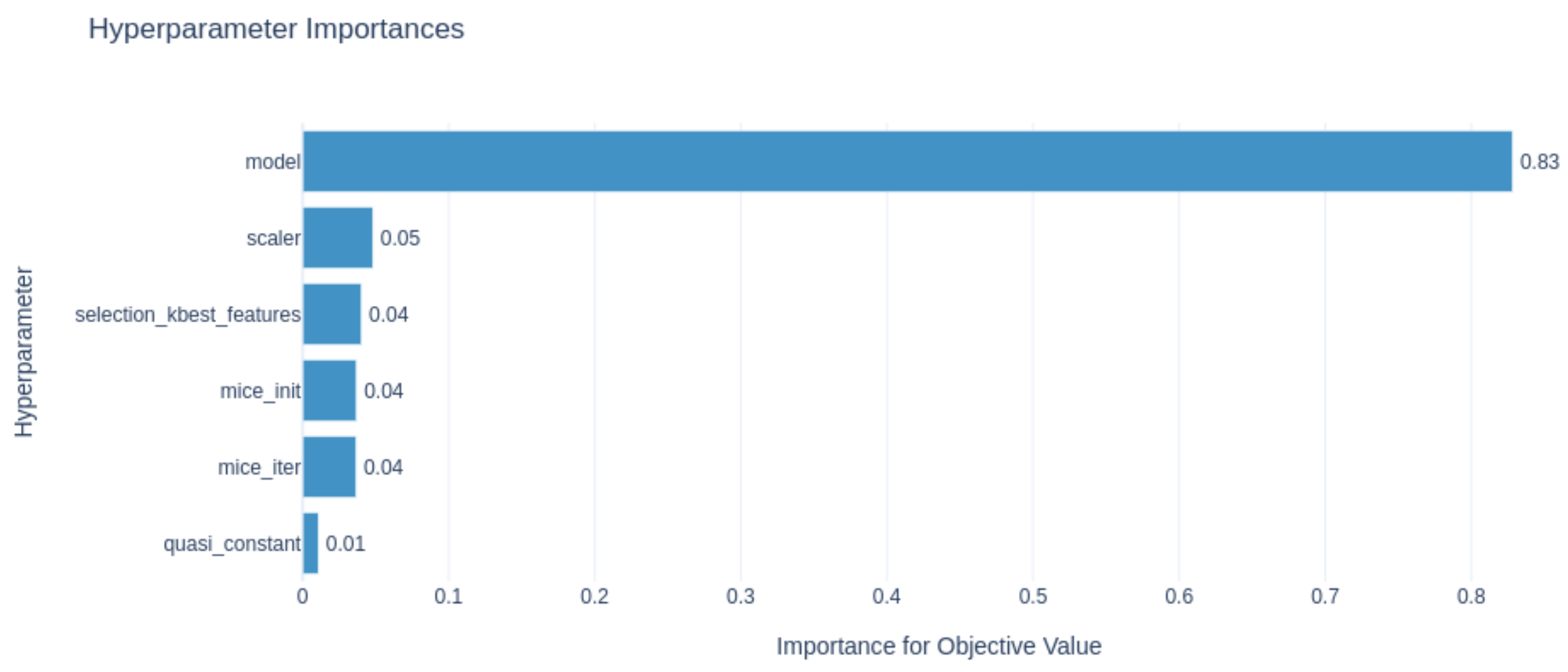

Fig. C Importance of the hyperparameter space variables (model, scaler, number of selected features, imputation using MICE (initial and after iteration), and quasi-constant features) for the objective function.

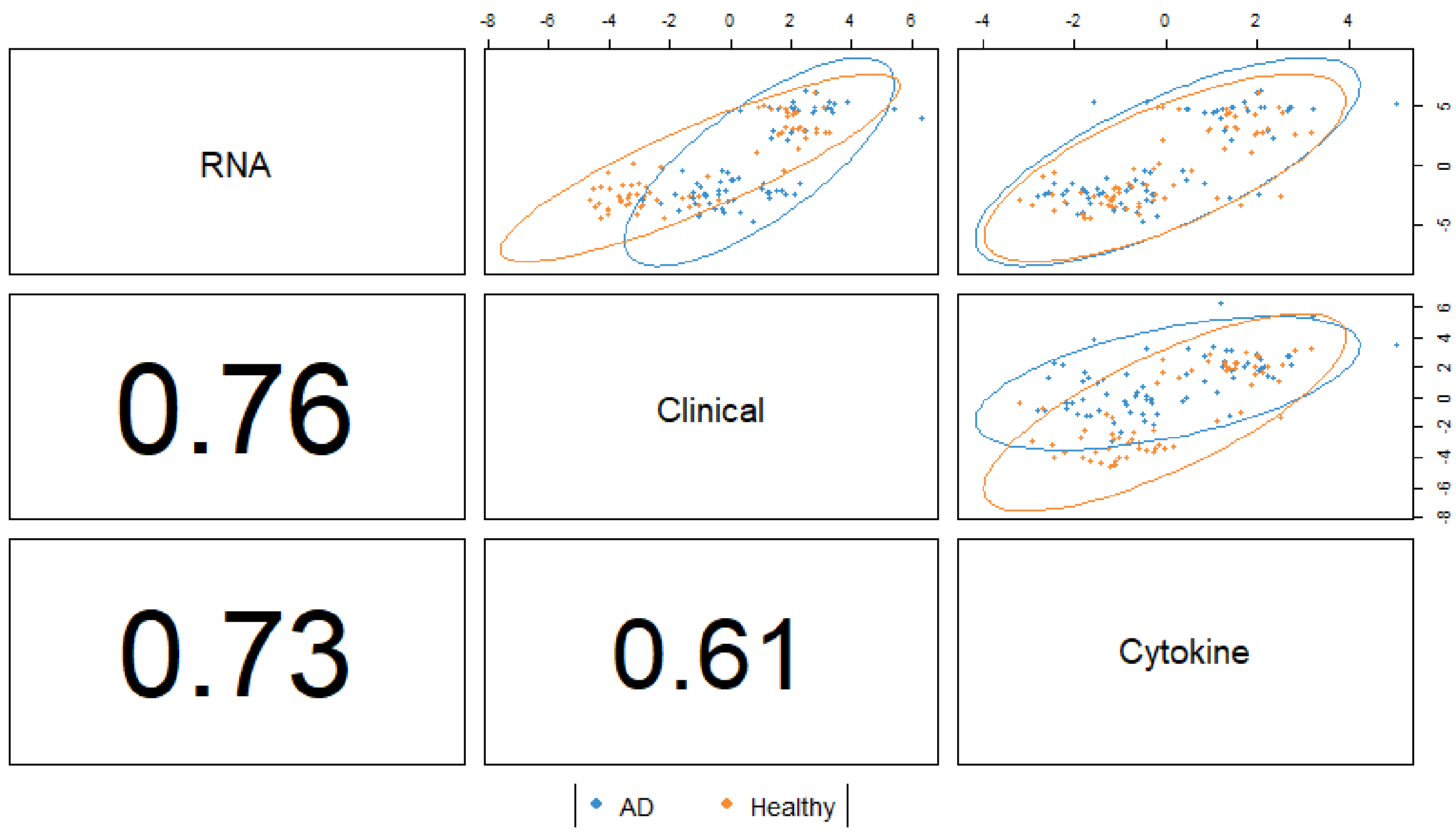

Fig. D Correlation scores between the RNA-Seq, the clinical questionnaire and antibody data, and the cytokine datasets (HC samples in orange, AD samples in blue). The clinical dataset corresponds to the combined questionnaire and antibody data.

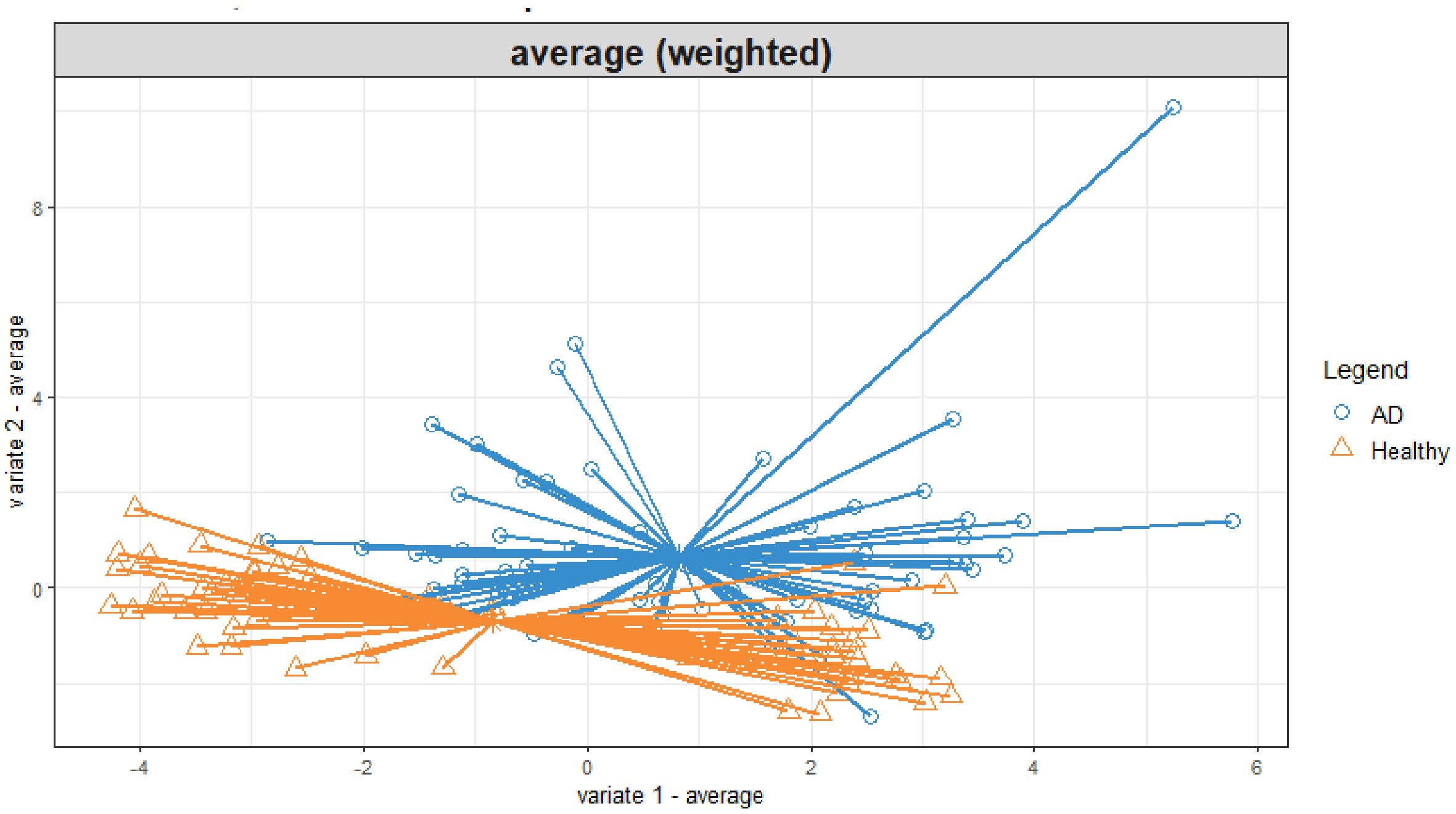

Fig. E Projections of samples in the first and second DIABLO components (HC samples in orange, AD samples in blue).

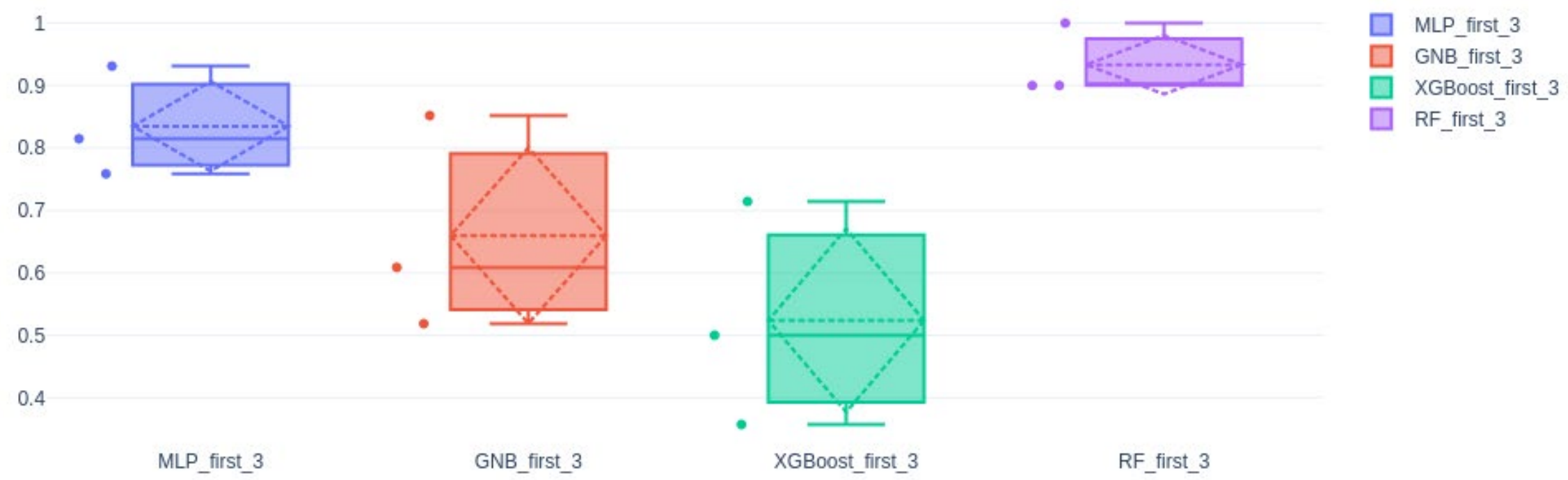

Fig. F Jaccard score for the overlap between the feature lists selected by the best 3 models of each ML algorithm.

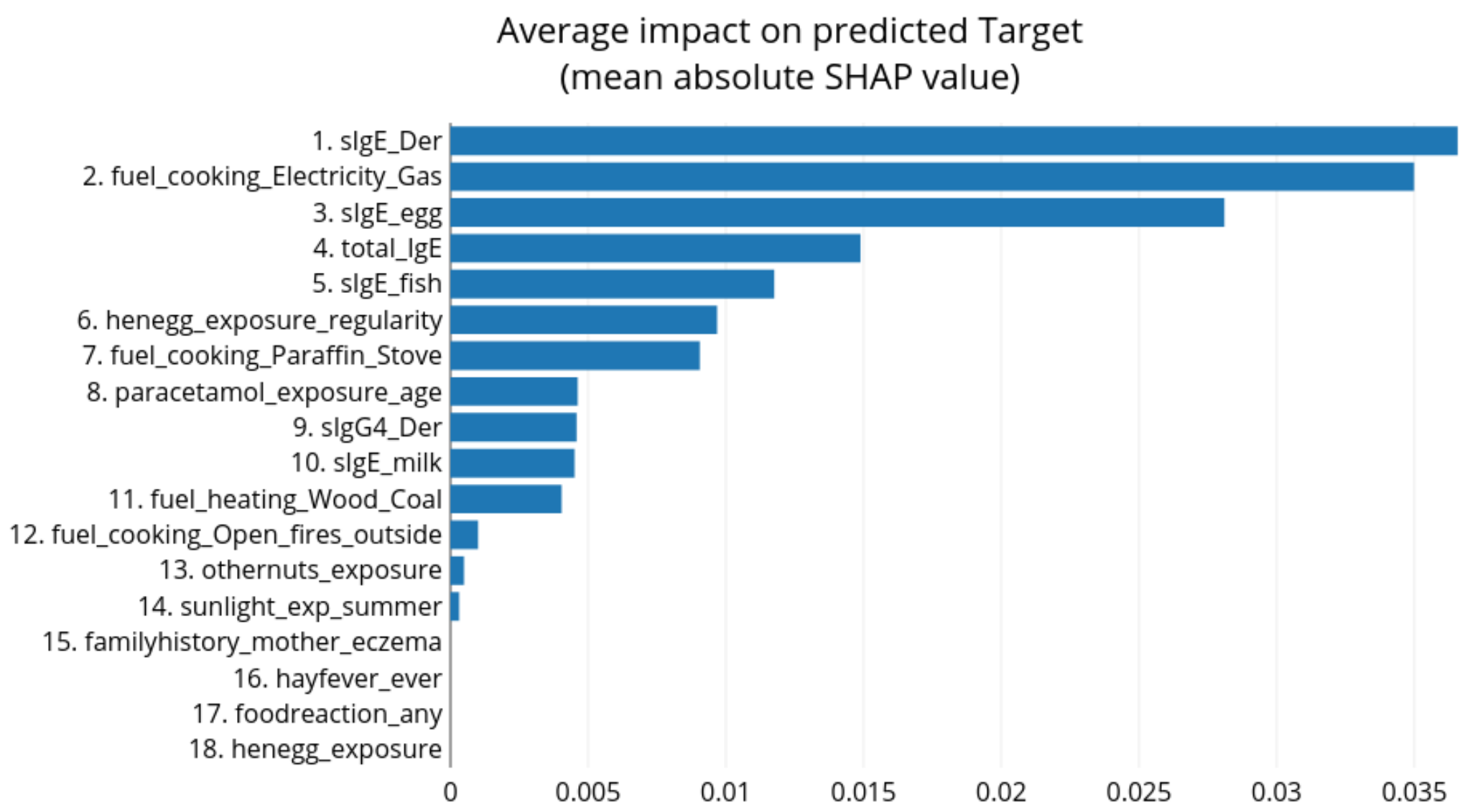

Fig. G Mean SHAP values for the 18 features selected by the Random Forest model M52, applied to the combined questionnaire and antibody data, showing their impact on the target prediction.

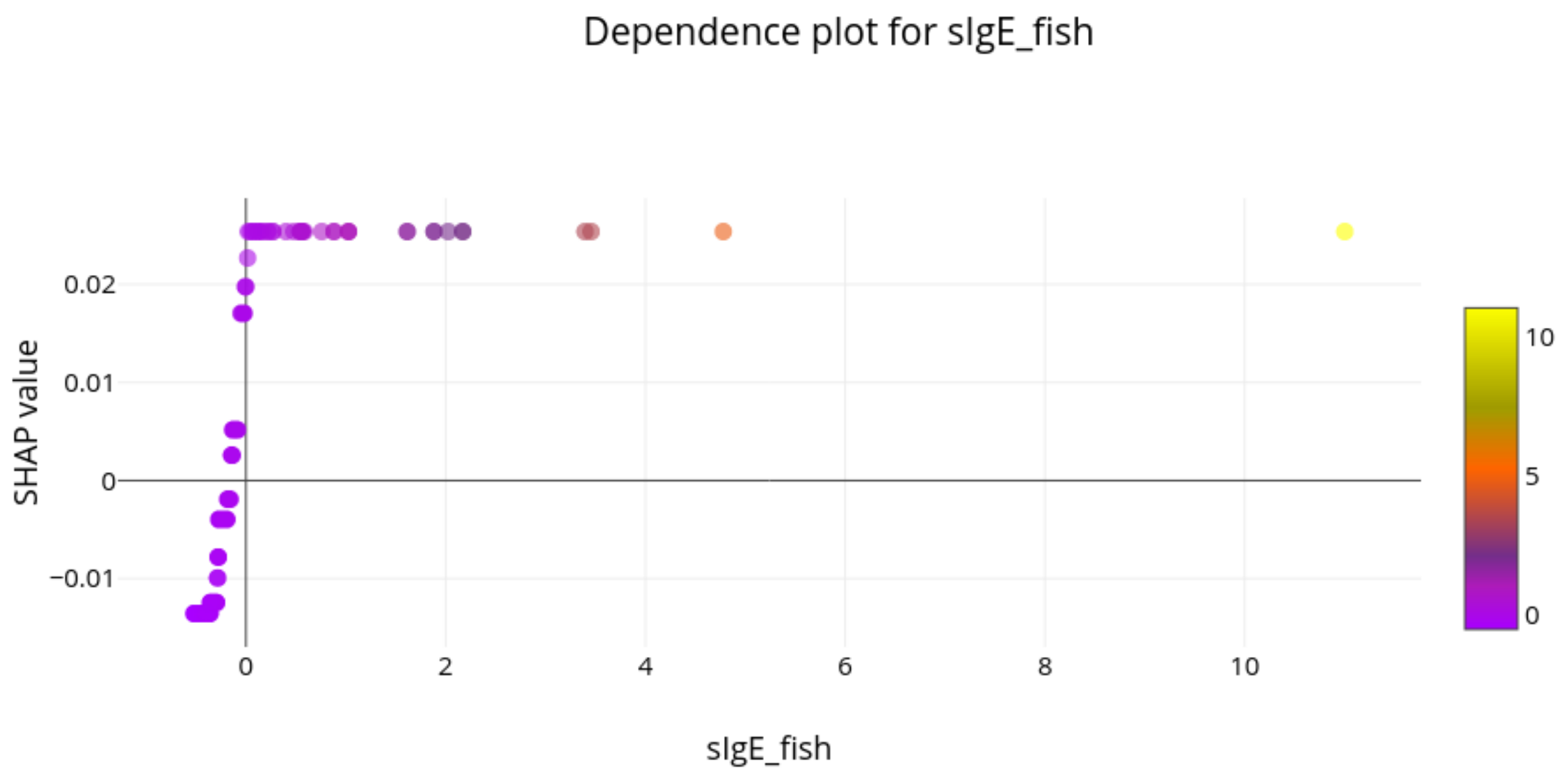

Fig. H SHAP dependence plot for the levels of specific IgE antibodies against hen egg.

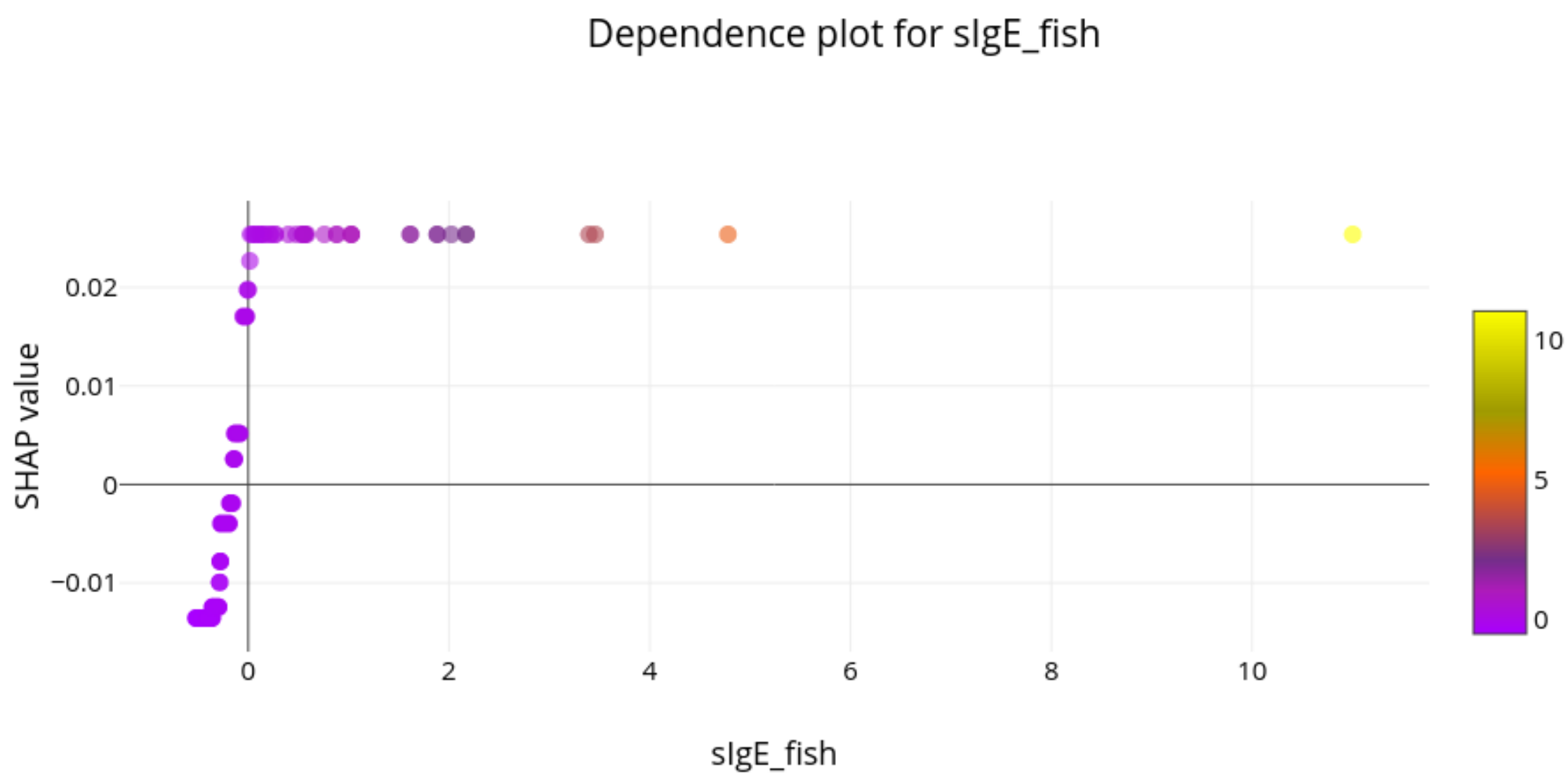

Fig. I SHAP dependence plot for the levels of specific IgE antibodies against fish.

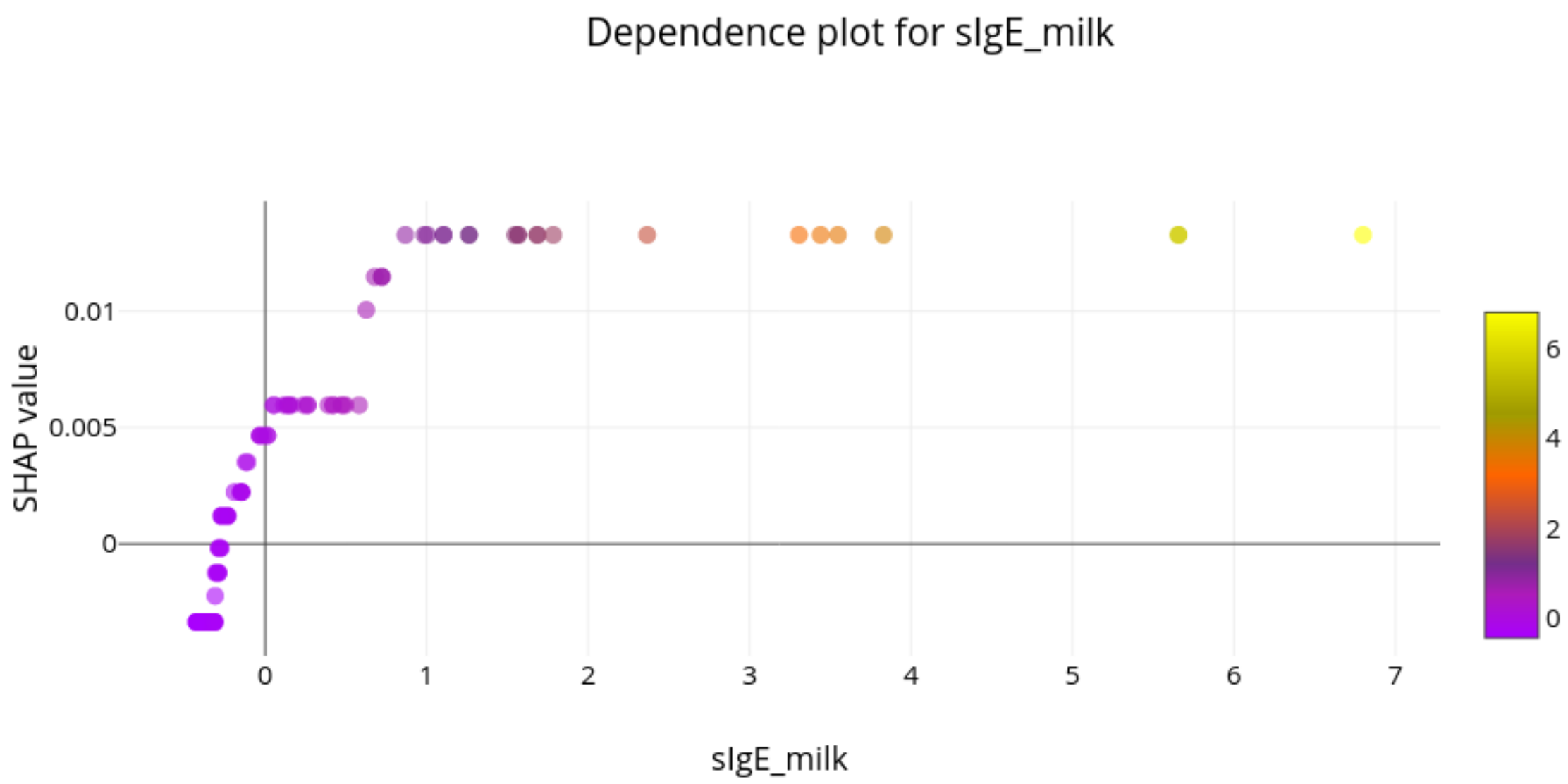

Fig. J SHAP dependence plot for the levels of specific IgE antibodies against milk.

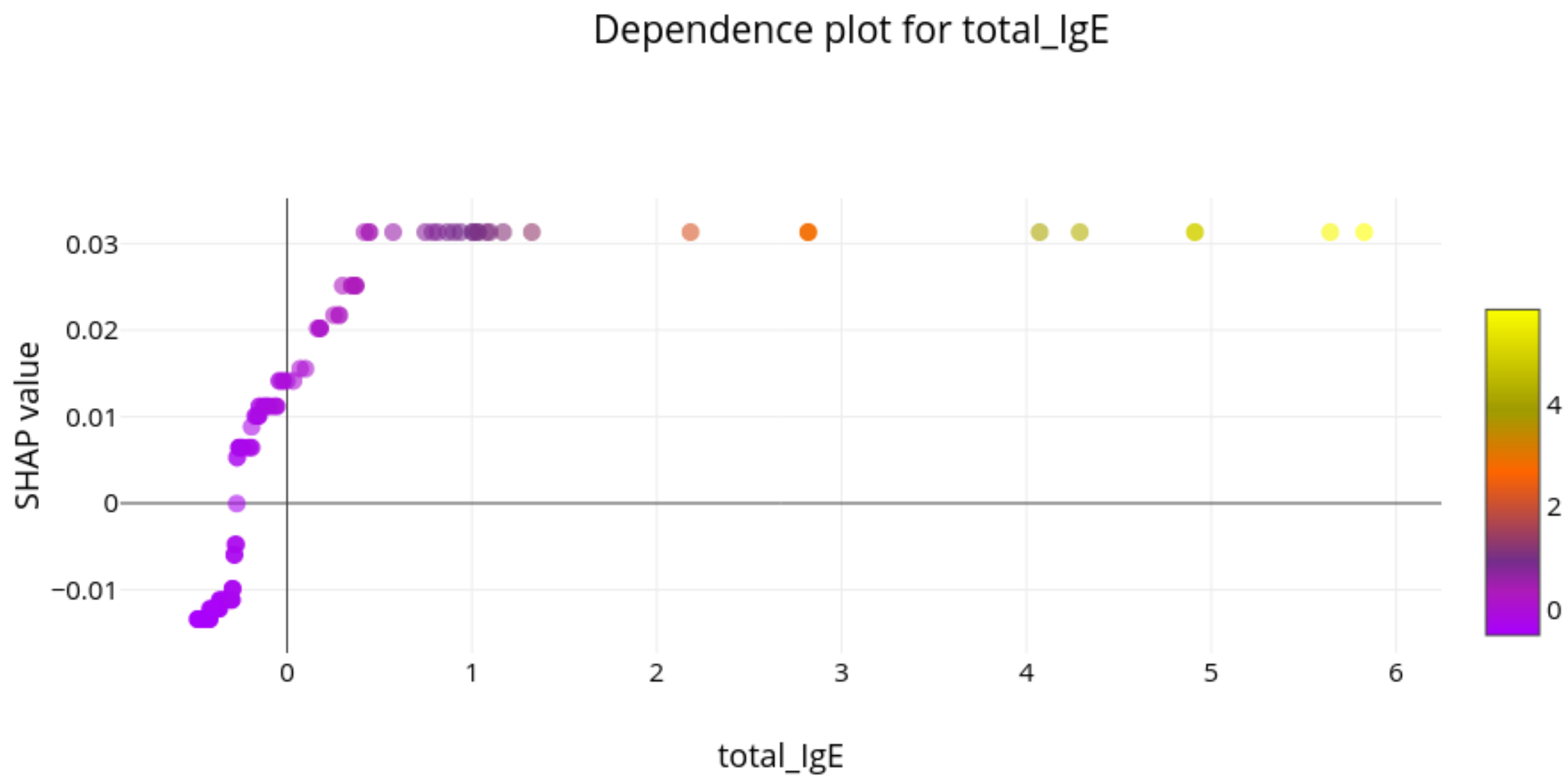

Fig. K SHAP dependence plot for the total levels of IgE antibodies.

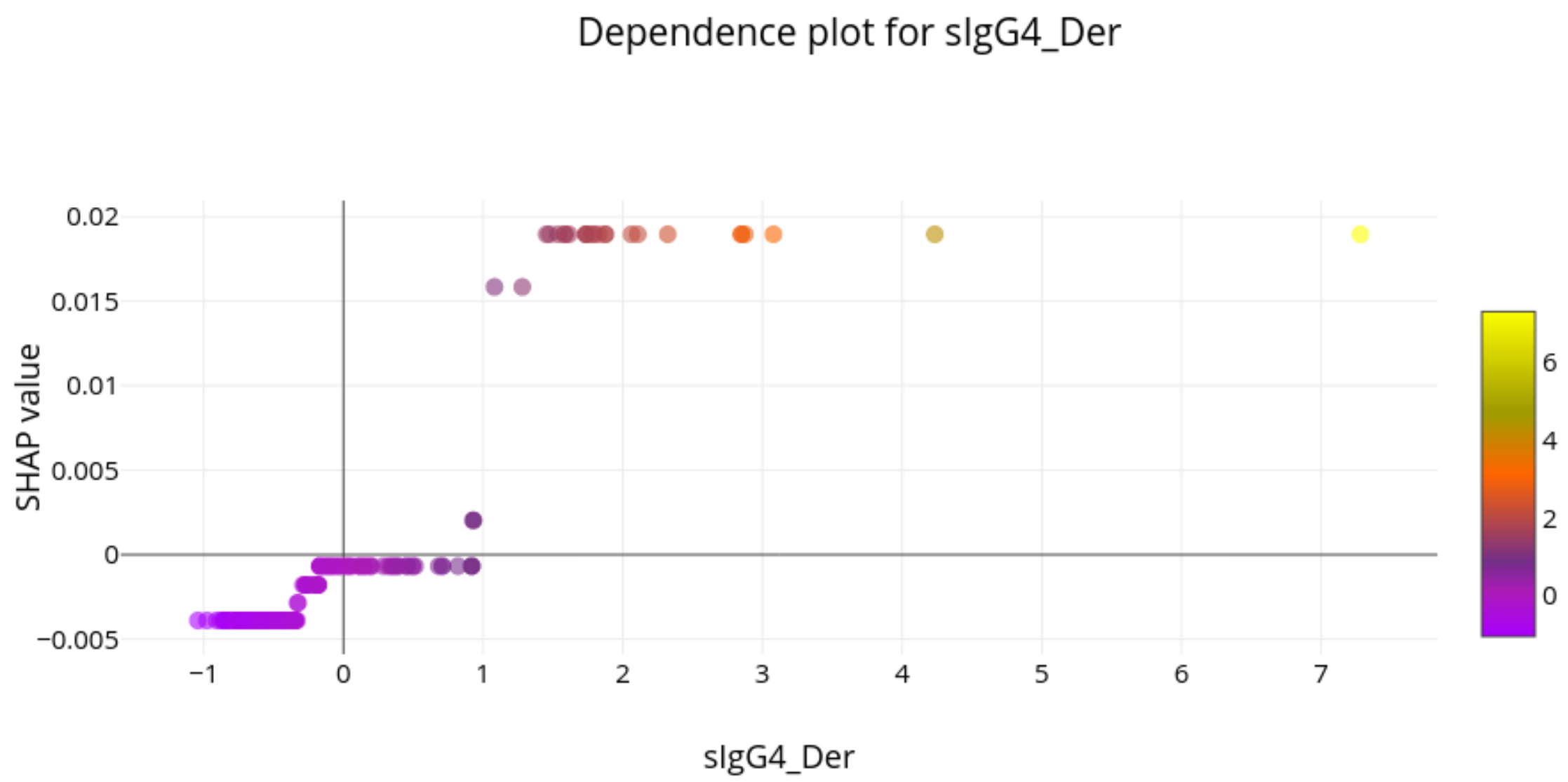

Fig. L SHAP dependence plot for the levels of specific IgG4 antibodies against house dust mites.

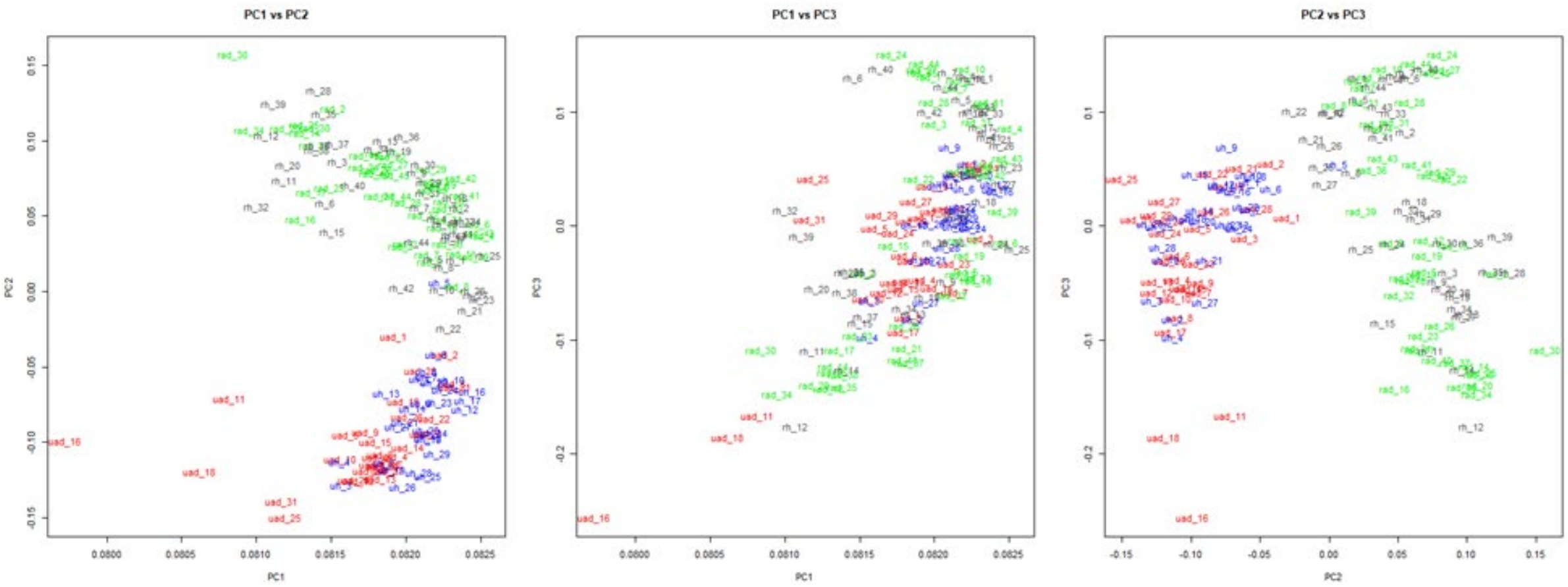

Fig. M PCA analysis of the RNA-Seq data with the Principal Components (PCs) 1-3 (green: rural – AD; grey: rural – HC; red: urban – AD; blue: urban – HC)

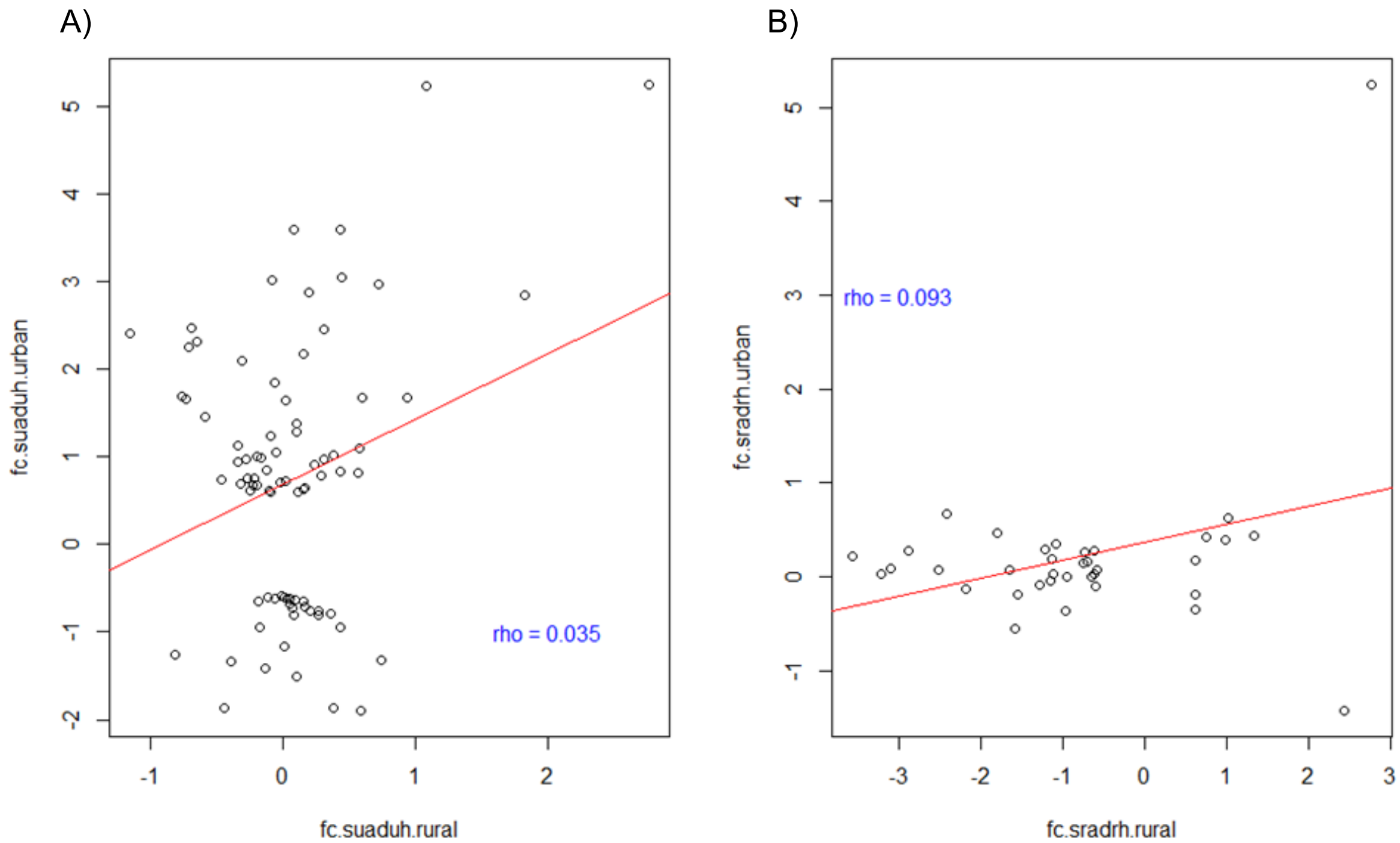

Fig. N Correlation of the fold changes of the DEGs identified in one comparison with the fold changes of these genes in the respective other comparison. A) Correlation of the fold changes of the genes identified with FDR < 0.05 and abs(fold change) > log2(1.5) in the comparison AD versus HC in urban samples. B) Correlation of the fold changes of the genes identified with FDR < 0.05 and abs(fold change) > log2(1.5) in the comparison AD versus HC in rural samples.

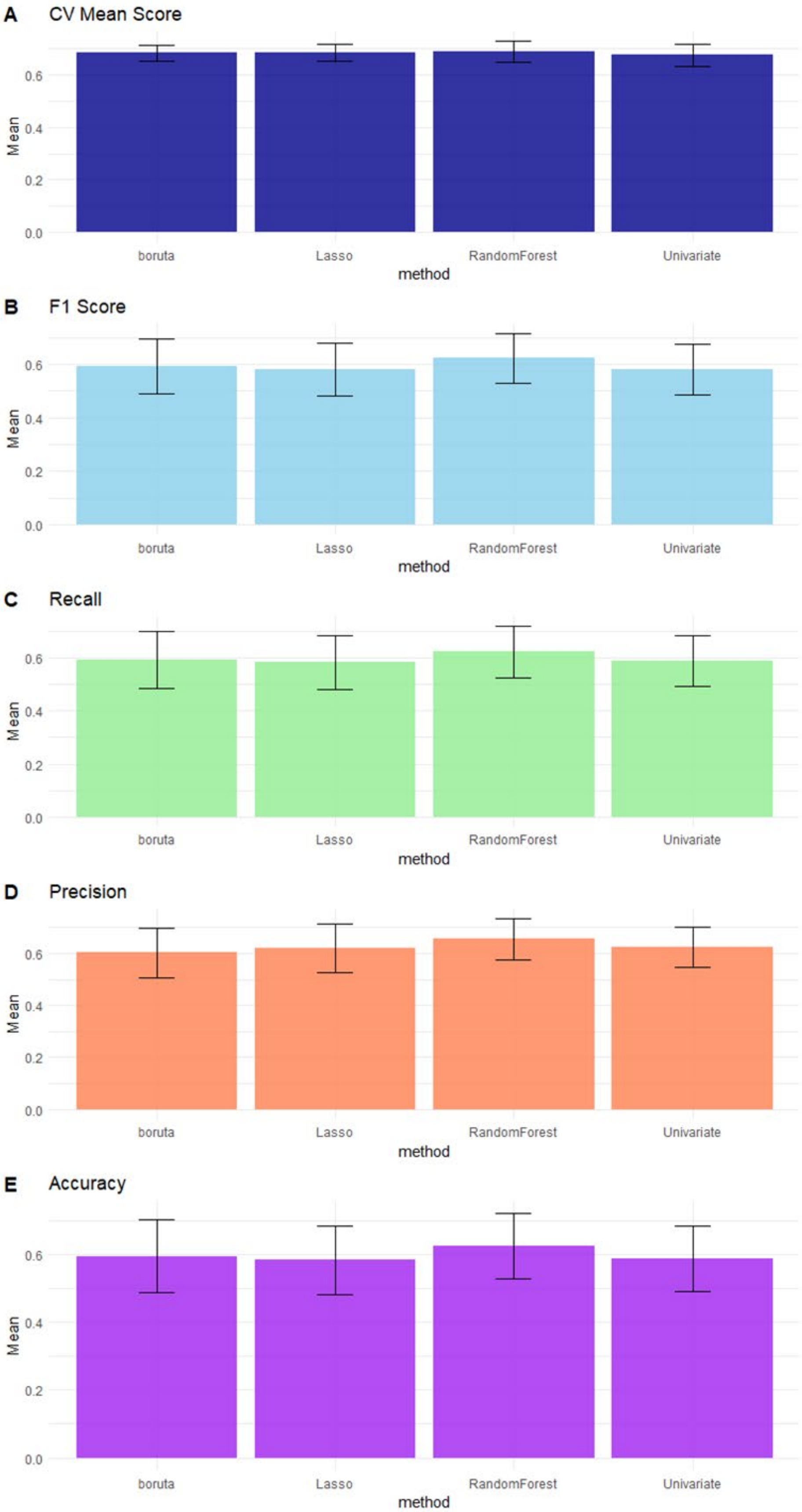

Fig. O Classification performance metrics of the four different ML methods.

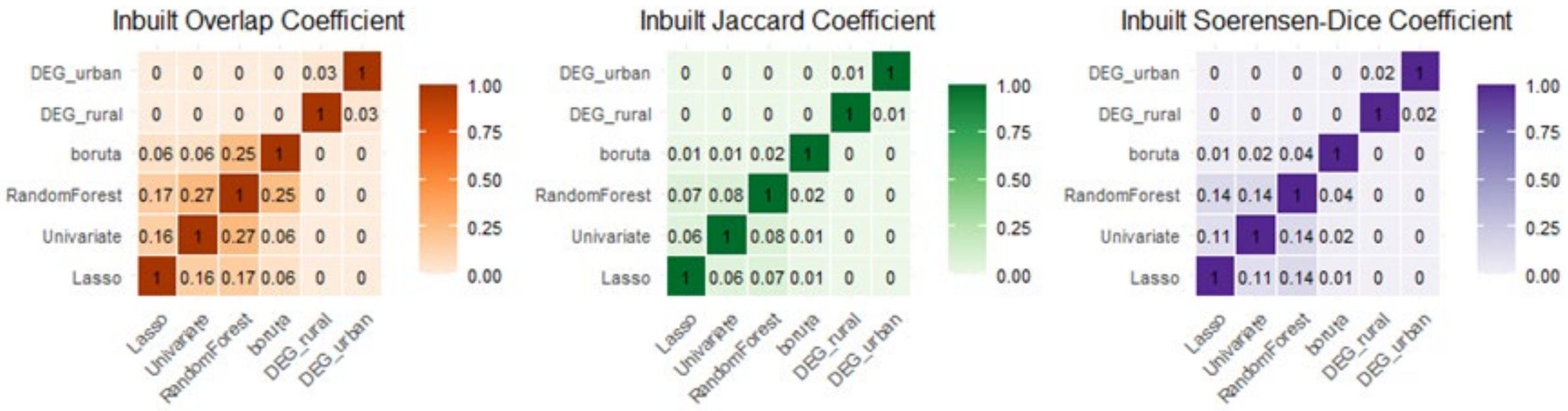

Fig. P Assessment of the overlap between selected feature lists from the four different ML Methods and the differential gene expression (DGE) lists using Overlap Coefficient, Jaccard Coefficient, and Sørensen-Dice Coefficient.

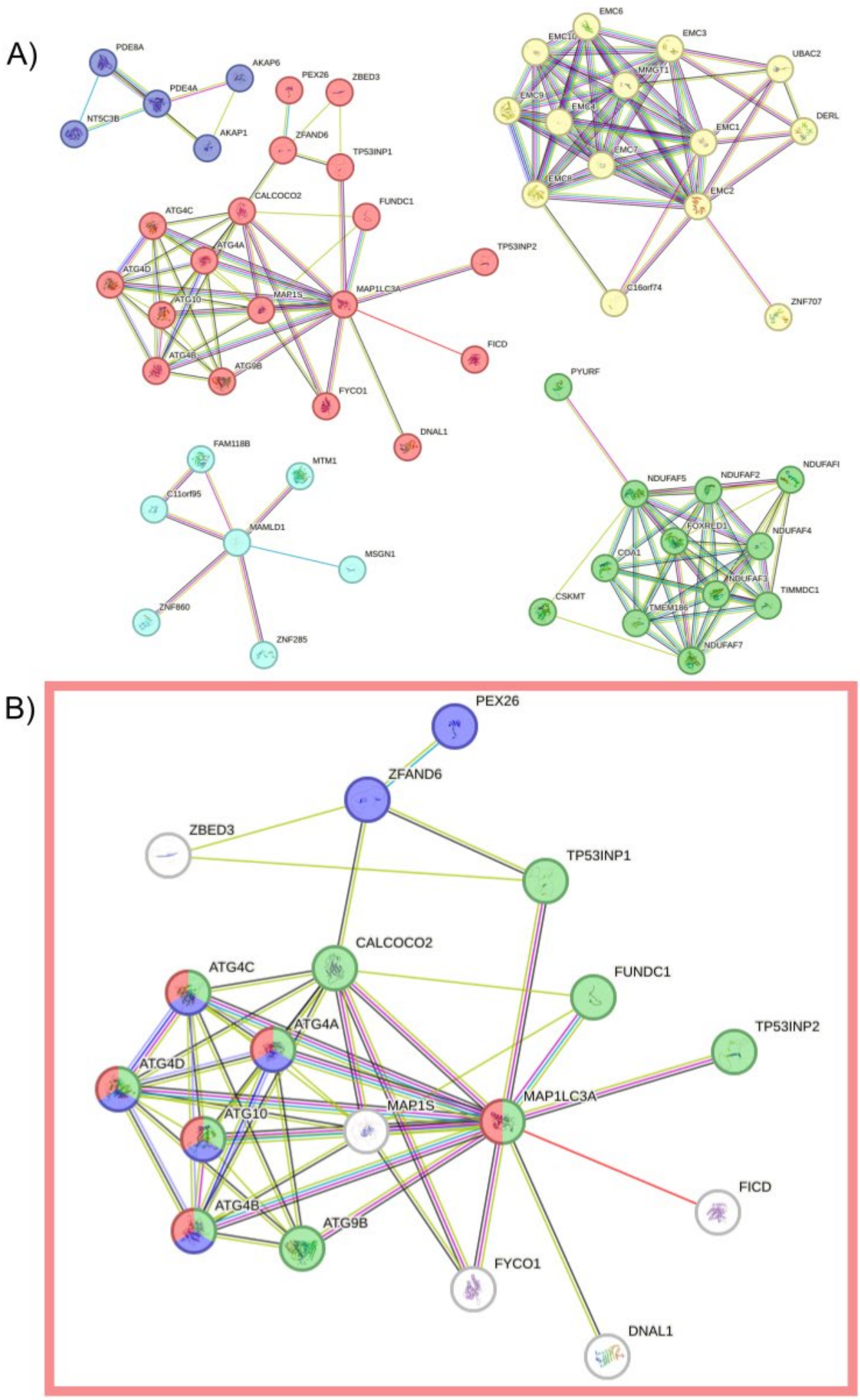

Fig. Q Genes in the violet DIABLO cluster subjected to network analysis and k-means subclustering in STRING, highlighting the red subcluster. A) The genes from the violet DIABLO cluster subjected to a network analysis in STRING (Szklarczyk et al., 2023), followed by a k-means clustering with the distinct subclusters coloured in red, violet, cyan, yellow, and green. B) The red subcluster from A) features ZFAND6 and MAP1LC3A. The nodes are coloured according to their UniProt annotated keywords *Autophagy* (green), *Protein transport* (blue), and *Ubl conjugation pathway* (red).

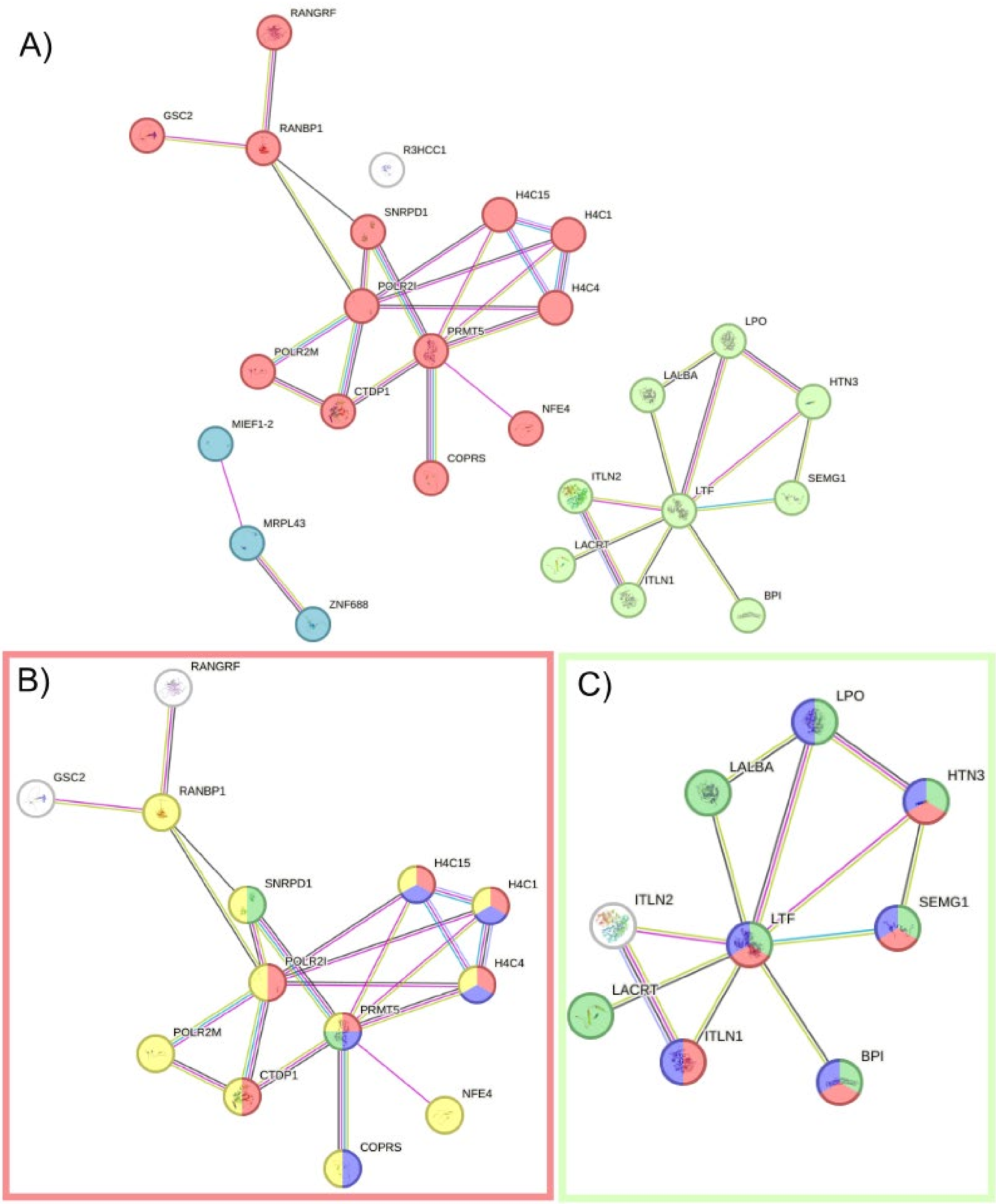

Fig. R Genes in the orange DIABLO cluster subjected to network analysis and k-means subclustering in STRING, highlighting the red and green subclusters. A) The genes from the orange DIABLO cluster subjected to a network analysis in STRING (Szklarczyk et al., 2023) with the interaction sources text mining, experiments, databases and co-expression with a maximum of 20 interactors in the 1st shell, followed by a k-means clustering with the distinct subclusters coloured in red, blue, and green. B) The nodes in the red subcluster from A) are coloured according to the functional enrichment terms: Subcellular localization: *Nucleus* (yellow); GO Biological Process: *Transcription, DNA-templated* (red) and *Chromatin organization* (purple); Local network cluster: *Methylosome* (green). C) The node in the green subcluster from A) are coloured according to the functional enrichment terms: GO Biological Process: *defense response to bacterium* (green); Reactome pathways: *Antimicrobial peptides* (red) and *Innate immune system* (purple).
