# Supplementary material for: Multimodal protective and susceptibility clusters in paediatric atopic dermatitis: A machine learning-based, data-driven observational study
Source: PLoS Med. 2026 Sep 8;23(9):e1004917. doi: 10.1371/journal.pmed.1004917 (PMC13552787; doi:10.1371/journal.pmed.1004917)
Supplement: Supplementary file 2 [file pmed.1004917.s002.pdf]

## APPENDIX IV: QUESTIONNAIRE

**Participant details**

| | | | |
|---|---|---|---|
| **Q1** | Participant ID | 0000/XX | |
| **Q2** | Enrolment date | DD/MM/YYYY | |
| **Q3** | Study site | 1 = Urban | 2= Rural |
| **Q4** | Date of birth | DD/MM/YYYY | |
| **Q5** | Age at enrolment | 00 months | |
| **Q6** | Sex | 1= Male | 2=Female |
| **Q7** | Weight | 00.0 kg | |
| **Q8** | Height/Length | 000 cm | |

**Q9 Immunisations**

| | | | Yes | No | | | | Yes | No |
|---|---|---|---|---|---|---|---|---|---|
| Birth | **9.1** | BCG | | | 14 weeks | **9.10** | RV(2) | | |
| | **9.2** | OPV (0) | | | | **9.11** | DTaPIPV/HiB(3) | | |
| 6 weeks | **9.3** | OPV (1) | | | | **9.12** | HepB(3) | | |
| | **9.4** | RV (1) | | | | **9.13** | PCV7 or 13 (2) | | |
| | **9.5** | DTaPIPV/HiB (1) | | | 9 months | **9.14** | Measles vaccine | | |
| | **9.6** | HepB (1) | | | | **9.15** | PCV 7 or 13 (3) | | |
| | **9.7** | PCV7 or 13(1) | | | 18 months | **9.16** | DTaPIPV/HiB (4) | | |
| 10 weeks | **9.8** | DTaPIPV/HiB (2) | | | | **9.17** | Measles Vaccine | | |
| | **9.9** | HepB (2) | | | | | | | |

**Q10 Vaccination Status**

| | | | |
|---|---|---|---|
| 1 = Complete | | 2 = Incomplete | |

**Q11 Paracetamol Exposure**

| | | | | | |
|---|---|---|---|---|---|
| **Q11.1** | Did your child have paracetemol or medicines containing paracetamol in the first year of life? ( Panado, Calpol, Paramed) | | | 0 = No | 1 = Yes |
| **Q11.2** | If Yes, How old was your child when they first had paracetamol? | | | Months | |
| **Q11.3** | If Yes, How often did your child have paracetamol on the first year of life? | | 1 =<br><br>1-10 days | 2 =<br><br>10-20 days | 3 =<br><br>More than 20 days |

**Q12 Childhood infections**

| Has your child had any of the following childhood infections? | | | | | | | | | |
|---|---|---|---|---|---|---|---|---|---|
| | | **0 = No** | **1 = Yes** | **999 = don' t know** | | | **0 = No** | **1 = Yes** | **999 = don't know** |
| **12.1** | Measles | | | | **12.5** | Glandular Fever | | | |
| **12.2** | Mumps | | | | **12.6** | Tuberculosis | | | |
| **12.3** | Rubella | | | | **12.7** | Hepatitis | | | |
| **12.4** | Chickenpox | | | | **12.8** | Other (please specify) | | | |

**Q13 Antibiotic and anti-helminthic exposure**

| | | | | | |
|---|---|---|---|---|---|
| **13.1** | Did your child have any antibiotics in the first year of life? | **0 = no** | **1 = Yes** | **999** = Don't know | |
| **13.2** | If Yes, how old were they with the first course?? | | | Months | |
| **13.3** | How many courses did your child have in the first year of life? | **1** =<br>None | **2** =<br>1 to 2 Courses | **3** =<br>3 to 5 courses | **4** =<br>> 5 courses |
| **13.4** | Did your child have any antibiotics in the last 2 months? | **0 = No** | **1 = Yes** | **999** = Don't know | |

| | | | | |
|---|---|---|---|---|
| | | | | |

| | | | | |
|---|---|---|---|---|
| **13.5** | Has your child ever had medicines for worms? | 0= No | 1 = Yes | 999=Don't Know |
| **13.6** | If Yes, at what age were they dewormed? | | | months |
| **13.7** | If Yes, at what age did they last take anti worm medicines? | | | months |
| **13.8** | Has your child had regular (yearly) medicine for worms? | 0 = No | 1 = Yes | 999 = Don't know |

**Q14 Probiotic Exposure and *Amasi* Exposure (CHILD)**

| | | | | |
|---|---|---|---|---|
| **14.1** | Did this **child** have probiotics in food or supplements in the first year of life? | **0 = No** | **1 = Yes** | **999** = Don't know |
| **14.2** | **If Yes**, how old was this child when they first had probiotics? | | | Months |
| **14.3** | **If Yes**, How often did this child have probiotics in the first year of life? | **1 =** 1-10 days | **2 =** 10-20 days | **3 =** >20 days |
| **14.4** | **If Yes**, which probiotic product were they given? | | | |

| | | | | |
|---|---|---|---|---|
| **14.3** | Has your **child** ever have **amasi** ? | **0 = No** | **1 = Yes** | **999** = Don't know |
| **14.4** | **If Yes**, how old was this child when they first had **amasi**? | | | Months |
| **14.5** | **If Yes**, how often does your child take amasi? | **1 =** Less than once a month | **2 =** 1-4 times per month | **3 =** more than 4 times per month |

**Probiotic and *Amasi* exposure (MOTHER)**

| | | | | |
|---|---|---|---|---|
| **14.6** | Did this child's mother have probiotics during pregnancy? | **0** = No | **1** = Yes | **999** =Don't know |
| **14.7** | If Yes, How often did she use probiotics? | **1** = 1-10 days | **2** = 10-20 d ays | **3** = >20 days |
| **14.8** | Which yes, which products did she use? | | | |
| | | | | |

| | | | | |
|---|---|---|---|---|
| **14.9** | Did this child's mother regularly have amasi during pregnancy?<br>(more than once a month) | **0** = No | **1** =Yes | **999** = Don't know |

**Q15 Delivery mode**

| | | | | |
|---|---|---|---|---|
| **Q15** | How was this child born? | **1** = Normal vaginal delivery | **2** = Caesarean section | **999** = Don't Know |

**Q16 Sunlight Exposure**

| How much time does your child spend outdoors on an average day?<br>*(add crèche daily average X5, to weekend total hours and divide by 7. Report to nearest 0.1)* | |
|---|---|
| **Q16.1 Winter** | **Q16.2 Summer** |
| 00.0 hours | 00.0 hours |
| **999 =** Don't Know | **999 =** Don't Know |

**Q17 Peanut exposure in pregnancy**

| | | | | |
|---|---|---|---|---|
| **17.1** | Did this child's mother eat peanuts regularly (every week) during pregnancy? | **0** = No | **1** = Yes | **999** = Don't know |
| **17.2** | Did this child's mother completely avoid peanuts during pregnancy? | **0** = No | **1** = Yes | **999** = Don't know |

**Q18 Breastfeeding information**

| | | | | |
|---|---|---|---|---|
| **Q18.1** | Was this child ever breastfed? | **0** = No | **1** = Yes | **999** = Don't know |
| **Q18.2** | If Yes, up to what age was this child exclusively breastfed<br>(i.e. no milk or other fluids via bottle nor given any solids) | | Months | **999** = Don't know |
| **Q18.3** | At what age did you completely stop breastfeeding this child? | | Months | **999** = Don't know |
| | | | Months | |

| | | | | |
|---|---|---|---|---|
| **Q18.4** | At what age did you first introduce any other milk (other than breast milk) | | | **999** = Don't know |

**Q19 Weaning foods**

| | | | | |
|---|---|---|---|---|
| **Q19.1** | When did you first introduce solids into your child's diet? | | | months |
| **Q19.2** | Which three types of foods did you introduce first**?** *(see coding chart for food categories)* | Free Text | Free text | Free text |

**Q20 Exposure to Unpasteurised milk**

| | | | | |
|---|---|---|---|---|
| **Q20** | Has this child ever had non-pasteurised (Fresh farm) milk? | **0** = No | **1** = Yes | **999** = Don't know |

**Q21 Food exposure**

| **Foods Eaten** | | **Ever Eaten** | | | **Age first eaten** | | | | **Still eating regularly** *(once a month or more)* | | |
|---|---|---|---|---|---|---|---|---|---|---|---|
| **21.1** | **Peanut**<br>e.g. peanut butter, peanut oil, peanuts in cookies or chocolates | **21.1.1** | 0 = No | 1 = Yes | **21.1.2** | | months | 999 = don't know | **21.1.3** | 0 = No | 1 = Yes<br>**If yes please continue with 21.1.4** |

| If yes to 21.1.4<br><br>**Please try an remember how many times your child ate the following peanut containing foods in the last day and *week*** | **Type of peanuts consumed** | **Number of times per day** | **Number of times per week** | **Usual amount** | | | **Total amount in grams** |
|---|---|---|---|---|---|---|---|
| | Peanut butter on bread | | | | Thin slices | **21.1.4** | |
| | | | | | Thick slices | **21.1.5** | |
| | Peanut butter in porridge | | | | Teaspoons | **21.1.6** | |
| | Raw/boiled peanuts | | | | Handfuls | **21.1.7** | |
| | Roasted peanuts | | | | Handfuls | **21.1.8** | |
| | Peanuts in chocolate or biscuits | | | | | **21.1.9** | |
| | **Total amount of peanut consumed in the last week** | | | | | **21.1.10** | |

| | | | | | | | | | | | |
|---|---|---|---|---|---|---|---|---|---|---|---|
| **21.2** | **Other nuts** e.g. Cashew, hazelnut, brazil nut, almonds, walnuts, pistachio, macadamia in chocolate and cookies or cakes and breads | **21.2.1** | 0 = No | 1 = Yes | **21.2.2** | | months | 999 = don't know | **21.2.3** | 0 = No | 1 = Yes |
| **21.3** | **Cow's milk products** e.g. fresh milk, cheese, yoghurt, cream | **21.3.1** | 0 = No | 1 = Yes | **21.3.2** | | months | 999 = don't know | **21.3.3** | 0 = No | 1 = Yes |
| **21.4** | **Cow's milk formula** e.g. Nan, Lactogen, Novolac, S26, Infacare, Pelargon | **21.4.1** | 0 = No | 1 = Yes | **21.4.2** | | Months | 999 = don't know | **21.4.3** | 0 = No | 1 = Yes |
| **21.5** | **Soya products** e.g. soya mince, soya sauce, tofu, baby foods, soya in bread | **21.5.1** | 0 = No | 1 = Yes | **21.5.2** | | Months | 999 = don't know | **21.5.3** | 0 = No | 1 = Yes |
| **21.6** | **Soya milk products** e.g. Infasoy, Isomil, Infacare | **21.6.1** | 0 = No | 1 = Yes | **21.6.2** | | Months | 999 = don't know | **21.6.3** | 0 = No | 1 = Yes |

| | | | | | | | | | | | |
|---|---|---|---|---|---|---|---|---|---|---|---|
| **21.7** | **Hen's egg**<br>e.g. boiled, scrambles eggs, omelettes, quiches(souttert), cakes, cookies, rusks | **21.7 .1** | 0 = No | 1 = Yes | **21.7 .2** | | Mont hs | 999 = don't know | **21.7 .3** | 0 = No | 1 = Yes |
| **21.8** | **Wheat**<br>e.g. cereal (Wheetabix, All Bran, Tasty Wheat), bread or pasta, cakes, rusks, couscous, semolina | **21.8 .1** | 0 = No | 1 = Yes | **21.8 .2** | | Mont hs | 999 = don't know | **21.8 .3** | 0 = No | 1 = Yes |
| **21.9** | **Fish (excluding shellfish)**<br>Hake, snoek, sardines,pilchards, tuna, kingklip, salmon etc and fish products: Fish paste(Redro), Worcester sauce, fish sauce | **21.9 .1** | 0 = No | 1 = Yes | **21.9 .2** | | mont hs | 999 = don't know | **21.9 .3** | 0 = No | 1 = Yes |

**Q22 Food Reactions**

| **22.1 PEANUTS** | | | | |
|---|---|---|---|---|
| **22.1.1** | Has your child ever had any of these reactions below to peanuts or food containing peanuts | | **0 = No** | **1 = Ye** |
| **22.1.2** | If yes, which of the following reactions has this child had? (you can circle more than one) | | | |
| | **1**= none | **6**= tight throat | **11**=shock/low blood pressure | |
| | **2**= itchy Rash | **7**= wheeze | **12**= collapse/loss of consciousness | |
| | **3**=swelling(face/lips/eyes) | **8**= vomiting | **13** = worsening of eczema | |
| | **4**= flushing | **9**= diarrhoea | | |
| | **5**= itchy mouth/throat | **10**= blue lips | | |
| **22.1.3** | If Yes, how old was this child when he/she had the first reaction? | | | Months |
| **22.1.4** | If yes, how long after the eating the food did the reaction occur? | **1 =**<br>< 1 hour | **2 =**<br>1-24 hours | **3 =**<br>>24 hours |
| **22.1.5** | If yes, was this reaction confirmed by a doctor? | | **0 = No** | **1 = Yes** |

| 22.2 OTHER NUTS | | | | | |
|---|---|---|---|---|---|
| **22.2.1** | Has your child ever had ny of these reactions below to other nuts or food containing nuts other than peanuts | | **0 = No** | **1 = Yes** | |
| **22.2.2** | If yes, which of the following reactions has this child had? (you can circle more than one) | | | | |
| | **1** = none | **6** = tight throat | **11** =shock/low blood pressure | | |
| | **2** = itchy Rash | **7** = wheeze | **12** = collapse/loss of consciousness | | |
| | **3** =swelling(face/lips/eyes) | **8** = vomiting | **13** = worsening of eczema | | |
| | **4** = flushing | **9** = diarrhoea | | | |
| | **5** = itchy mouth/throat | **10** = blue lips | | | |
| **22.2.3** | If Yes, how old was this child when he/she had the first reaction? | | | Months | |
| **22.2.4** | If yes, how long after the eating the food did the reaction occur? | | **1 =** < 1 hour | **2 =** 1-24 hours | **3 =** >24 hours |
| **22.2.5** | If yes, was this reaction confirmed by a doctor? | | **0 = No** | 1 = Yes | |

| 22.3 COW'S MILK | | | | | |
|---|---|---|---|---|---|
| **22.3.1** | Has your child ever had any of these reactions below to cow's milk or food containing cow's milk | | **0 = No** | **1 = Yes** | |
| **22.3.2** | If yes, which of the following reactions has this child had? (you can circle more than one) | | | | |
| | **1** = none | **6** = tight throat | **11** = shock/low blood pressure | | |
| | **2** = itchy Rash | **7** = wheeze | **12** = collapse/loss of consciousness | | |
| | **3** =swelling(face/lips/eyes) | **8** = vomiting | **13** = worsening of eczema | | |
| | **4** = flushing | **9** = diarrhoea | | | |
| | **5** = itchy mouth/throat | 10 = blue lips | | | |
| **22.3.3** | If Yes, how old was this child when he/she had the first reaction? | | | Months | |
| **22.3.4** | If yes, how long after the eating the food did the reaction occur? | | **1 =** < 1 hour | **2 =** 1-24 hours | **3 =** >24 hours |
| **22.3.5** | If yes, was this reaction confirmed by a doctor? | | **0 = No** | **1 = Yes** | |

| 22.4 SOYA | | | |
|---|---|---|---|
| **22.4.1** | Has your child ever had any of these reactions below to soya or food containing soya | **0 = No** | **1 = Yes** |
| **22.4.2** | If yes, which of the following reactions has this child had? (you can circle more than | | |

| | | | |
|---|---|---|---|
| | one) | | |
| | 1 = none | 6= tight throat | 11=shock/low blood pressure |
| | 2 = itchy Rash | 7= wheeze | 12= collapse/loss of consciousness |
| | 3 =swelling(face/lips/eyes) | 8= vomiting | 13 = worsening of eczema |
| | 4 = flushing | 9= diarrhoea | |
| | 5 = itchy mouth/throat | 10= blue lips | |

| | | | | |
|---|---|---|---|---|
| **22.4.3** | If Yes, how old was this child when he/she had the first reaction? | | Months | |
| **22.4.4** | If yes, how long after the eating the food did the reaction occur? | **1 =** < 1 hour | **2 =** 1-24 hours | **3 =** >24 hours |
| **22.4.5** | If yes, was this reaction confirmed by a doctor? | **0 = No** | **1 = Yes** | |

| | | | | |
|---|---|---|---|---|
| **22.5 HEN'S EGG** | | | | |
| **22.5.1** | Has your child ever had any of these reactions below to hen's egg or food containing egg | **0 = No** | **1 = Yes** | |
| **22.5.2** | If yes, which of the following reactions has this child had? (you can circle more than one) | | | |
| | 1 = none | 6 = tight throat | 11 =shock/low blood pressure | |
| | 2 = itchy Rash | 7 = wheeze | 12 = collapse/loss of consciousness | |
| | 3 =swelling(face/lips/eyes) | 8 = vomiting | 13 = worsening of eczema | |
| | 4 = flushing | 9 = diarrhoea | | |
| | 5 = itchy mouth/throat | 10= blue lips | | |
| **22.5.3** | If Yes, how old was this child when he/she had the first reaction? | | months | |
| **22.5.4** | If yes, how long after the eating the food did the reaction occur? | 1= < 1 hour | **2=** 1-24 hours | **3=** >24 hours |
| **22.5.5** | If yes, was this reaction confirmed by a doctor? | **0 = No** | **1 = Yes** | |

| | | | |
|---|---|---|---|
| **22.6 WHEAT** | | | |
| **22.6.1** | Has your child ever had any of these reactions below to wheat or food containing wheat | **0 = No** | 1 = Yes |
| **22.6.2** | If yes, which of the following reactions has this child had? (you can circle more than one) | | |
| | 1= none | 6= tight throat | 11=shock/low blood pressure |
| | 2= itchy Rash | 7= wheeze | 12= collapse/loss of consciousness |
| | 3=swelling(face/lips/eyes) | 8= vomiting | 13 = worsening of eczema |
| | 4= flushing | 9= diarrhoea | |

| | | | | | |
|---|---|---|---|---|---|
| | 5= itchy mouth/throat | 10= blue lips | | | |
| **22.6.3** | If Yes, how old was this child when he/she had the first reaction? | | | Months | |
| **22.6.4** | If yes, how long after the eating the food did the reaction occur? | | **1=** < 1 hour | **2=** 1-24 hours | **3=** >24 hours |
| **22.6.5** | If yes, was this reaction confirmed by a doctor? | | **0 = No** | **1 = Yes** | |

| | | | |
|---|---|---|---|
| **22.7 FISH (excluding shellfish)** | | | |
| **22.7.1** | Has your child ever had any of these reactions below to fish or food containing fish | **0 = No** | **1 = Yes** |
| **22.7.2** | If yes, which of the following reactions has this child had? (you can circle more than one) | | |
| | 1 = none | 6 = tight throat | 11 =shock/low blood pressure |
| | 2 = itchy Rash | 7 = wheeze | 12 = collapse/loss of consciousness |
| | 3 =swelling(face/lips/eyes) | 8 = vomiting | 13 = worsening of eczema |
| | 4 = flushing | 9 = diarrhoea | |
| | 5 = itchy mouth/throat | 10 = blue lips | |
| **22.7.3** | If Yes, how old was this child when he/she had the first reaction? | | Months |
| **22.7.4** | If yes, how long after the eating the food did the reaction occur? | **1=** < 1 hour | **2=** 1-24 hours / **3=** >24 hours |
| **22.7.5** | If yes, was this reaction confirmed by a doctor? | **0 = No** | **1 = Yes** |

**Q23 Asthma**

| | | | | |
|---|---|---|---|---|
| **Q23.1** | Has your child ever symptoms of asthma without having a cold or chest infection? (e.g. wheeze, persistent cough at night or when exercising, shortness of breath) | **0 = No** | **1 = Yes** | |
| **Q23.2** | If Yes, how old was he/she? | | Months | |
| **Q23.3** | If yes, who diagnosed the asthma? | **1** = Self | **2** = Nurse | **3** = Doctor |

**Q24 Hay fever**

| | | | | |
|---|---|---|---|---|
| **Q24.1** | Has your child ever had symptoms of hay fever (e.g. itchy runny eyes, itchy runny nose, blocked nose, frequent sneezing) without having a “cold” or upper respiratory tract infection? | **0 = No** | | **1 = Yes** |
| **Q24.2** | If Yes, how old was he/she? | | | Months |
| **Q24.2** | If yes, who diagnosed the hay fever? | **1** = Self | **2** = Nurse | **3** = Doctor |

**Q25 Eczema**

| | | | | |
|---|---|---|---|---|
| **Q25.1** | Has your child ever had symptoms of eczema (e.g. an itchy rash especially in the folds of the elbows, behinds the knees, in front of the ankles, under the buttocks or around the neck, ears or eyes?) | **0 = No** | | **1 = Yes** |
| **Q25.2** | If yes, how old was your child then? | | | Months |
| **Q25.3** | If yes, who diagnosed the eczema? | **1** = Self | **2** = Nurse | **3** = Doctor |

**Q26 Medication use**

| | | | | | | | | |
|---|---|---|---|---|---|---|---|---|
| **Q26.1** | Is your child on any of the following medication? (see NAEP Chart) | | | | | | | |
| **26.1.1** | | Relievers (blue) e.g. Asthavent | 0 = No | 1 = Yes | **25.1.5** | Nasal corticosteroid spray e.g. Beclate | 0 = No | 1 = Yes |
| **26.1.2** | INHALERS | Controllers (brown/cream) e.g. Budeflam | 0 = No | 1 = Yes | **25.1.6** | Antihistamines **(If yes, please complete Q26.4 below)** | 0 = No | 1 = Yes |
| **26.1.3** | | Other (please specify below) | 0 = No | 1 = Yes | **25.1.7** | Steroid creams | 0 = No | 1 = Yes |
| **26.1.4** | | Home nebuliser | 0 = No | 1 = Yes | **25.1.8** | Adrenalin auto injector or pen e.g. Epipen | 0 = No | 1 = Yes |
| **Q26.2** | If your child is on any other oral medication (pills or syrups) for asthma, please **specify** | | | | Free text | | | |

| | | |
|---|---|---|
| | **each one**.<br>(e.g. oral steroids or leukotriene receptor antagonists) | |
| **Q26.3** | If your child is on antihistamines, **please specify** which type/brand? | Free text |

| | | | | |
|---|---|---|---|---|
| **Q26.4** | If your child is on antihistamines, how many days since they were last taken? | **1 =**<br><2 days | **2 =**<br>2-5 days | **3 =**<br>>5 days |
| **Q27.5** | If your child is on any other medication for other illnesses, please specify each one. | Free text | | |

**Q27 Family history of allergic disease**

| Does anyone in your family have allergic diseases? Please circle.<br>(you can choose more than one option) | | | | | | |
|---|---|---|---|---|---|---|
| | Family member | None | Asthma | Hay fever | Eczema | Food allergy |
| **Q27.1** | Mother | 1 = none | 2 = asthma | 3 = hay fever | 4 = eczema | 5 = food allergy |
| **Q27.2** | Father | 1 = none | 2 = asthma | 3 = hay fever | 4 = eczema | 5 = food allergy |
| **Q27.3** | Full Sibling 1 | 1 = none | 2 = asthma | 3 = hay fever | 4 = eczema | 5 = food allergy |
| **Q26.4** | Full Sibling 2 | 1 = none | 2 = asthma | 3 = hay fever | 4 = eczema | 5 = food allergy |
| **Q27.5** | Full Sibling 3 | 1 = none | 2 = asthma | 3 = hay fever | 4 = eczema | 5 = food allergy |
| **Q27.6** | Full Sibling 4 | 1 = none | 2 = asthma | 3 = hay fever | 4 = eczema | 5 = food allergy |

**Q28 Child's Medical history**

| | | | |
|---|---|---|---|
| **Q28.1** | Does your child have any other significant medical problems? (e.g. heart or lung problems, kidney or liver disease, epilepsy, diabetes) | **0 = No** | **1 = Yes** |
| **Q28.2** | If yes, please specify | | |

**Q29 Home Language and Migration**

| | | | | |
|---|---|---|---|---|
| **Q29.1** | What language do you mainly speak at home? (Please choose only one) | | | |
| | **1** = IsiXhosa | **2** = English | **3** = Afrikaans | |
| | **4** = IsiZulu | **5** = Sesotho | **6** = Setswana | |
| | **7** = SiSwati | **8** = IsiNdebele | **9**=Xitsonga | |
| | **10** = Sepedi | **11**=Tshivenda | **12** = Other | |
| **Q29.1.2** | If Xhosa speaking, was this child born in Cape Town? | | **0 = No** | **1 = Yes** |

| | | | | | |
|---|---|---|---|---|---|
| 29.2.1 | If No, where was this child born? | Province | | | |
| | | 1 = Western Cape<br>2 = Eastern Cape<br>3 = Northern Cape<br>4 = Free state<br>5 = Gauteng | | 6 = Kwazulu Natal<br>7 = Mpumalanga<br>8 = Limpopo<br>9 = North West Province<br>10 = Other | |
| | | Town (specify) | | Free text | |
| 29.2.2 | When did this child move to Cape Town? | | Year | | Month |

| | | | | | |
|---|---|---|---|---|---|
| Q29.3 | Was this child’s mother born in Cape Town? | | | 0 = No | 1 = Yes |
| 29.3.1 | **If No,** where was she born? | Province (please circle) | | | |
| | | 1 = Western Cape<br>2 = Eastern Cape<br>3 = Northern Cape<br>4 = Free state<br>5 = Gauteng | | 6 = Kwazulu Natal<br>7 = Mpumalanga<br>8 = Limpopo<br>9 = North West Province<br>10 = Other | |
| | | Town (specify) | | Free text | |
| 29.3.2 | **If no** when did she move to Cape Town? | | Year | | Month |
| 29.3.3 | Was this Child’s father born in Cape Town? | | | 0 = No | 1 = Yes |
| | **If No,** where was he born? | Province | | | |
| | | 1 = Western Cape<br>2 = Eastern Cape<br>3 = Northern Cape<br>4 = Free state<br>5 = Gauteng | | 6 = Kwazulu Natal<br>7 = Mpumalanga<br>8 = Limpopo<br>9 = North West Province<br>10 = Other | |
| | | Town(specify) | | | |

**Q30 Ethnicity**

| | | | |
|---|---|---|---|
| Q30 | What is your child’s ethnic origin? (circle as appropriate) | **1** = White/Caucasian | **2** = Coloured / Mixed race |
| | | **3** = Black African | **4** = Asian/Indian |
| | | **5** = Other (Specify) | |

**Q30 Household information**

| | | |
|---|---|---|
| Q31.1 | How many people live together in your house? | |

| | | |
|---|---|---|
| **Q31.2** | How many children (12 years or less) that are OLDER than this child live in the same household? | |
| **Q31.3** | How many children that are YOUNGER than this child live in the same household? | |

**Q32 Parental Education level**

| | |
|---|---|
| **1** = None | **11** = Grade 9 / Std 7 |
| **2** = Grade R / preschool | **12** = Grade 10 / Std 8 |
| **3** = Grade 1 / SubA | **13** = Grade 11 / Std 9 |
| **4** = Grade 2 / Sub B | **14** = Grade 12 / Matric |
| **5** = Grade 3 / Std 1 | **15** = Grade 9,10,11 (Std 7,8,9) & diploma |
| **6** = Grade 4 / Std 2 | **16** = Grade 12 (Std 10) & Certificate or Diploma |
| **7** = Grade 5 / Std 3 | **17** = Grade 12 (Std 10) & Degree |
| **8** = Grade 6 / Std 4 | **18** = Grade 12 (Std 10) & Degree plus Diploma or further degree |
| 9 = Grade 7 / Std 5 | **19** = Grade 12 (Std 10) & PhD |
| **10** = Grade 8 / Std 6 | Other (**Specify) :** Free text |

**Q33 Household income**

| | | | |
|---|---|---|---|
| **Q33.1** | What job does this child's mother/female guardian do? | | |
| **Q33.2** | What job does this child's father/male guardian do? | | |
| **Q33.3** | How much money or income does your household receive every month after tax? (incl. money from work, pensions, informal business etc.) | R | 999 = Don't know |

**Q34 Contact with pets and animals**

| | | | | |
|---|---|---|---|---|
| **Q34.1** | Do you own a cat or have a cat in your home? | | **0 = No** | **1 = Yes** |
| **Q34.2** | Do you own a dog or have a dog in your home? | | **0 = No** | **1 = Yes** |
| **Q34.3** | Does your child have regular (at least once a week) contact with farm animals (e.g. cattle, pigs, goats, sheep or poultry)? | **0 = No** | **1 = Yes** | **999** = Don't know |
| **Q34.4** | Has this child's mother had regular (at least once a week) contact with farm animals (e.g. cattle, pigs, goats, sheep or poultry) while being pregnant with this child? | **0 = No** | **1 = Yes** | **999** = Don't know |

**Q35 Fuel exposure**

| | | |
|---|---|---|
| **Q35.1** | At your house, what fuel is used for cooking? | |
| | **1** = Electricity/Gas | **4** = Open fires outside the house |
| | **2** = Paraffin Stove | **5** = Other (specify) |
| | **3** = Open fires in the house | |
| **Q35.2** | At your house, what fuel is used for heating? | |
| | **1** = Electricity | **4** = Wood/coal |
| | **2** =Gas | **5** = Other (specify) |
| | **3** =Kerosene/Paraffin | |

**Q36 Cigarette smoke exposure**

| | | | | |
|---|---|---|---|---|
| **Q36.1** | Does this child's mother (or female guardian) currently smoke cigarettes? | **0 = No** | **1 = Yes** | **3** = Don't know |
| **36.1.1** | If **YES**, about how many cigarettes does the child's mother (or female guardian) smoke each day? | number of cigarettes: | | |
| **Q36.2** | Does this child's father (or male guardian) currently smoke cigarettes? | **0 = No** | **1 = Yes** | **3** = Don't know |
| **36.2.1** | If **YES**, about how many cigarettes does the child's father (or male guardian) smoke each day? | number of cigarettes: | | |
| **Q36.3** | How many people living in the house currently smoke cigarettes, including parents? | No of people: | | |
| **Q36.4** | Did this child's mother smoke cigarettes while being pregnant with this child? | **0 = No** | **1 = Yes** | **3** = Don't know |
