# Supplementary material for: Multimodal protective and susceptibility clusters in paediatric atopic dermatitis: A machine learning-based, data-driven observational study
Source: PLoS Med. 2026 Sep 8;23(9):e1004917. doi: 10.1371/journal.pmed.1004917 (PMC13552787; doi:10.1371/journal.pmed.1004917)
Supplement: Supplementary file 3 [file pmed.1004917.s003.pdf]

S2 File

SOS-ALL Machine Learning Methods

In [5]:
```
import os
import pandas as pd
from pathlib import Path
from IPython.display import display, Image
```

1. Data

- Base-file for the analysis is `DataTransformed_ImmunoCAP_Final_value_report_f_dtypes.csv` received from Damir Zhakparov / Katja Baerenfaller. The file was used in combination with `DataTransformed_ImmunoCAP_Final.txt`

In [6]:
```
project_directories = os.getcwd()

data_path = os.path.join(project_directories, 'data', 'input', 'DataTransformed_
path_data_setup = os.path.join(project_directories, 'data', 'input', 'DataTransf

data = pd.read_csv(data_path, sep='\t', index_col=0)
data_setup = pd.read_csv(path_data_setup, sep=";", index_col=0)
```

In [7]:
```
data
```

Out[7]:

	PID	diagnosis	location	enrolment_age	gender	vaccination_status	paracetamol_
**0**	CA-001-LS	AD	Urban	24	2.0	1.0	
**1**	CA-002-YF	AD	Urban	14	1.0	1.0	
**2**	CA-003-US	AD	Urban	20	2.0	1.0	
**3**	CA-004-NK	AD	Urban	14	1.0	1.0	
**4**	CA-005-BD	AD	Urban	17	2.0	1.0	
**...**	...	...	...	...	...	...	
**212**	CO-149-LM	HC	Rural	13	2.0	1.0	
**213**	CO-150-LN	HC	Rural	26	1.0	1.0	
**214**	CO-151-AN	HC	Rural	23	1.0	1.0	
**215**	CO-152-AF	HC	Rural	26	1.0	1.0	
**216**	CO-153-YM	HC	Rural	16	2.0	1.0	

217 rows × 139 columns

In [8]:

```
data_setup
```

Out[8]:

	variable	value_counts	unique_values	nan_counts	nan_perce
**1**	PID	{'CA-001-LS': 1, 'CO-035-SM': 1, 'CO-024-KM': ...	217.0	0.0	0.0
**2**	diagnosis	{'AD': 116, 'HC': 101}	2.0	0.0	0.0
**3**	location	{'Rural': 112, 'Urban': 105}	2.0	0.0	0.0
**4**	enrolment_age	{17: 14, 14: 13, 12: 12, 19: 11, 23: 10, 21: 1...	30.0	0.0	0.0
**5**	gender	{1.0: 120, 2.0: 96}	3.0	1.0	0.4
**...**	...	...	...	...	
**135**	log_blood_counts_monocytes	{-0.371063681: 6, -0.356674944: 5, 0.039220713...	115.0	43.0	19.8
**136**	log_blood_counts_lymphocytes	{1.373715579: 3, 1.526056303: 3, 1.870262531: ...	148.0	44.0	20.2
**137**	log_blood_counts_eosinophils	{-2.302585093: 8, -1.078809661: 5, -1.60943791...	104.0	41.0	18.8
**138**	diagnosis_location	{'AD_Rural': 60, 'AD_Urban': 56, 'HC_Rural': 5...	4.0	0.0	0.0
**139**	RNASeq	{'yes': 149, '0': 68}	2.0	0.0	0.0

139 rows × 7 columns

1.1 Removing unnecessary features

All features with a value=1 in the 'exclude'column of the file `DataTransformed_ImmunoCAP_Final.txt` were excluded from all subsequent analyses. Some features were excluded after initial analyses and subsequent discussions as part of an iterative process. In total, the following **45 features** were **excluded**:

['vaccination_status', 'antibiotic_exposure_age', 'solidfood_which_first', 'peanuts_exposure_age_first', 'peanuts_exposure_regularity', 'othernuts_exposure_age_first', 'othernuts_exposure_regularity', 'fish_exposure_age_first', 'fish_exposure_regularity', 'eczema_ever', 'eczema_age', 'eczema_whodiagnosed', 'medication_any', 'medication_nasalcorticosteroid', 'medication_antihistamines', 'medication_steroidcreams', 'medication_oral_which', 'medication_antihistamines_last', 'medicalhistory_comorbidities', 'parental_education_level', 'parental_income', 'am_height', 'am_weight', 'am_abd_girth', 'am_skinfold', 'scorad', 'bsa', 'country', 'familyhistory_sibling_eczema', 'familyhistory_sibling_other_allergic_disease', 'diagnosis_location', 'RNASeq', 'location', 'log_blood_counts_wcc', 'stool_katokatz', 'sp_egg', 'sp_milk', 'sp_soy', 'sp_wheat', 'sp_fish', 'sp_peanut', 'sp_hazelnut', 'sp_fresh_peanut', 'sp_fresh_egg', 'sp_fresh_milk']

1.2 Setting feature types

Features were converted to the appropriate data types based on the "dtype" column in `DataTransformed_ImmunoCAP_Final.txt` column 'dtype'. The "PID" column was set as the index for the analysis and was not used as a predictive feature.

1.3 Shuffling observations

All observations were shuffled to ensure that training and validation sets do not contain only one group of data.

1.4 Setting the target variable

The "diagnosis" column was defined as the target variable (y). The values were recoded from "AD" and "HC" to 1 (AD) and 0 (HC), respectively.

1.5 Final training data

After applying all the steps described above, the `training data` were structured as follows:

In [9]:

```
data_path = os.path.join(project_directories, 'data', 'output', 'data', 'X_train
X_train = pd.read_pickle(data_path)
X_train
```

Out[9]:

PID	enrolment_age	gender	paracetamol_exposure	paracetamol_exposure_age	parace
CA-123-PG	21	1.0	1.0	3.0	
CA-034-LS	29	2.0	1.0	5.0	
CA-035-AR	15	1.0	1.0	6.0	
CA-107-BM	25	1.0	1.0	7.0	
CA-142-AM	21	2.0	1.0	3.0	
...	...	...	...	...	
CO-140-IM	19	1.0	1.0	6.0	
CO-023-AN	17	2.0	1.0	7.0	
CA-117-SM	25	1.0	0.0	NaN	
CO-026-LT	17	2.0	1.0	NaN	
CA-038-LM	16	2.0	1.0	4.0	

217 rows × 92 columns

In [10]:

```
data_path = os.path.join(project_directories, 'data', 'output', 'data', 'y_train
y_train = pd.read_pickle(data_path)
y_train
```

```
Out[10]: PID
CA-123-PG    1.0
CA-034-LS    1.0
CA-035-AR    1.0
CA-107-BM    1.0
CA-142-AM    1.0
            ...
CO-140-IM    0.0
CO-023-AN    0.0
CA-117-SM    1.0
CO-026-LT    0.0
CA-038-LM    1.0
Name: diagnosis, Length: 217, dtype: float64
```

No train–test split was performed, as all data were used for model development via cross-validation. The dataset was therefore used for both training and validation of all models.

2. Modelling

The goal of the analysis was not simply to build the "best" model (and obtain a score), but to identify useful and meaningful features that are important for model building. Ideally, these features should be relatively stable across different models and/or model groups. This would indicate that they are true predictors rather than being specific to a particular model (or its hyperparameters). To achieve this, several steps were implemented.

2.1 Optuna (framework)

`Optuna` was used as a framework to perform hyperparameter optimisation (https://optuna.org/). This framework allows the definition of search spaces for hyperparameters, rather than fixed values as in grid search. After defining these hyperparameter spaces, either a number of trials or a time budget is specified. Based on these settings, Optuna attempts to identify the "best" model using a **TPESampler**. Unlike grid search, where all possible combinations of hyperparameter settings are evaluated, the TPE approach focuses on parameter settings that are more likely to achieve high performance. TPE stands for Tree-structured Parzen Estimator. On each trial, for each parameter, TPE fits one Gaussian Mixture Model (GMM) l(x) to the set of parameter values associated with the best objective values, and another GMM g(x) to the remaining parameter values. It then selects the parameter value x that maximises the ratio l(x)/g(x) (source:
https://optuna.readthedocs.io/en/stable/reference/samplers/generated/optuna.samplers.TPE

The **TPESampler** was applied not only to optimise hyperparameters within a single model, but also across different models (algorithms). In this study, the following algorithms (and corresponding feature spaces) were used:

- **RandomForestClassifier** (sklearn.ensemble.RandomForestClassifier):

```
params = {
            'n_estimators': trial.suggest_int('rf__n_estimators', 
50, 500),
            'max_depth': trial.suggest_categorical('rf__max_depth', 
[None, 1, 2, 3, 4, 5])
        }
```

- **MLP** (sklearn.neural_network.MLPClassifier):

```
params = {
            'hidden_layer_sizes': 
tuple([trial.suggest_int(f'mlp__layer_{i}_size', 1, 200) for i in 

range(trial.suggest_int('mlp__n_layers', 1, 5))]),
            'alpha': trial.suggest_float('mlp__alpha', 0.00001, 
0.001, log=False),
            'activation': 
trial.suggest_categorical('mlp__activation', ['logistic', 'tanh', 
'relu']),
            'solver': trial.suggest_categorical('mlp__solver', 
['lbfgs', 'sgd', 'adam'])
        }
```

- **GNB** (sklearn.naive_bayes.GaussianNB)

```
params = {
            'var_smoothing': 
trial.suggest_float('gnb__var_smoothing', 1e-10, 1e-7, log=True)
        }
```

- **XGBOOST** (xgboost.XGBClassifier)

```
params = {
            'max_depth': trial.suggest_int('xgb__max_depth', 1, 9),
            'learning_rate': 
trial.suggest_float('xgb__learning_rate', 0.01, 1.0, log=True),
            'n_estimators': trial.suggest_int('xgb__n_estimators', 
50, 500),
            'min_child_weight': 
trial.suggest_int('xgb__min_child_weight', 1, 10),
            'gamma': trial.suggest_float('xgb__gamma', 1e-8, 1.0, 
log=True),
            'subsample': trial.suggest_float('xgb__subsample', 
0.01, 1.0, log=True),
            'colsample_bytree': 
trial.suggest_float('xgb__colsample_bytree', 0.01, 1.0, log=True),
            'reg_alpha': trial.suggest_float('xgb__reg_alpha', 1e-
8, 1.0, log=True),
            'reg_lambda': trial.suggest_float('xgb__reg_lambda', 
1e-8, 1.0, log=True)
        }
```

2.2 Pipeline

The training data currently contain missing values. For some models, these missing values need to be replaced with valid numerical values to allow model training. In

addition, preprocessing steps such as normalisation or scaling are useful for certain algorithms, for example MLP. To account for these requirements and to reduce the risk of data leakage, an `sklearn.pipeline` was constructed.

2.2.1 Imputation

Imputation of missing values is one of the most important steps in the prediction process in this study. Replacing missing values with simple group means or medians, as commonly done, would lead to distorted results in this case. This is because missing values are not equally distributed between "AD" and "HC", and for several features there is a clear difference in the number of missing values between these groups. If missing values are replaced by a constant value (e.g. group mean or median), the model may use this as an additional signal, leading to biased predictions. For this reason, the `miceforest` package was used. This imputation method applies a RandomForest-based approach to estimate missing values for each feature based on the values of other features.

The following parameters were used for `miceforest`:

```
    mice_initialization = trial.suggest_categorical('mice_init',
['random', 'empty'])
    mice_iterations = trial.suggest_int("mice_iter", 1, 5)
```

The initialisation can be either empty or random, and between 1 and 5 iterations are applied to estimate missing values for each feature.

2.2.2 Scaling

Some algorithms perform better when features are scaled. Therefore, the pipeline included a scaling step with the following parameters:

```
    trial.suggest_categorical('scaler', ['StandardScaler',
'MinMaxScaler', 'passthrough'])
```

`passthrough` indicates that no scaling was applied

2.2.3 Quasi-constant features

Quasi-constant features are features with no or very little variation and therefore provide little useful information. These features were excluded from the analysis. They could not be removed during preprocessing, as the lack of variation depends on the train–validation split during cross-validation.

```
    trial.suggest_categorical('quasi_constant',
['variance_threshold', 'passthrough'])
```

In case the 'variance_threshold' is used be the Sampler (see next section) the parameter-space for 'variance_threshold' is:

```
    params = {
                'threshold':
trial.suggest_float('quasi_constant__variance_threshold', 0,
0.15),
            }
```

2.2.5 Feature selection

The training data contain a large number of features, which increases the risk of overfitting when all features are used. Therefore, a feature selection step was included in the pipeline.

```
    sklearn.feature_selection.SelectKBestSelectKBest(k), with
k=trial.suggest_int("selection_kbest_features", 5, 30)
```

This means that at least 5 features and at most 30 features were selected in each model run.

After applying all steps, the pipeline was structured as follows:

```
pipe = Pipeline([
                ('mice_imputer', 'mice_imputer'),
                ('knn_imputer', knn_imputer),
                ('scaler', scaler),
                ('selection', selection),
                ('quasi_constant', constant),
                ('model', model)
            ])
```

Remark: The `knn_imputer` applied after the `mice_imputer` is necessary because, in some cases (depending on the train-test split and very few missing values in a feature), it is possible that not missing values are present in the training data. In such cases, the `mice_imputer` in cannot estimate missing values for that feature. To address this, an additional `nn_imputer` imputation step was applied to ensure that all missing values are replaced. This issue affects only very few values (if any), therefore the `knn_imputer` can be used without introducing the data leakage mentioned above.

2.3 Cross-validation

The entire pipeline was evaluated for each hyperparameter combination using a 5-fold cross-validation process. The mean accuracy across the five test folds was used as the final score for each combination. Accuracy was chosen as the evaluation metric because the distribution of "AD" and "HC" in the dataset is nearly balanced.

3. Results

In this study, **77 different models** (with different hyperparameter settings) were evaluated. All models were stored so they could be analysed later using SHAP values.

Remark: Each model was refitted on the full dataset using its respective hyperparameter settings after cross-validation. The cross-validation score therefore serves as an indicator of how well the model is expected to perform on unseen data (accuracy). SHAP values were calculated using the full dataset.

In [11]:
```
results
```

Out[11]:

	State	Group	Disc	Number	Value	Datetime_start	Datetime_complete	Dur
**0**	COMPLETE	NaN		0	0.755814	2023-10-01 17:46:27	2023-10-01 17:58:34	0:
**1**	COMPLETE	NaN		1	0.465011	2023-10-01 17:58:34	2023-10-01 18:05:44	0:
**2**	COMPLETE	NaN		2	0.465011	2023-10-01 18:05:44	2023-10-01 18:16:01	0:
**3**	COMPLETE	NaN		3	0.728224	2023-10-01 18:16:01	2023-10-01 18:18:27	0:
**4**	COMPLETE	NaN		4	0.746829	2023-10-01 18:18:27	2023-10-01 18:30:48	0:
**...**	...	...	...	...	...	...	...	
**73**	COMPLETE	NaN		73	0.802114	2023-10-02 02:54:17	2023-10-02 03:05:43	0:
**74**	COMPLETE	NaN		74	0.797357	2023-10-02 03:05:43	2023-10-02 03:17:20	0:
**75**	COMPLETE	NaN		75	0.788161	2023-10-02 03:17:20	2023-10-02 03:28:43	0:
**76**	COMPLETE	NaN		76	0.534989	2023-10-02 03:28:43	2023-10-02 03:40:16	0:
**77**	COMPLETE	NaN		77	0.802008	2023-10-02 03:40:16	2023-10-02 03:52:06	0:

78 rows × 35 columns

The best **accuracy score (0.81)** was achieved by multiple Random Forest models.

In [12]:
```
display(Image(filename="data/output/overview.png", width='75%'))
```

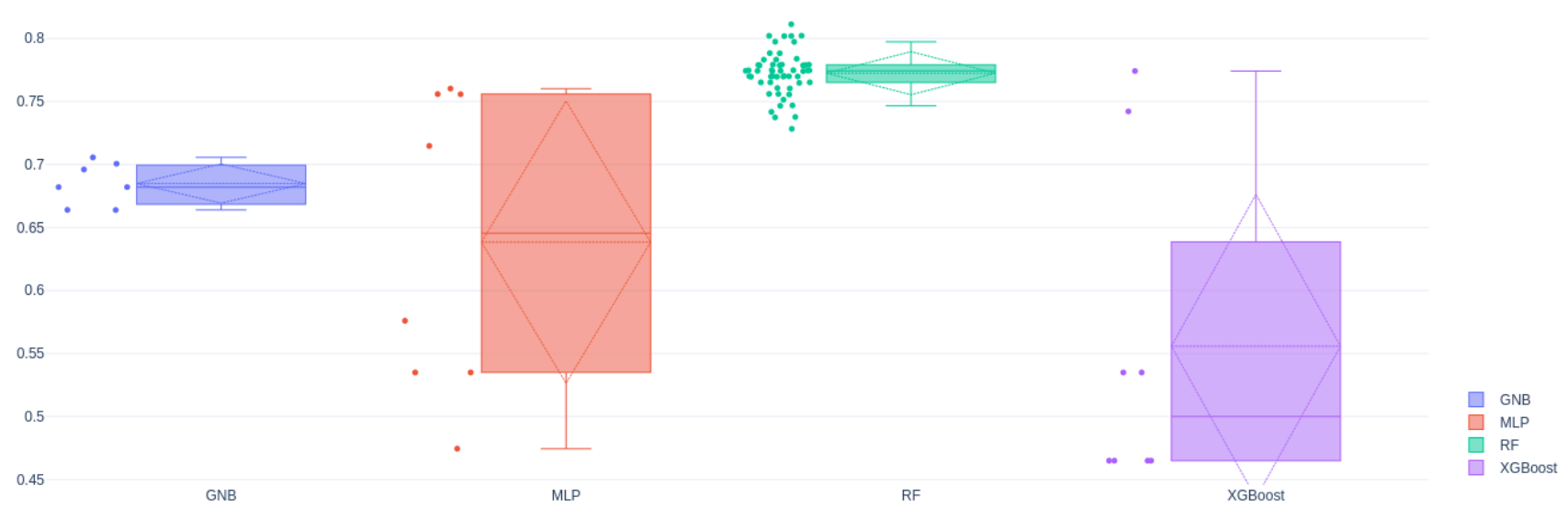

The best model performance was achieved after **52 trials**.

In [13]:
```
display(Image(filename="data/output/model_performance_development.png", width='7
```

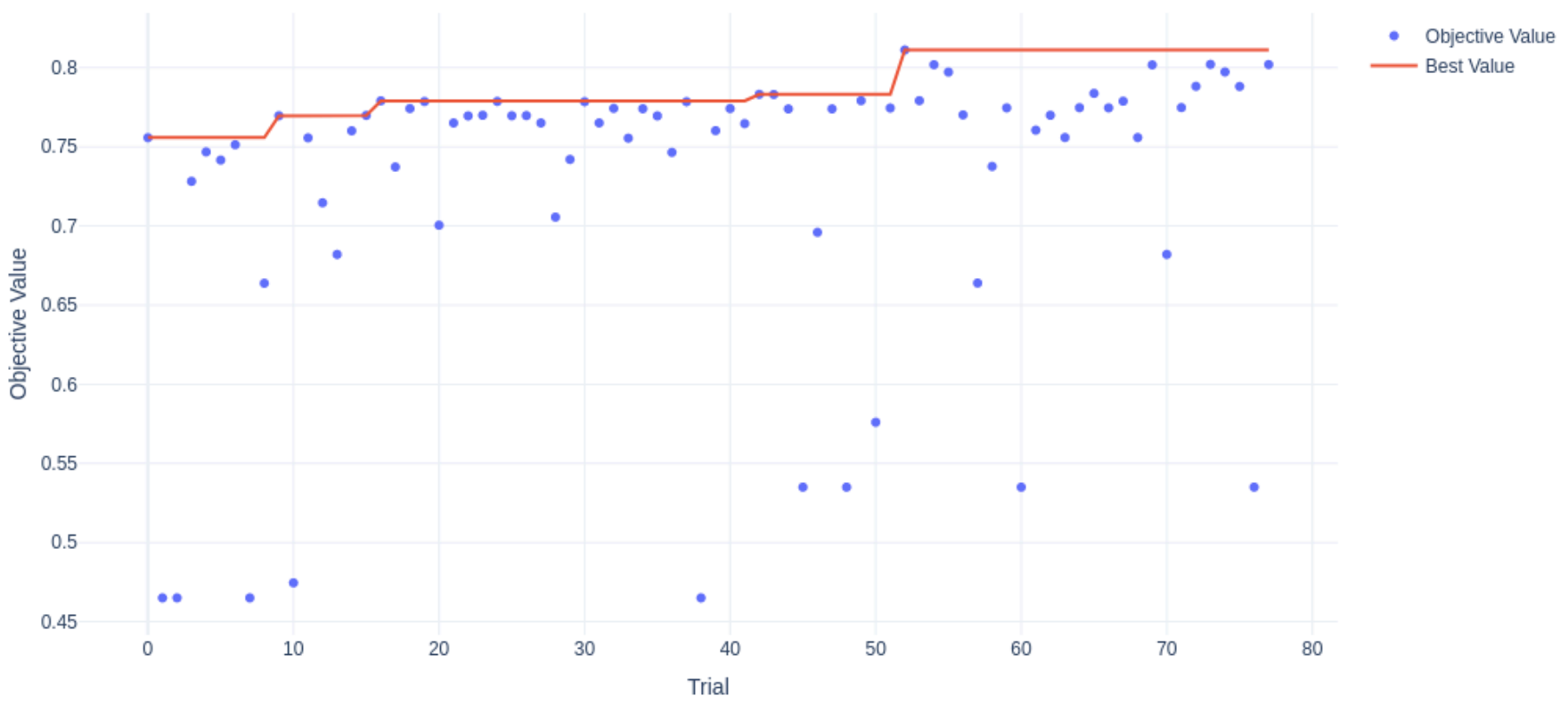

Interestingly, most (almost all) well-performing Random Forest (RF) models have a max_depth parameter of only 1, resulting in very simple trees with only one split.

In [14]:
```
display(Image(filename="data/output/rf_52_max_depth.png", width='75%'))
```

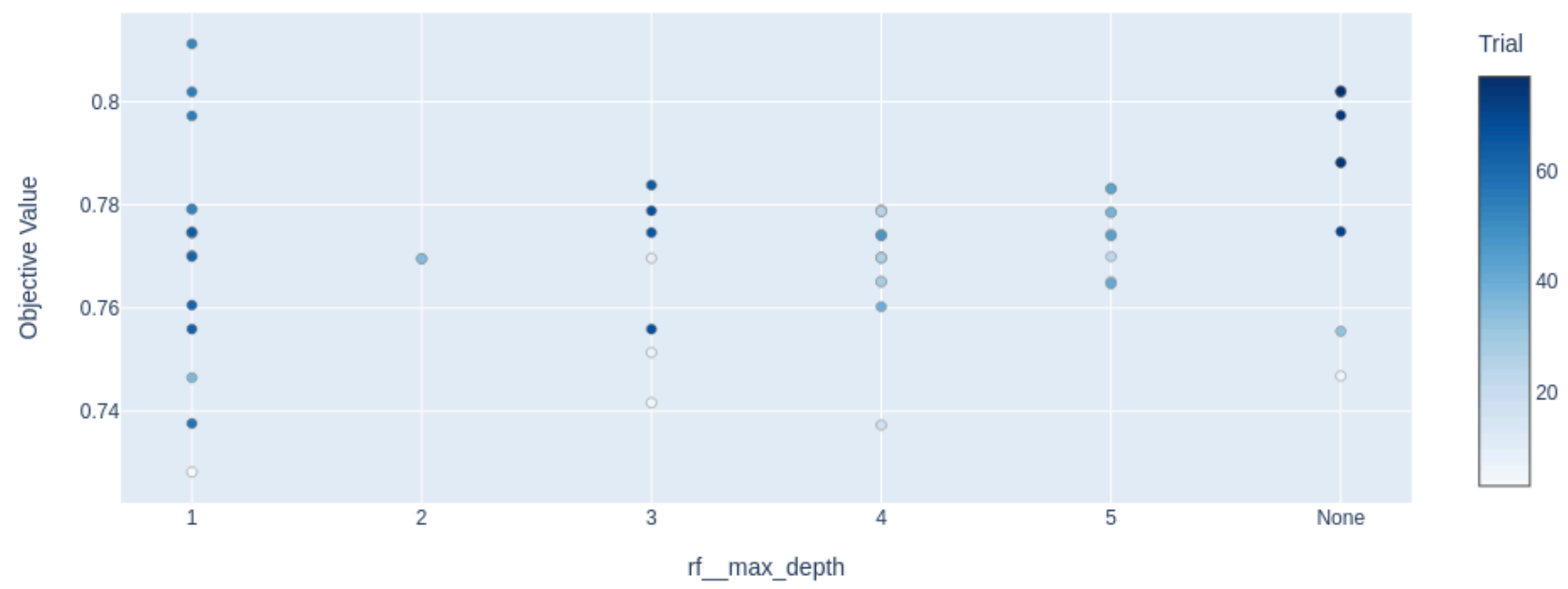

As also visible in the overview plot, the model was the most important parameter in the hyperparameter feature space. Many different Random Forest models achieved high scores. Random Forest outperformed the other model groups in this run.

In [15]:
```
display(Image(filename="data/output/hyperparamter_importance.png", width='75%'))
```

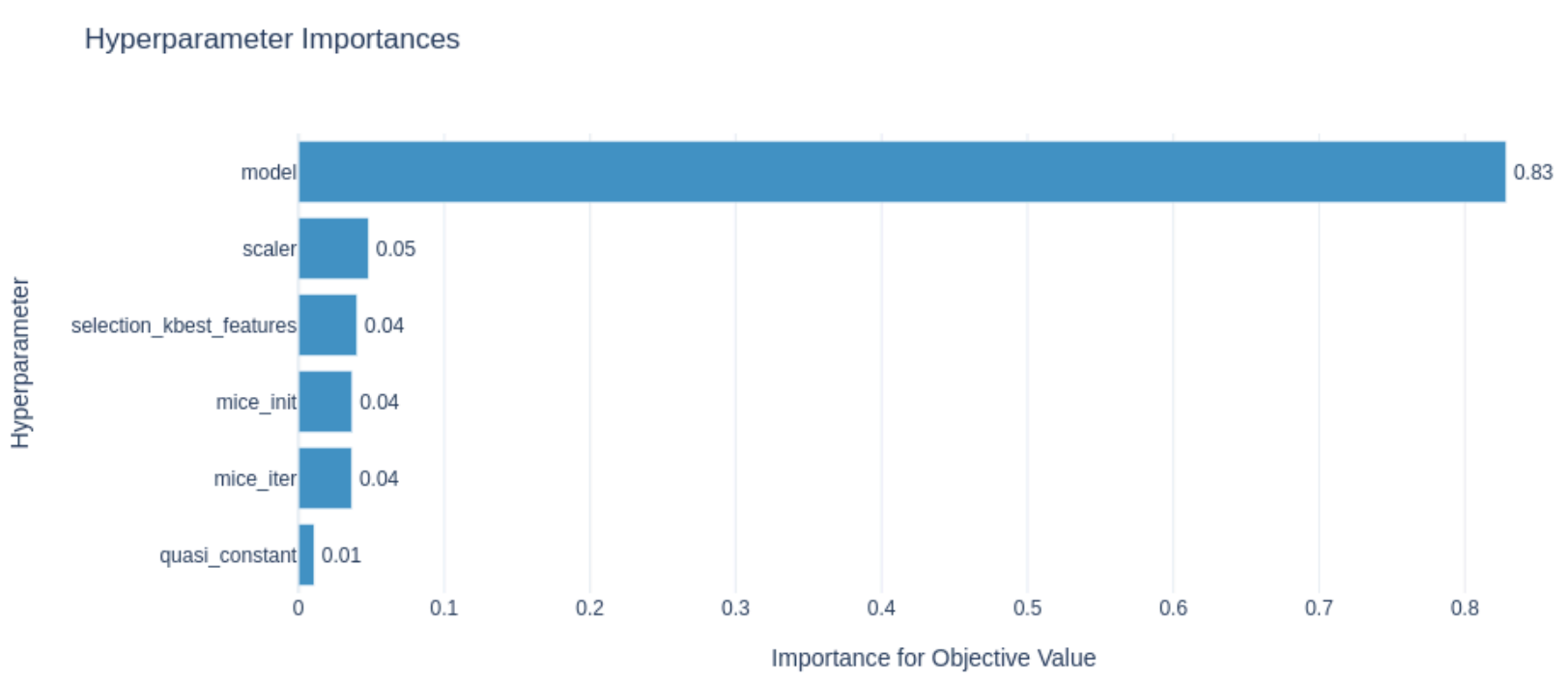

To compare feature stability, the 3 best models from each model group were selected. For all these combinations (4 groups × 3 models), feature stability (method = Jaccard) was calculated and plotted.

Except for trial 48 (XGBOOST) and GBN (trial 28), high scores (> 0.6) were achieved. This is a good indicator that the features do not differ much between the individual trials.

In [16]:
```python
display(Image(filename="data/output/feature_stability_jaccard.png", width='75%'))
```

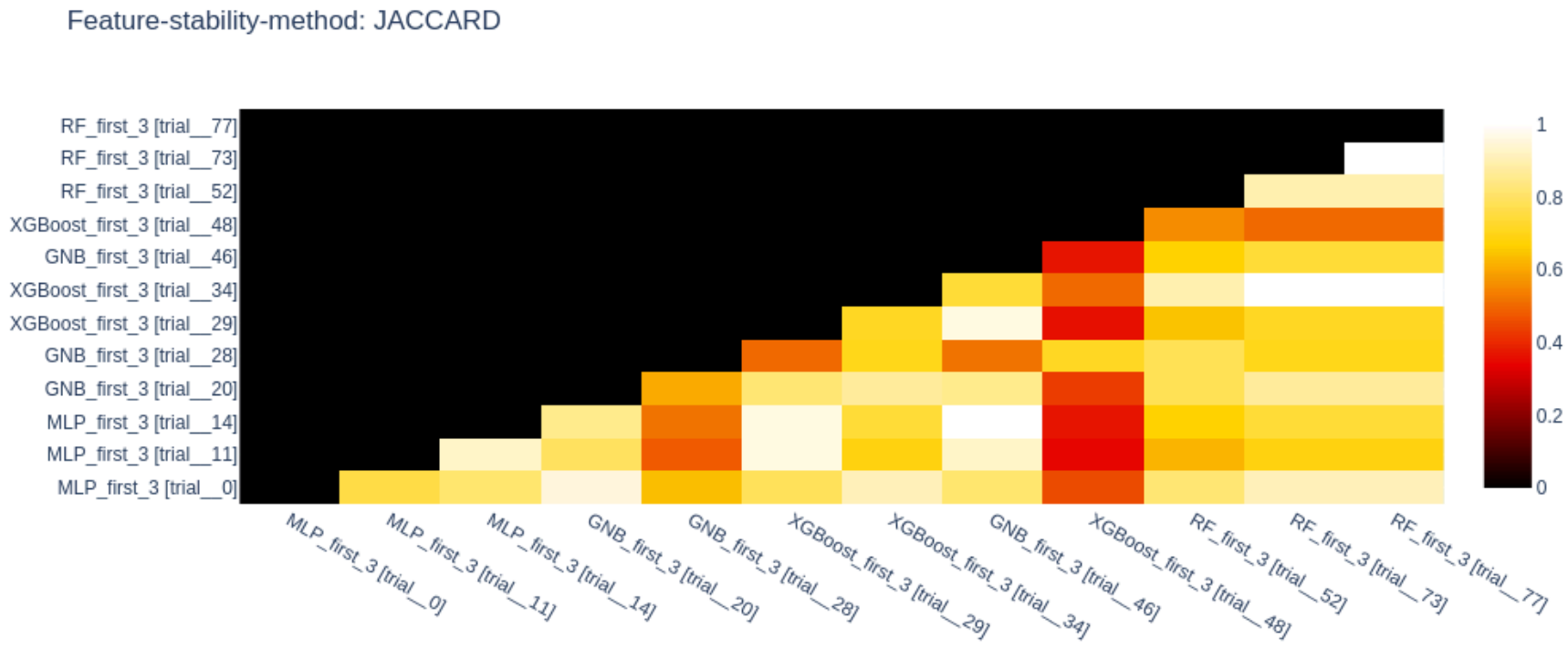

In [17]:
```python
feature_stability
```

Out[17]:

	Nr	Feature	Sum	% Col	GNB_first_3 [trial__20]	GNB_first_3 [trial__28]	G
**0**	0	fuel_heating_Wood_Coal	12	100.00	1	1	
**1**	1	total_IgE	12	100.00	1	1	
**2**	2	fuel_cooking_Electricity_Gas	12	100.00	1	1	
**3**	3	fuel_cooking_Paraffin_Stove	12	100.00	1	1	
**4**	4	fuel_cooking_Open_fires_outside	12	100.00	1	1	
**5**	5	sIgE_Der	12	100.00	1	1	
**6**	6	sIgE_fish	12	100.00	1	1	
**7**	7	sIgG4_Der	12	100.00	1	1	
**8**	8	henegg_exposure_regularity	12	100.00	1	1	
**9**	9	hayfever_ever	12	100.00	1	1	
**10**	10	sIgE_milk	11	91.67	1	1	
**11**	11	paracetamol_exposure_age	11	91.67	1	1	
**12**	12	othernuts_exposure	11	91.67	1	1	
**13**	13	foodreaction_any	11	91.67	1	1	
**14**	14	sunlight_exp_summer	10	83.33	1	0	
**15**	15	familyhistory_mother_eczema	10	83.33	1	0	
**16**	16	henegg_exposure	10	83.33	1	0	
**17**	17	sIgE_egg	10	83.33	1	0	
**18**	18	fuel_cooking_heating_Paraffin	9	75.00	1	0	
**19**	19	familyhistory_father_other_allergic_disease	9	75.00	1	0	
**20**	20	sunlight_exp_winter	6	50.00	1	0	
**21**	21	log_blood_counts_monocytes	6	50.00	1	0	
**22**	22	farmanimal_contact_child	5	41.67	1	0	
**23**	23	pets_dog	4	33.33	0	0	
**24**	24	wheat_exposure_regularity	4	33.33	0	0	
**25**	25	fish_exposure	4	33.33	0	0	
**26**	26	cigarettesmoke_numberofpersons	4	33.33	0	0	
**27**	27	sIgE_soy	2	16.67	0	0	
**28**	28	pets_cat	1	8.33	0	0	

The table above shows that a couple of features (9) were **present in all** (3-best) **models**.

These have been:

```
fuel_heating_Wood_Coal
total_IgE
fuel_cooking_Electricity_Gas
fuel_cooking_Paraffin_Stove
fuel_cooking_Open_fires_outside
sIgE_Der
sIgE_fish
sIgG4_Der
henegg_exposure_regularity
hayfever_ever
```

Also, other features have a great overlap across all models, as can be seen in the table above.

This image shows the Jaccard score for feature stability per model group. The 3 dots per group represent the comparison of all 3 models in this particular group (e.g. RF1 vs RF2, RF1 vs RF3, and RF2 vs RF3). RandomForest (RF) reached the highest scores in feature stability. A Jaccard score of 1 means that all features are present in the compared models.

In [18]:
```python
display(Image(filename="data/output/jaccard_overview.png", width='75%'))
```

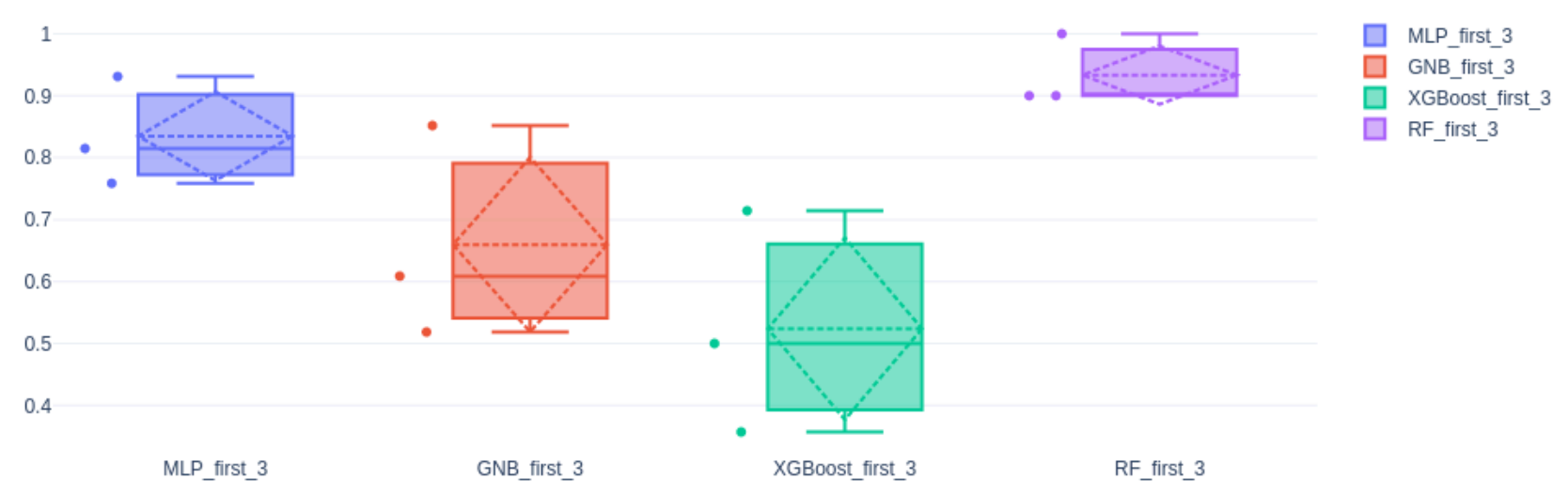

As next step in the analysis, the SHAP values (https://shap.readthedocs.io/en/latest/) for the most promising models were calculated. SHAP values are very handy for explaining models, even if they are "black boxes" (for example MLP). The concept originally comes from game theory, to calculate the contribution of each player (of a game) to the final result. This idea can also be transferred to the importance of features in a classification model.

3.1 SHAP values

3.1.1 Model: ID52 (RandomForest)

The feature importance calculated with SHAP values in Model52 (best model overall) looks like this:

In [19]:
```python
display(Image(filename="data/output/feature_importance_52_shap.png", width='75%'
```

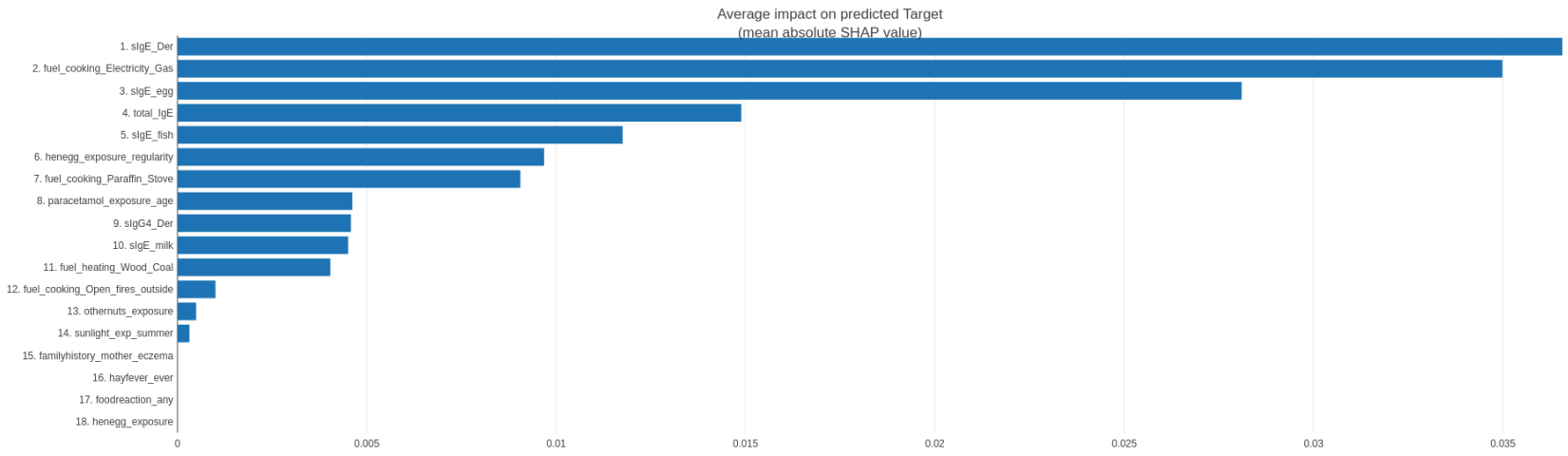

And feature importance using feature permutation:

In [20]:
```
display(Image(filename="data/output/feature_importance_52_permutation.png", widt
```

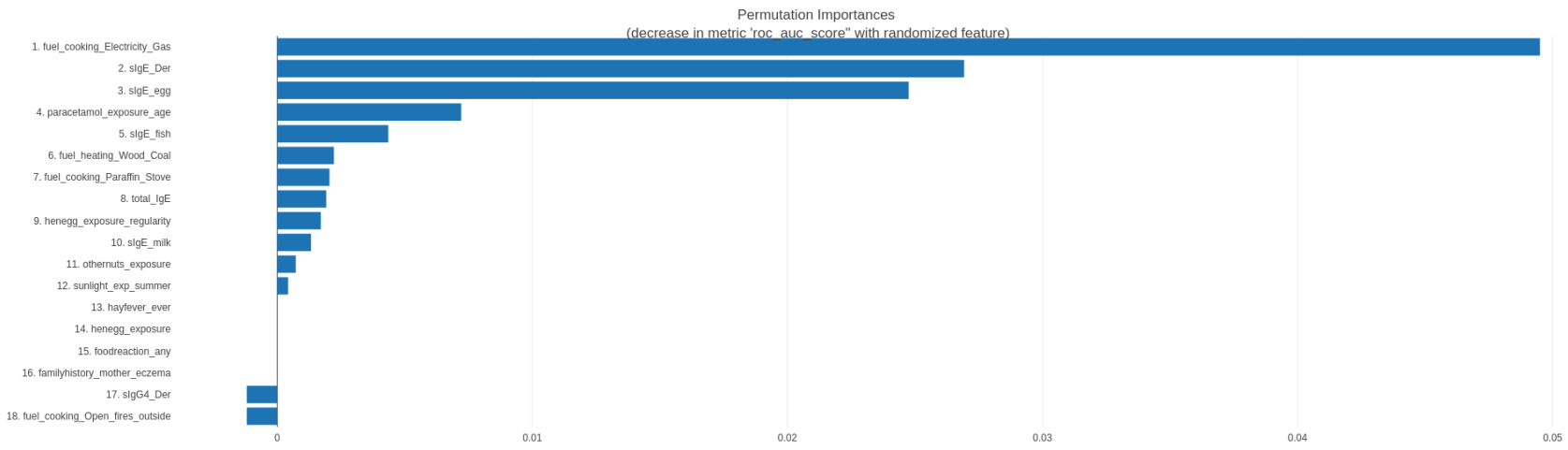

The first 3 features are the same in both analyses, with a different ranking, as can be seen in the plots above:

- sIgE_Der
- fuel_cooking_Electricity_Gas
- sIgE_egg

In [21]:
```
display(Image(filename="data/output/shap_detailed_summary_52.png", width='60%'))
```

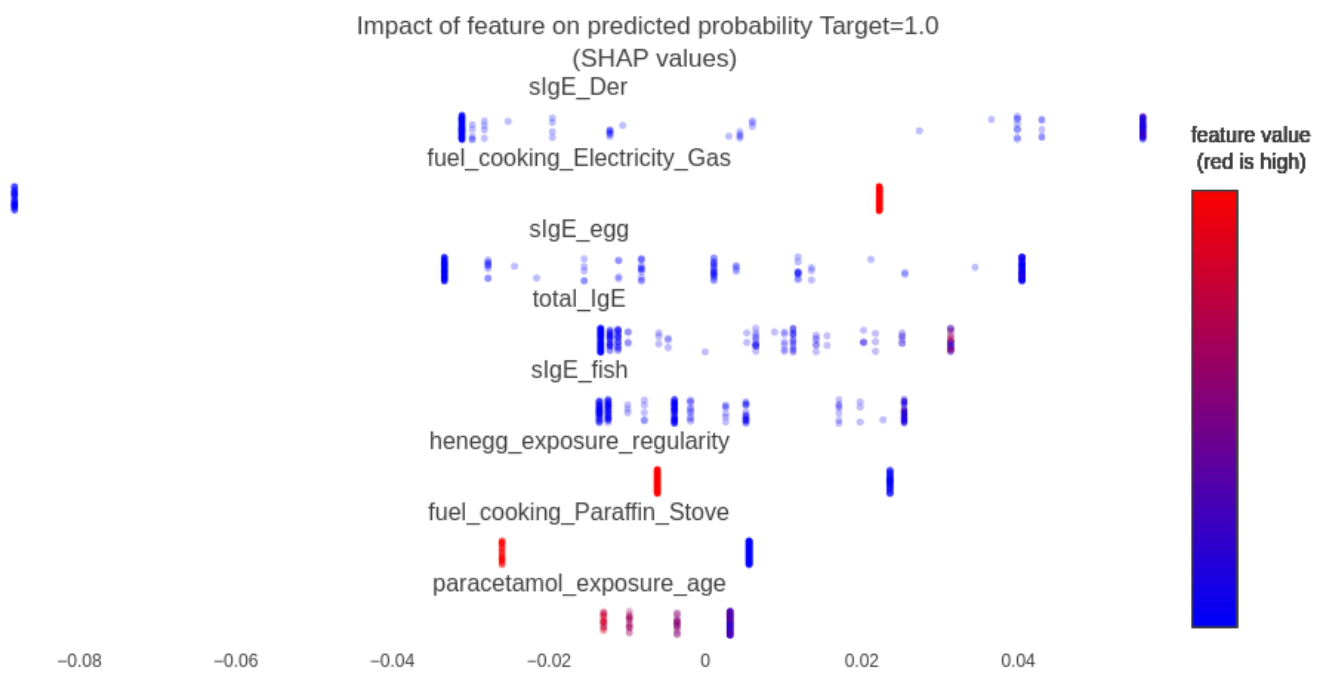

In the SHAP detailed view, the effects on each feature can be analysed:

Very obvious is the effect of a low/high value in `fuel_cooking_Electricity_Gas`. Observations with value = 1 (high) increase their SHAP value, and value = 0 (low) decreases the SHAP value. So observations with value = 1 in `fuel_cooking_Electricity_Gas` tend to have an increased possibility of having `diagnosis` 1 (= AD). The opposity seems to be the case for `henegg_exposure_regularity` and `fuel_cooking_paraffin_stove`. In `total_IgE`, higher feature values seem to correspond to higher SHAP values and therefore a bigger chance of having a `diagnosis` of AD.

3.1.2 Model: ID34 (XGBOOST)

The feature importance calculated with SHAP values in Model34 (best model overall) looks like this:

In [22]:
```
display(Image(filename="data/output/feature_importance_34_shap.png", width='75%'
```

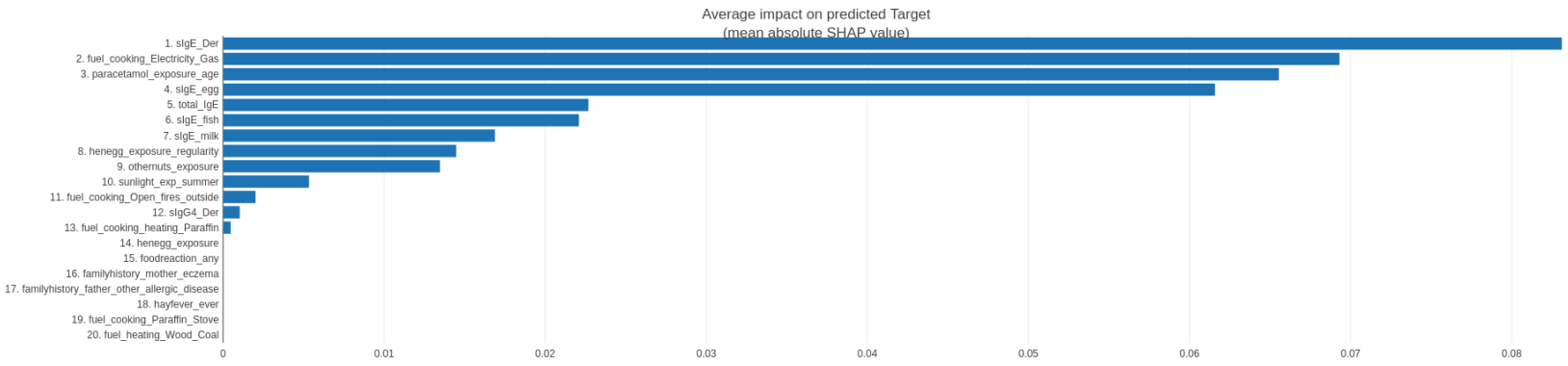

And feature importance using feature permutation:

In [23]:
```
display(Image(filename="data/output/feature_importance_34_permutation.png", widt
```

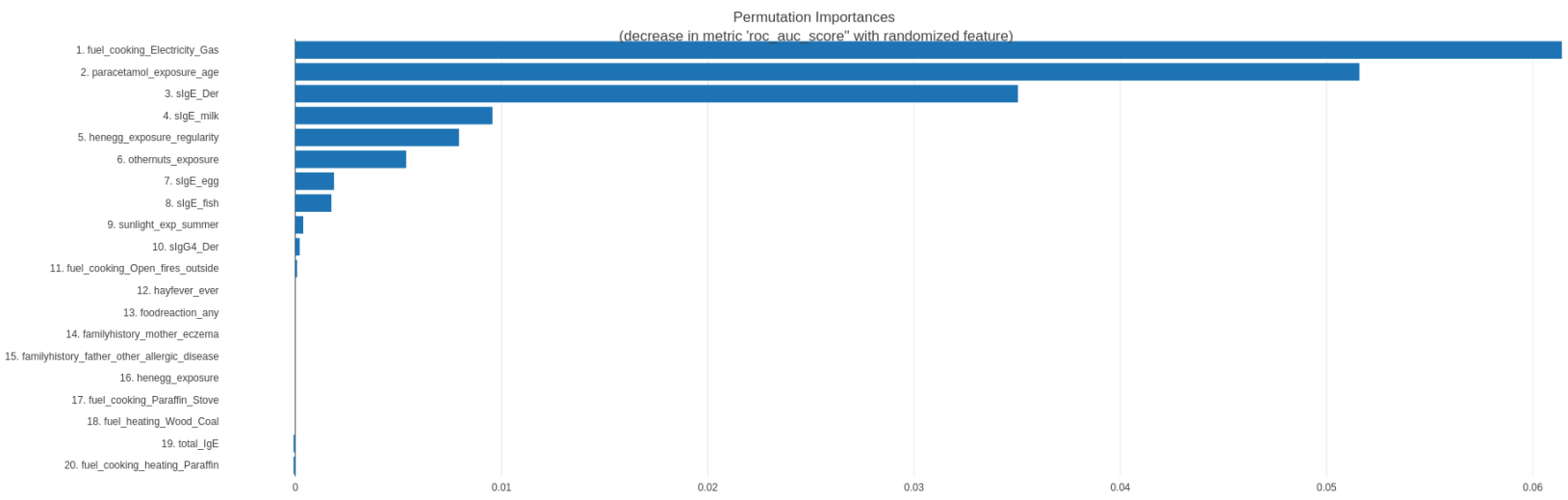

SHAP detailed view (first 8 features):

In [24]:
```
display(Image(filename="data/output/shap_detailed_summary_34.png", width='60%'))
```

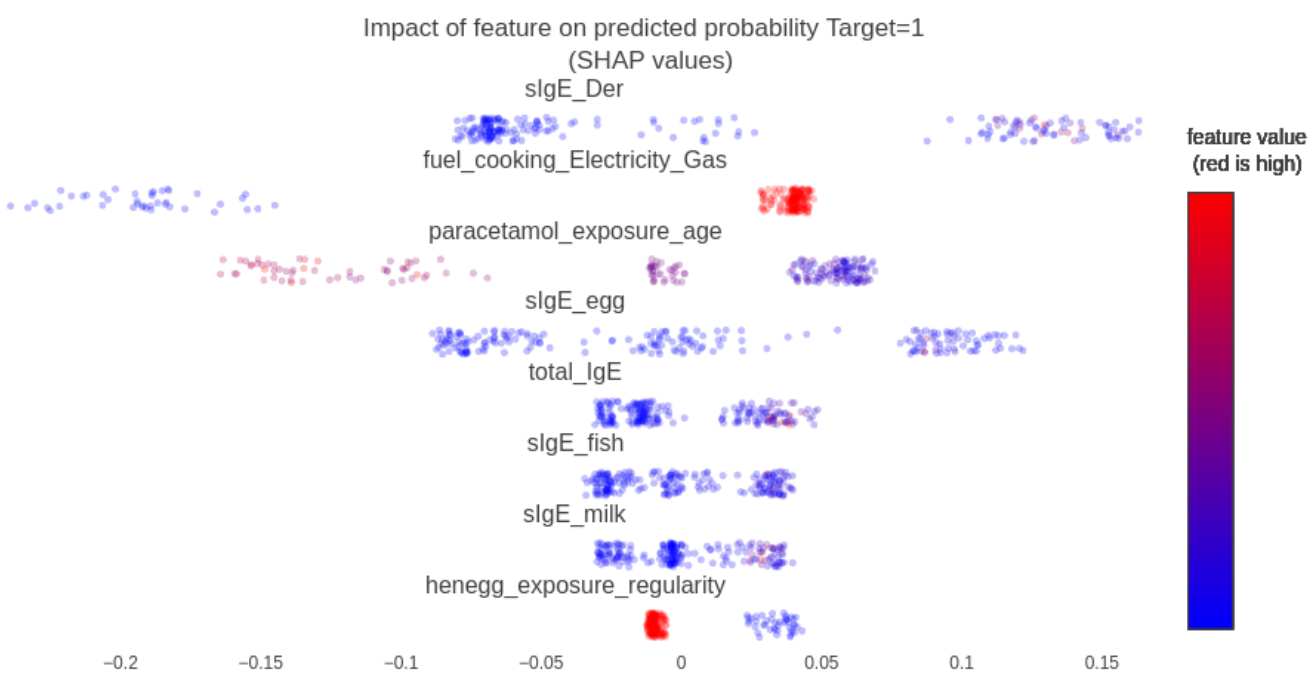

3.1.3 Model: ID14 (MLP)

The feature importance calculated with SHAP values in Model14 (best model overall) looks like this:

In [25]:
```
display(Image(filename="data/output/feature_importance_14_shap.png", width='75%'
```

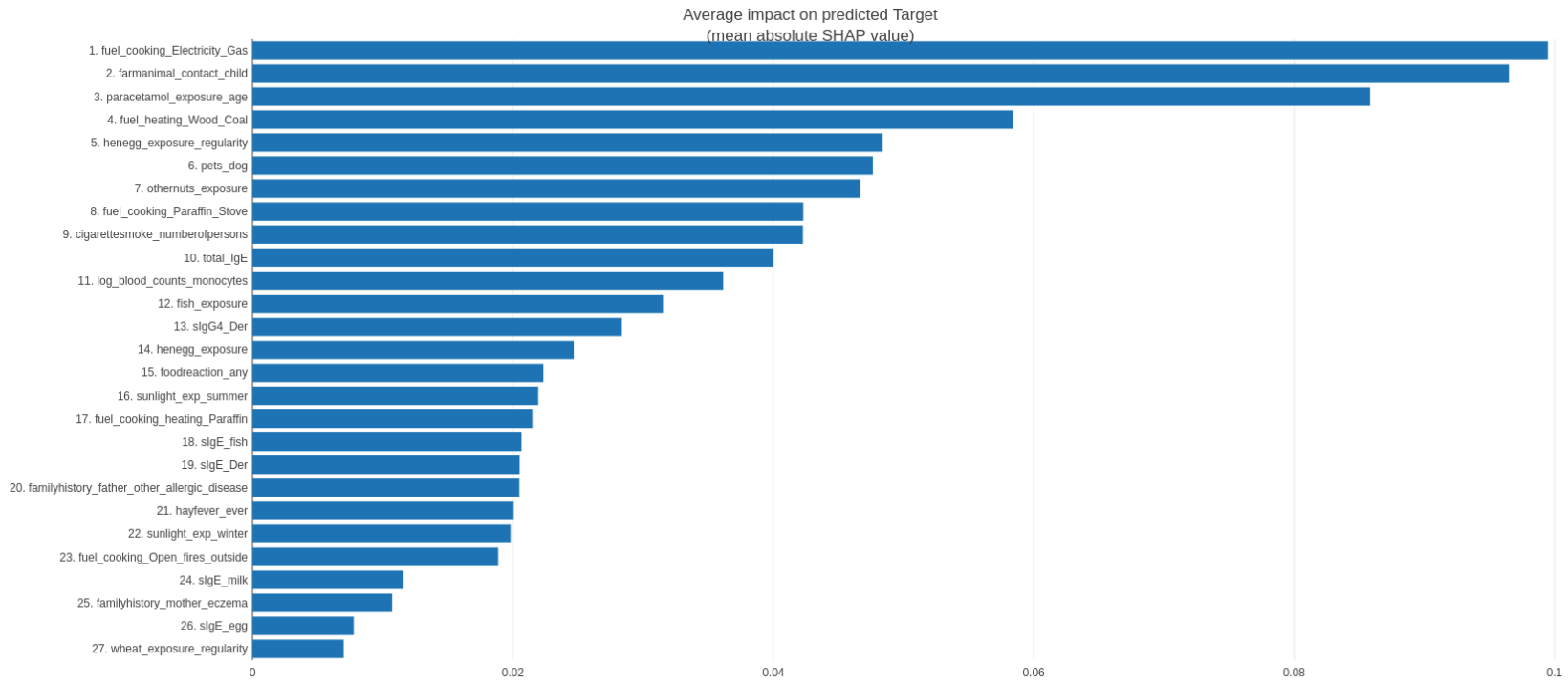

And feature importance using feature permutation:

In [26]:
```
display(Image(filename="data/output/feature_importance_14_permutation.png", widt
```

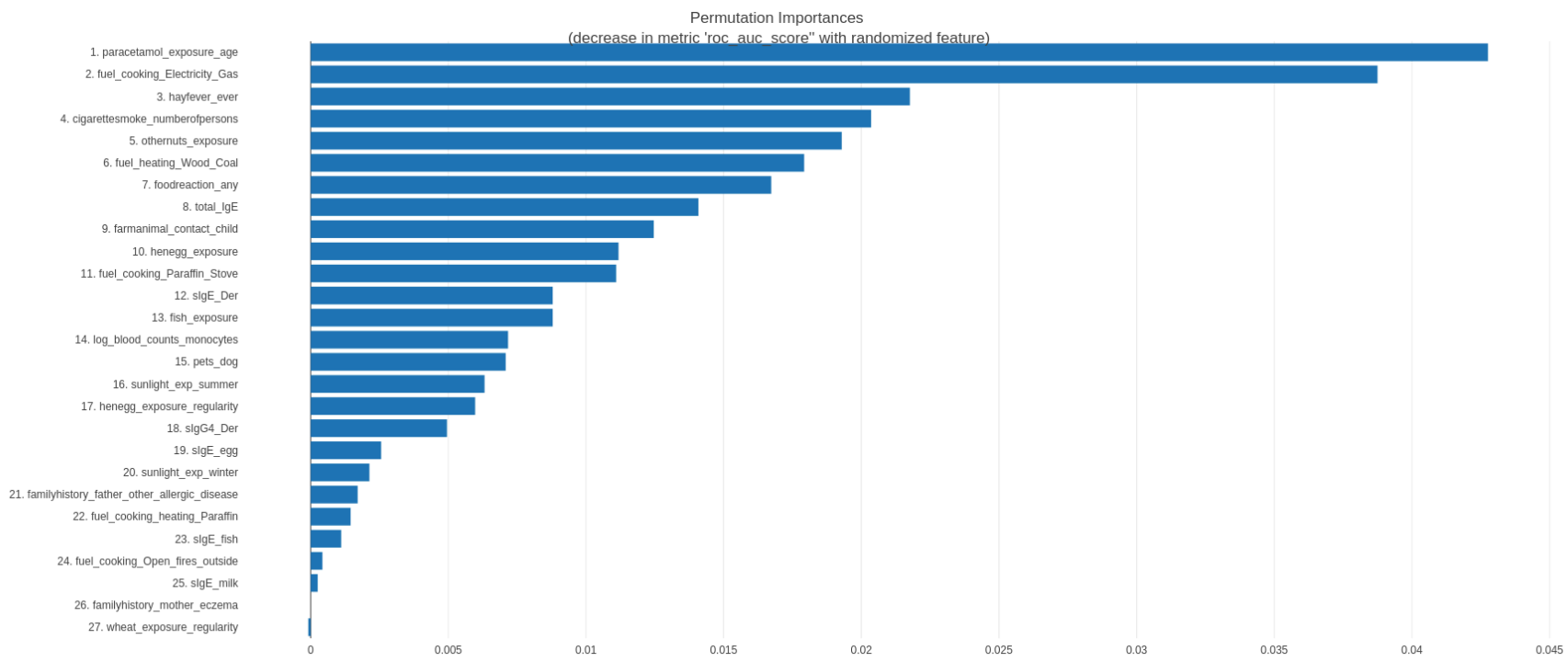

Interestingly feature `sIgE_Der` that seems to be very important for RF and XGBOOST isn't that important for a neuronal network. The feature distributions seems to be a little different in case of MLP.

SHAP detailed view (first 8 features):

In [27]:
```
display(Image(filename="data/output/shap_detailed_summary_14.png", width='60%'))
```

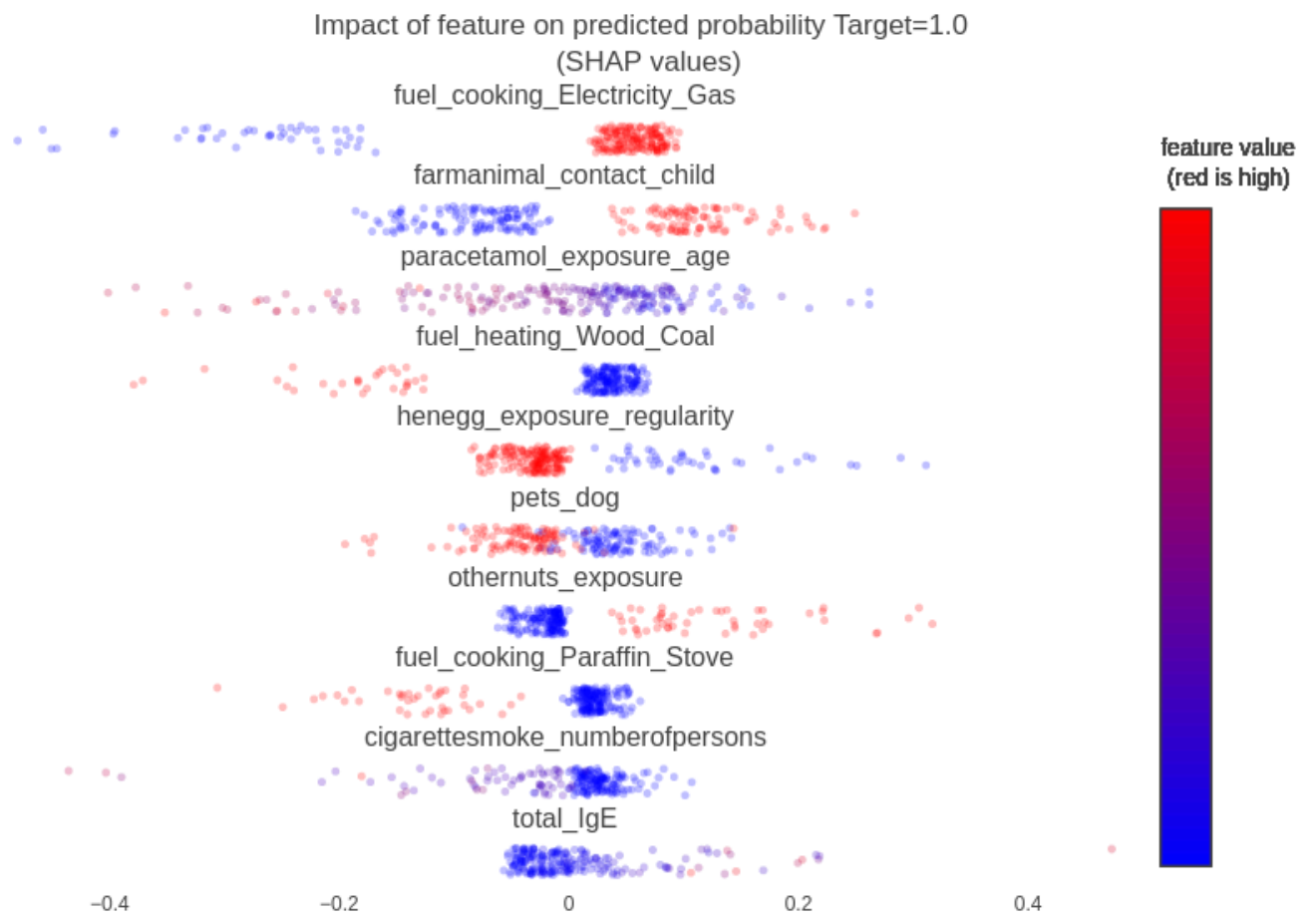

In [ ]:
