# Supplementary material for: Multimodal protective and susceptibility clusters in paediatric atopic dermatitis: A machine learning-based, data-driven observational study
Source: PLoS Med. 2026 Sep 8;23(9):e1004917. doi: 10.1371/journal.pmed.1004917 (PMC13552787; doi:10.1371/journal.pmed.1004917)
Supplement: Supplementary file 7 [file pmed.1004917.s010.pdf]

Reviewer #1:

"Protective and Susceptibility Clusters of Environmental Factors, Gene Expression, Antibody Responses, and Cytokines in Pediatric Atopic Dermatitis: Insights from Multi-Modal Data Integration" employs machine learning (ML) approaches to identify novel clusters of features associated with atopic dermatitis (AD), on a balanced dataset (n=200+; roughly half with AD) of South African AmaXhosa children (12-36 months). Various protective and susceptibility clusters were found using latent component modelling.

While the analysis is comprehensive, detailed and includes multiple modalities, the organization of the manuscript could be improved given the relative complexity of the ML pipeline and analyses - particularly for the methodology.

Response: As detailed below, we have attempted to improve the organisation of the manuscript in response to the various concerns, particularly regarding the methodology. In the new S2 File, we also provide a comprehensive description of the ML methods used to analyse the clinical dataset comprising the combined questionnaire and antibody data. We hope this clarifies our analysis strategy and workflow.

Some other issues might also be considered:

1. From Section 2, the full dataset consisted of four (sub-)datasets: 1) environmental exposures/living conditions (from a questionnaire); 2) clinical data; 3) antibody data; 4) RNA sequencing data. It might first be clarified as to which of these datasets the allergen/cytokine data described in the next subsection was assigned to.

Response: We agree with the reviewer that this was not clearly formulated. In the revised Methods section, we now include the following statement (new text in blue):

[Allergen Sensitization, Allergen Antibody and Cytokine Measurements

To establish the antibody data that form part of the clinical dataset, skin prick tests were performed as previously reported in (8), using Alk-Abello (Alk-Abello, Denmark) reagents for sensitization to cow's milk, …]

We have also changed the table header of S3 Table to:

S3 Table: List of features in the pre-processed clinical dataset corresponding to the combined questionnaire and antibody data.

2. It is then stated that "...The hyperparameter search space also included selection of the ML algorithms". Does this imply that the four classifiers might not have been evaluated the same number of times? If so, how was the stopping criteria decided for the optuna meta-classifier framework?

Response: We thank the reviewer for raising this point. We confirm that the four classifiers have not been evaluated the same number of times; however, this was done by design. Optuna employs a Bayesian optimisation approach (in particular, the Tree-structured Parzen Estimator, TPE) to navigate the hyperparameter search space in a more informed manner. Unlike classical grid or random search,

Optuna adaptively chooses the next set of parameters based on previous results. Parameters (including the choice of algorithm) that yield poor performance are deprioritised and sampled less frequently, while more promising ones receive more trials. As a result, underperforming classifiers are naturally deprioritised, which reduces the computational resources required compared to exhaustive search. This is a well-established property of Bayesian optimisation and is a key motivation for using this strategy over simpler approaches. Including algorithm selection as a categorical hyperparameter is consistent with how Optuna is used in the literature and in its reference documentation.
Regarding the stopping criterion, limits of n_trials = 150 and a timeout of 36'000 s were defined, and the timeout was reached first. After 10 hours, 77 trials were executed, and the process was terminated.
To clarify these points, the detailed ML methods have been included as a new S2 File, and the Methods section of the manuscript now reads as follows (new text in blue):
[In the ML classification, AD vs HC was set as the target variable. All data were included to build the models using cross-validation as the main aim was not to build a single best model, but to extract the features that contributed most to predictive performance. To conduct the hyperparameter search for the models, the Bayesian optimisation-based Optuna framework was used, which employs the Tree-structured Parzen Estimator (TPE) to navigate the hyperparameter search space in an informed manner. Unlike classical random or grid searches, Optuna samples combinations of hyperparameters, including algorithm choice, that yield better performance, while deprioritising less optimal ones. This approach enabled the selection of hyperparameter settings that were most likely to yield a high classification score (16). As the stopping criterion, limits of n_trials = 150 and a timeout of 36,000 s were defined, and the timeout was reached first after 78 trials were executed. …]

3. A five-fold cross-validation is then described on the questionnaire and antibody data, with the best model performance (of 78 models) reported as from Model 52, with a cross-validation mean accuracy of 0.81. However, the results for this classification do not appear to be reported. S2.3.1 for example shows the Random Forest model as having an accuracy of about 0.62.
Response: We thank the reviewer for highlighting a potential source of confusion regarding the reported classification performance results. We have now included the classification performance bar plot for M52, together with details of the full cross-validation procedure, in the Supporting Information (new Figure S2 and new File S2), and adjusted the text in the Methods section as follows (new text in blue):
[The pipeline was then evaluated for every hyperparameter combination in a 5-fold cross-validation (CV) process, using mean accuracy as the classification performance measure, as the distribution between AD and HC in the data is nearly equal. As described in detail in S2 File, all data preprocessing steps were applied separately within each CV fold. After the thorough model building process, 78 models were trained and the best model performance was reached in Model 52 (M52). This best-performing Random Forest model had a cross-validation mean accuracy score of 0.81. Other classification metrics were similarly high (approximately 0.80) for both CV and the test set (S2 Fig.). …]

Regarding the Random Forest model with an accuracy of 0.62, this model was used in the feature selection procedure for the RNA-seq data within the GeneSelectR pipeline and is not related to the clinical data.

4. In general, the ML analysis does not appear to be very systematic. For example, while AD vs. HC was defined as the target variable for ML classification, the first set of classifiers was trained and (cross-)validated using only the questionnaire and antibody data. Feature selection is then done on RNA-Seq data, before DIABLO is applied to the integrated/combined data from all modalities. Given this, it is unclear why all the data (from all modalities) could not have been collated first, before feature selection and then classification/clustering modelling. The Appendix further presents another feature selection step for the combined data (S3.2.3), and variable selection from DIABLO (S3.3.1). However, it is unclear whether these insights from DIABLO were used to develop any further ML classifier.
Response: We thank the reviewer for this comment, and we acknowledge that the presentation of the ML pipeline could be explained more clearly. We also agree that the analysis could be enhanced with more explicit justification for the integration strategy described.
In our analysis, we follow a late data integration framework, which is an established methodology in multi-modal data integration (Picard et al., 2021, DOI: 10.1016/j.csbj.2021.06.030; Sibilio et al., 2025, DOI: 10.1016/j.btre.2025.e00938). In brief, in a late integration approach, modality-specific preprocessing and feature selection are done independently, followed by joint classification modelling. This is a deliberate design choice, as it offers several advantages: it reduces the problem of the curse of dimensionality already introduced by the RNA-Seq dataset, which has a much higher number of features compared to the other datasets. Furthermore, it prevents RNA-Seq from dominating the modelling process by taking up most of the feature space prior to integration.
Regarding the alternative of collating the dataset at an earlier stage before the feature selection procedure, while it is a valid alternative, early integration could introduce a problem of overfitting due to the dominance of one dataset with extremely high dimensionality (RNA-Seq), especially in cohorts with limited sample size. Therefore, a modality-specific feature selection step was necessary to bring the feature spaces to a comparable scale. To address this point, we have added the following text to the Methods section (new text in blue):
[Machine Learning on Integrated Data
To combine different data modalities, we adopted a late data integration strategy (26,27). In this approach, feature selection is performed on each sub-dataset separately to reduce dimensionality and avoid introducing redundant noise into the combined dataset. The sub-datasets were then matched using a complete-case approach, resulting in 132 samples with patient information, antibody data, RNA-Seq data, and quantitative cytokine data (S1 Fig.).]

We also clarified that DIABLO was used here as an exploratory and biological interpretation tool to identify co-varying features across modalities, rather than a step in the ML classification pipeline itself. Since the main goal of our study was explainable ML and biological interpretation, its outputs were

used for informed biological interpretation but were not used to develop a predictive model. We have added the following text to the Discussion to make this distinction explicit:
[Several limitations of this study should be noted. First, the ML approaches used here were not intended to produce a deployable predictive model for clinical use. Rather, they were applied as an explainable AI framework to explore the underlying structure of the data and to generate mechanistic insights into the pathogenesis of AD. …]

5. Given that the DIABLO model can also be used as a classifier, its performance metrics might also be reported.
Response: We thank the reviewer for this comment and for pointing out that DIABLO could be used as a classification tool. While we agree with this statement, we would like to clarify that our study was primarily focused on exploring the data and deriving biological insights; therefore, the DIABLO approach was used as an explainable AI (exAI) tool rather than a predictive model. As noted above, we have clarified this point and highlighted it in the revised text.

Reviewer #2:
Overall comment:This study investigated environmental, immunological, and transcriptomic factors associated with atopic dermatitis (AD) in 217 AmaXhosa children aged 12-36 months living in rural and urban South Africa. Using an integrated systems biology approach, the authors analyzed questionnaire-derived environmental exposures, allergen-specific and total IgE antibodies, plasma cytokines, and PBMC RNA sequencing data. Machine learning methods were applied for feature selection, classification, and multi-omics integration.The analysis identified distinct clusters of features associated with AD status. Rural environmental exposures were linked to specific cytokine patterns and autophagy-related gene expression signatures, whereas AD susceptibility was associated with elevated IgE responses and chemokines such as MCP-4 and TARC. Integration of multi-modal datasets further revealed correlated immune and transcriptomic patterns distinguishing children with and without AD. Overall, the study applies explainable machine learning and multi-omics integration to characterize environmental and immune features associated with early-childhood AD in an underrepresented population. The findings will be potentially impactful.
Response: We thank the reviewer for the positive assessment of our work and are grateful for the provided feedback.

Major comments:
1.Please clarify whether imputation, scaling, and feature selection were performed within each training fold. Was nested cross-validation used during hyperparameter tuning? Were folds stratified by outcome? Without this clarification, the reported mean CV accuracy of 0.81 cannot be reliably interpreted.
Response: We thank the reviewer for the comment and for raising these important methodological points. We confirm that, following standard machine learning practices, all operations were performed

within each training fold to prevent potential data leakage. We have added a more detailed description of the analysis in the Supplementary Material (new S2 File).

Regarding nested cross-validation, hyperparameter tuning was performed using the same cross-validation folds as those used to estimate model performance. We acknowledge that this may introduce a modest optimistic bias in the reported CV accuracy. However, given the size of the hyperparameter search space, we expect this effect to be within an acceptable margin.

Regarding stratification, the dataset has a balanced class distribution (approximately 50/50), which is expected to result in similar performance for stratified and non-stratified splits. Therefore, the effect of stratification is likely negligible.

2.The manuscript reports that 78 models were trained and the best-performing model (M52) selected based on mean CV accuracy. Selecting the best model from multiple tuned candidates using the same validation structure introduces optimistic bias. Furthermore, only accuracy is reported. Even with near-balanced classes, accuracy alone might be insufficient. Please provide the following as much as possible/ appropriate: AUC (ROC), Sensitivity and specificity, Balanced error rate, Confusion matrix, Clarification on whether model selection was nested. If no independent validation cohort is available, the predictive claims should be framed as exploratory.

Response: We thank the reviewer for this comment. We have included the cross-validation and repeated test set split metrics (F1, AUC–ROC, precision, and recall) in the Supporting Information (S2 Fig.). The M52 assessment is also included in the GitHub repository. In addition, we have expanded the limitations section to emphasise that the aim of the study was to provide mechanistic insights rather than to develop a predictive model for practical use. To make this distinction explicit, we have added the following text to the Discussion (new text in blue):

[Several limitations of this study should be noted. First, the ML approaches used here were not intended to produce a deployable predictive model for clinical use. Rather, they were applied as an explainable AI framework to explore the underlying structure of the data and to generate mechanistic insights into the pathogenesis of AD. …]

3.The rural-urban contrast underpins several biological interpretations, including "protective environmental clusters." However, the Methods do not describe adjustment for: Age within the 12-36 month range, Sex, Socioeconomic status, Batch effects (for RNA-Seq and cytokines). Given structural differences between rural and urban environments, unadjusted comparisons may reflect confounding rather than biological protection. Please comment

Response: We thank the reviewer for raising this point and would like to clarify how these factors were handled.

- Age and sex. The cohort was balanced for age (12–36 months) and sex across rural and urban groups at the level of the study design, as described in the first paper (Lunjani *et al.,* 2022). This is further supported empirically: sex did not differ significantly between groups (point-biserial $r = 0.10$, $p = 0.13$), and age showed only a modest difference within the pre-specified enrolment window (rural median 20 months vs urban 24 months; $r = 0.17$, $p = 0.01$) (Tables R1 and R2).

- Socioeconomic status. Parental income and education were collected and are reported in Table R1. Both show the expected association with the rural–urban contrast (r = 0.19, p = 0.005 and r = 0.25, p < 0.001, respectively), reflecting the well-known structural coupling of socioeconomic status with environment in this setting. Notably, median parental education was identical in both groups, indicating substantial overlap rather than stratification. We therefore interpret our findings as reflecting the composite rural–urban environment, of which socioeconomic status is an integral component, rather than a biological effect to be disentangled from socioeconomic status.
- Batch effects. RNA-Seq and cytokine batches were structured along the rural–urban axis by cohort design. We addressed this by including the rural–urban variable in the design formula of our differential analyses, so that this structure is explicitly modelled. This has now been clarified in the Methods.
- Machine learning robustness. Our ML analyses relied primarily on tree-based methods, which are comparatively robust to correlated covariates, and our multi-label classification experiments used balanced class representation by design.

Table R1: Assessment of the statistical differences between rural and urban with regard to the socio-economic variables ‘Parental income’ and ‘Parental education’, as well as enrolment age and sex.

Variable	Rural median [IQR] (n)	Urban median [IQR] (n)	Test	p-value
Parental income	2150 [1300–3615] (n=111)	5500 [3160–11000] (n=93)	Mann–Whitney U	<0.001
Parental education (years)	14 [12–16] (n=112)	14 [14–16] (n=103)		0.001
Enrolment age (months)	20 [15–26] (n=112)	24 [17–30] (n=105)		0.012
Sex	1 [1–2] (n=111)	1 [1–2] (n=105)		0.145

Table R2: Association of covariates with the rural–urban group indicator.

Covariate	Method	Value (r)	p-value
Enrolment age	Point-biserial r	0.174	0.010
Gender		0.103	0.130
Parental income		0.189	0.005
Parental education		0.248	<0.001

4.RNA-Seq analysis using edgeR is appropriate; however, it is unclear whether batch, age, sex, or other covariates were included. Additionally, PBMC transcriptomes are sensitive to cell composition shifts. No mention is made of cell-type deconvolution or adjustment. Without accounting for cellular heterogeneity, differential expression may reflect altered immune cell proportions rather than transcriptional regulation.

Response: We thank the reviewer for this point and would like to address these issues.

- Our differential gene expression analysis included the rural–urban variable together with the diagnosis in the design formula, so that batch-related variation is explicitly modelled. As detailed in our response to comment 3 above, age and sex were balanced at the level of study design (Lunjani *et al.*, 2022), and our empirical checks confirm that sex does not differ between groups (p = 0.13), while age shows only a modest shift within the prespecified 12–36 months window (p = 0.01). In the design formula of edgeR, the four groups were set as comparisons.
- Regarding cell type deconvolution, we deliberately did not apply this approach for two related reasons. First, reliable deconvolution of paediatric PBMC transcriptomes requires a matched, high-quality single-cell reference dataset that captures relevant immune cell states in this specific demographic. In its absence, deconvolution may introduce reference-driven bias rather than remove biological variation. Second, in the biological context of this study, regulatory and compositional components of the signal are not fully separable, as changes in immune cell proportions and changes in their transcriptional programmes tend to be coupled as aspects of the same response, and both are informative for the study questions. We therefore interpret the bulk RNA-Seq signal as reflecting this combined biology.

5.The structure of missing data across modalities has not been sufficiently described. The study includes differing sample sizes for clinical (n=217), antibody (n=152), RNA-Seq (n=149), and cytokine (n=159) datasets, but it is unclear how these discrepancies were handled during integration. The authors state that "In running the ML pipeline, missing data were initially imputed using the miceforest package." However, it is not stated whether DIABLO analyses were restricted to overlapping samples with complete data across all modalities, how partially missing data blocks were treated, or whether a complete-case approach was applied. Clarification is needed to understand the effective sample size for integrated analyses and the potential impact on statistical power and bias.

Response: We thank the reviewer for this helpful comment and agree that data missingness deserves a more explicit description.

- Missing values in the clinical dataset, comprising the combined questionnaire and antibody data, were imputed using the miceforest package, but only for a specified set of features where imputation was biologically and statistically justified. Features for which imputation would have been inappropriate were excluded from imputation and either retained as-is or not used in downstream analyses. The full list of imputed features, together with the imputation parameters and diagnostics, is provided in the new File S2 and in the accompanying GitHub repository (https://github.com/dzhakparov/mlm-sos-all).

- For multi-modal data integration with DIABLO, we used a complete-case approach restricted to the subset of 132 participants with matched data across all modalities (Fig. S1). We have now stated this effective sample size explicitly in the Methods (new text in blue):

[Machine Learning on Integrated Data

To combine different data modalities, we adopted a late data integration strategy (26,27). In this approach, feature selection is performed on each sub-dataset separately to reduce dimensionality and avoid introducing redundant noise into the combined dataset. The sub-datasets were then matched using a complete-case approach, resulting in 132 samples with patient information, antibody data, RNA-Seq data, and quantitative cytokine data (S1 Fig.).]

Minor comments:

1.The introduction section is method-heavy. The central research gap and hypothesis are not sharply articulated. The introduction would benefit from clearer conceptual framing, stronger logical flow, and less emphasis on specific analytical tools.

Response: We have thoroughly reviewed especially the last part of the Introduction to make it less technically heavy while still introducing the different tools. It now reads as follows:

[… The analysis of the individual datasets, and particularly their integration, posed several challenges, as the data are complex, multidimensional, and influenced by a strong environmental factor, with substantial differences in the number of features across the datasets. To address this, we first used machine learning (ML) to identify the most important features in the clinical questionnaire and antibody data. This was followed by the calculation of Shapley additive explanation (SHAP) values to understand the contributions of these features to the results (11). For the RNA-Seq data, we applied the dedicated GeneSelectR workflow (12) to select a subset of genes with high discriminatory power and biological relevance. Finally, by integrating all four datasets using Data integration Analysis for biomarker discovery using latent components (DIABLO) (13), we identified clusters of correlated environmental, cytokine, and RNA-Seq features that could be broadly associated with either being protective or increase susceptibility to AD.]

2.Random seed specification and package versions should be reported for reproducibility.

Response: The random seed specification and the versions of all packages used in the analysis are provided in the GitHub repository associated with this study: github.com/dzhakparov/mlm-sos-all.

3.I suggest that the authors address major concerns. Minor limitations can be discussed in a well-developed limitation sub-section under their discussion section.

Response: The following section on limitations has been added to the Discussion section (new text in blue):

[Several limitations of this study should be noted. First, the ML approaches used here were not intended to produce a deployable predictive model for clinical use. Rather, they were applied as an explainable AI framework to explore the underlying structure of the data and to generate mechanistic insights into the pathogenesis of AD. Second, although our findings are based on a well-characterised

paediatric AD cohort, further validation in an independent cohort would be a valuable next step to confirm the generalisability of the identified disease markers and to support the findings of the current study.]

This study has several notable strengths. The cohort is well characterized, with dermatologist-confirmed moderate-to-severe atopic dermatitis in a clearly defined pediatric age group, enhancing diagnostic reliability. The integration of multiple data domains—including environmental questionnaire data, allergen-specific and total IgE measurements, plasma cytokines, and PBMC transcriptomics—reflects a comprehensive systems-level approach that is well aligned with the complex pathophysiology of AD. The authors demonstrate commendable transparency through public availability of sequencing data and analysis code, supporting reproducibility. In addition, the deliberate exclusion of disease-defining variables such as SCORAD from predictive modeling reduces circularity and strengthens the validity of the classification framework. Importantly, the focus on AmaXhosa children in South Africa addresses a significant gap in multi-omics research in underrepresented African populations. Collectively, these strengths enhance the potential impact of the study, provided that the methodological concerns outlined above are adequately addressed.
Response: We thank the reviewer for the overall favourable assessment of the strengths and potential impact of the manuscript. We believe that the methodological concerns outlined above have been addressed in the revised manuscript and the accompanying Supporting Information, where additional details and clarifications are provided.

Reviewer #3:
This is a follow on study on material collected and reported in a previous study (PMID PMID: 34086351), where the authors described an association between AD and elevated levels of specific IgE to food and environmental antigens (i.e. atopy), eosinophilia, and a suite of serum cytokines. In the current study, the authors have analysed the dataset, including many of the same readouts, but supplemented this with bulk RNAseq data from PBMCs using a complex ML strategy. Despite these efforts to identify correlations, overall, the associations appear to be in line with their previous publication. In its current form, based on the analysis presented, the results amount to a small incremental advance on their previous publication. At a minimum, the authors should clarify what has been done previously with the same dataset, and any new insights from the current manuscript that were not reported previously. It is possible that the major advance is methodological (see comment 6).
Response: We thank the reviewer for this comment and the opportunity to clarify the novelty of the present study.
We agree that this work builds on previously published datasets (Lunjani et al., 2022, 2024). While the previous studies were either conducted on subsets of the data or primarily focused on describing immunological readouts in relation to environmental differences between rural and urban settings, which represent the dominant variable in this cohort, the aim of the present study was to address a different and more challenging question, namely the comparison between AD and healthy individuals in the presence of this strong environmental effect. This required the analysis of a complex,

multidimensional dataset comprising multiple data types with substantially different numbers of variables, a scenario that is highly relevant for many clinical studies.

To tackle this, we implemented an integrative analysis strategy combining multiple advanced methods, with a particular emphasis on making the results interpretable. Rather than analysing individual data layers in isolation, we performed a joint analysis of questionnaire, antibody, cytokine, and RNA-Seq data. This enabled us to identify features with strong discriminatory power, as well as clusters of correlated variables across data modalities that are associated with either protection or increased susceptibility to AD.

We therefore see the main advance of this study as both conceptual and methodological. Conceptually, it provides an integrated view of AD in this cohort that goes beyond previously reported single-modality associations. Methodologically, it establishes a robust and explainable workflow for the analysis of complex, heterogeneous clinical datasets, which we believe will be of broader relevance.

Major comments

1. The description of the cohort lacks sufficient detail to determine potential for bias. Although the inclusion criteria is stated (a dermatologist's diagnosis of AD), how was the cohort assembled? In other words, from what population of individuals meeting the criterion of a dermatologist's diagnosis was the study sample drawn? How were cases identified, and what proportion of cases meeting inclusion criteria were included, and what were the reasons for exclusion?

Response: We thank the reviewer for these comments and the opportunity to clarify these important points. This study was designed as a matched case control study conducted at one time-point in patients with moderate to severe AD. All participants were from the ethno-linguistic AmaXhosa population and aged 12-36 months. Urban children with moderate to severe atopic dermatitis were enrolled at the Paediatric Dermatology Clinic, Red Cross Children's Hospital, Cape Town. Healthy non-allergic, non-food sensitized (on SPT screening) participants were enrolled from randomly selected crèches in the Cape Town metropole. Similarly, children from a rural setting with AD were enrolled at the dermatology clinic at the Nelson Mandela Academic Hospital, Umthatha. Their non-allergic healthy control counterparts were randomly selected at study sites based at village clinics in the Mqanduli district of Umthatha. All participants were seen at one time point for questionnaire, SPT, clinical assessment and blood sample collection.

This information has been added to the Methods section of the manuscript (new text in blue):

[As detailed in (8,15) this study employed a single-visit, cross-sectional case-control design involving AmaXhosa children in South Africa who were between 12-36 months old, lived in either a rural or urban environment, and were either healthy or had a dermatologist's diagnosis of moderate to severe AD, fulfilling the UK Working Party criteria for AD. Exclusion criteria included other considerable health conditions, immune-mediated diseases other than allergic comorbidities and positive stool and serum IgE tests for helminths. Urban children with moderate to severe AD were enrolled at the Paediatric Dermatology Clinic, Red Cross Children's Hospital, Cape Town. Healthy non-allergic, non-food sensitized (on skin prick test (SPT) screening) participants were enrolled from randomly selected

crèches in the Cape Town metropole. Similarly, children from a rural setting with AD were enrolled at the dermatology clinic at the Nelson Mandela Academic Hospital, Umthatha. Their non-allergic healthy control counterparts were randomly selected at study sites based at village clinics in the Mqanduli district of Umthatha. All participants were seen at one time point for questionnaire, SPT, clinical assessment, and blood sample collection. A standardised questionnaire was used to obtain data on the environmental exposures and living conditions of the children. In total, standard questionnaire data on environmental exposures and living conditions of the children were obtained for we obtained clinical data on patient characteristics of 217 AmaXhosa children ..]

2. Did the healthy control population contain atopic individuals. If not, how was the atopic status of cases and controls determined?

Response: As already detailed in the response above and added to the Methods section, none of the controls were atopic. This was confirmed by clinical history, negative allergen SPT responses, and low levels of serum allergen-specific IgE. All cases had a doctor-diagnosed moderate to severe AD.

3. Did any of the cases or controls come from the same family, or the same household?

Response: Cases and controls came from different families and households.

4. Sunlight exposure was reported in the analysis. This is a notoriously difficult variate to ascertain accurately. How was it determined in the current study?

Response: Sunlight exposure was calculated by asking the child's parent how many hours per day were typically spent outdoors in winter time and summer time.

5. The analysis focusses on RNAseq dataset from bulk PBMCs. One of the confounders of bulk RNAseq is differences in abundance of cell subsets in the PBMC compartment, and although the study is billed as a multi-omics analysis, no information is provided about variation in the cellular composition of the PBMCs in patients.

Response: As detailed above in our response to Reviewer 2, we deliberately did not apply cell type deconvolution for two related reasons. First, reliable deconvolution of paediatric PBMC transcriptomes requires a matched, high-quality single-cell reference dataset that captures the relevant immune cell states in this specific demographic. In its absence, deconvolution may introduce reference-driven bias rather than remove biological variation. Second, in the biological context of this study, regulatory and compositional components of the signal are not fully separable, as changes in immune cell proportions and changes in their transcriptional programmes tend to be coupled as aspects of the same response, and both are informative for the study questions. We therefore interpret the bulk RNA-Seq signal as reflecting this combined biology.

6. The most striking finding from the RNAseq data was the effect of location. This prompted a machine learning approach to identify signals that might be associated with AD. Although some signals were identified from the models developed, none of these were verified with biological studies. The majority of the analysis is a description of the ML strategy, which is probably outside of the interests of a general medical audience and would probably be better suited to a bio-informatics journal. At a minimum, the authors should improve the labelling of their figures to assist the reader in interpreting how the results in one figure relate to those presented elsewhere. In particular, features should be labelled appropriately, rather than with codes.

Response: We have rewritten the Introduction to reduce technical density and place greater emphasis on the clinical and biological motivation of the study, thereby improving its accessibility to a general medical audience. However, particularly in relation to the figure legends, we note that a certain level of technical detail remains necessary for the correct interpretation of the results.

7. It is possible that the model arrived at based on the ML strategy is informative, but this would need to be confirmed in an independent cohort.

Response: We acknowledge this limitation. However, the primary focus of this study was on advanced analytical approaches to identify informative features and to enhance the interpretability of the results. To clarify this point, we have added the following statement to the Discussion section addressing this limitation:

[Several limitations of this study should be noted. First, the ML approaches used here were not intended to produce a deployable predictive model for clinical use. Rather, they were applied as an explainable AI framework to explore the underlying structure of the data and to generate mechanistic insights into the pathogenesis of AD. Second, although our findings are based on a well-characterised paediatric AD cohort, further validation in an independent cohort would be a valuable next step to confirm the generalisability of the identified disease markers and to support the findings of the current study.]
