# Supplementary material for: Multimodal protective and susceptibility clusters in paediatric atopic dermatitis: A machine learning-based, data-driven observational study
Source: PLoS Med. 2026 Sep 8;23(9):e1004917. doi: 10.1371/journal.pmed.1004917 (PMC13552787; doi:10.1371/journal.pmed.1004917)
Supplement: Peer review history

# Editor report: Decision Letter 0

**DOI:** 10.1371/journal.pmed.1004917.r001

**Authors:** Katarzyna Starowicz

12 Jan 2026

Dear Dr Baerenfaller,

Thank you for submitting your manuscript entitled "Protective and Susceptibility Clusters of Environmental Factors, Gene Expression, Antibody Responses, and Cytokines in Pediatric Atopic Dermatitis: Insights from Multi-Modal Data Integration" for consideration by PLOS Medicine.

Your manuscript has now been evaluated by the PLOS Medicine editorial staff, and I am writing to let you know that we would like to send your submission out for external peer review.

However, before we can send your manuscript to reviewers, we need you to complete your submission by providing the metadata that is required for full assessment. To this end, please login to Editorial Manager where you will find the paper in the 'Submissions Needing Revisions' folder on your homepage. Please click 'Revise Submission' from the Action Links and complete all additional questions in the submission questionnaire.

For clinical studies, please upload a copy of your trial study protocol as a supporting information file. The study protocol should be the version submitted for approval to the institutional review board or ethics committee, should include any amendments to the study protocol, as well as the date of their approval by the institutional review or ethics committee. Please also detail any deviations from the study protocol in the Methods section of your manuscript. The editors will consider the protocol and study conduct prior to a final decision for external review.

Please re-submit your manuscript within two working days, i.e. by Jan 14 2026 11:59PM.

Login to Editorial Manager here: https://www.editorialmanager.com/pmedicine

Once your full submission is complete, your paper will undergo a series of checks in preparation for peer review. Once your manuscript has passed all checks it will be sent out for review.

Feel free to email us at plosmedicine@plos.org if you have any queries relating to your submission.

Kind regards,

Katarzyna Starowicz, PhD

Associate Editor

PLOS Medicine

kstarowicz@plos.org

# Peer review: Decision Letter 1

**DOI:** 10.1371/journal.pmed.1004917.r002

**Authors:** Katarzyna Starowicz

1 Apr 2026

Dear Dr Baerenfaller,

Many thanks for submitting your manuscript "Protective and Susceptibility Clusters of Environmental Factors, Gene Expression, Antibody Responses, and Cytokines in Pediatric Atopic Dermatitis: Insights from Multi-Modal Data Integration" (PMEDICINE-D-26-00121R1) to PLOS Medicine. Please accept my sincere apologies for delay regarding decision on your manuscript. The paper has been reviewed by subject experts and a statistician; their comments are included below and can also be accessed here: [LINK]

After discussing the paper with the editorial team and an academic editor with relevant expertise, I'm pleased to invite you to revise the paper in response to the reviewers' comments. As you will see, the reviewers finds your study interesting and with possible clinical impact however, they requested further clarifications and raised some concerns around methodology and validation. We would be happy to reconsidered revised manuscript if reviewers' concerns will be addressed in full, but we cannot provide any guarantees at this stage regarding publication.

In addition to these revisions, you may need to complete some formatting changes, which you will receive in a follow up email. A member of our team will be in touch with a set of requests shortly. If you do not receive a separate email within a few days, please assume that checks have been completed, and no additional changes are required.

When you upload your revision, please include a point-by-point response that addresses all of the reviewer and editorial points, indicating the changes made in the manuscript and either an excerpt of the revised text or the location (eg: page and line number) where each change can be found. Please also be sure to check the general editorial comments at the end of this letter and include these in your point-by-point response. When you resubmit your paper, please include a clean version of the paper as the main article file and a version with changes tracked as a marked-up manuscript. It may also be helpful to check the guidelines for revised papers at http://journals.plos.org/plosmedicine/s/revising-your-manuscript for any that apply to your paper.

We ask that you submit your revision by Apr 22 2026 11:59PM. However, if this deadline is not feasible, please contact me by email, and we can discuss a suitable alternative.

Don't hesitate to contact me directly with any questions (kstarowicz@plos.org).

Best regards,

Katarzyna

Katarzyna Starowicz, PhD

Associate Editor

PLOS Medicine

kstarowicz@plos.org

-----------------------------------------------------------

Comments from the reviewers:

Reviewer #1: "Protective and Susceptibility Clusters of Environmental Factors, Gene Expression, Antibody Responses, and Cytokines in Pediatric Atopic Dermatitis: Insights from Multi-Modal Data Integration" employs machine learning (ML) approaches to identify novel clusters of features associated with atopic dermatitis (AD), on a balanced dataset (n=200+; roughly half with AD) of South African AmaXhosa children (12-36 months). Various protective and susceptibility clusters were found using latent component modelling.

While the analysis is comprehensive, detailed and includes multiple modalities, the organization of the manuscript could be improved given the relative complexity of the ML pipeline and analyses - particularly for the methodology. Some other issues might also be considered:

1. From Section 2, the full dataset consisted of four (sub-)datasets: 1) environmental exposures/living conditions (from a questionnaire); 2) clinical data; 3) antibody data; 4) RNA sequencing data. It might first be clarified as to which of these datasets the allergen/cytokine data described in the next subsection was assigned to.

2. It is then stated that "...The hyperparameter search space also included selection of the ML algorithms". Does this imply that the four classifiers might not have been evaluated the same number of times? If so, how was the stopping criteria decided for the optuna meta-classifier framework?

3. A five-fold cross-validation is then described on the questionnaire and antibody data, with the best model performance (of 78 models) reported as from Model 52, with a cross-validation mean accuracy of 0.81. However, the results for this classification do not appear to be reported. S2.3.1 for example shows the Random Forest model as having an accuracy of about 0.62.

4. In general, the ML analysis does not appear to be very systematic. For example, while AD vs. HC was defined as the target variable for ML classification, the first set of classifiers was trained and (cross-)validated using only the questionnaire and antibody data. Feature selection is then done on RNA-Seq data, before DIABLO is applied to the integrated/combined data from all modalities.

Given this, it is unclear why all the data (from all modalities) could not have been collated first, before feature selection and then classification/clustering modelling. The Appendix further presents another feature selection step for the combined data (S3.2.3), and variable selection from DIABLO (S3.3.1). However, it is unclear whether these insights from DIABLO were used to develop any further ML classifier.

5. Given that the DIABLO model can also be used as a classifier, its performance metrics might also be reported.

Reviewer #2: Overall comment:

This study investigated environmental, immunological, and transcriptomic factors associated with atopic dermatitis (AD) in 217 AmaXhosa children aged 12-36 months living in rural and urban South Africa. Using an integrated systems biology approach, the authors analyzed questionnaire-derived environmental exposures, allergen-specific and total IgE antibodies, plasma cytokines, and PBMC RNA sequencing data. Machine learning methods were applied for feature selection, classification, and multi-omics integration.

The analysis identified distinct clusters of features associated with AD status. Rural environmental exposures were linked to specific cytokine patterns and autophagy-related gene expression signatures, whereas AD susceptibility was associated with elevated IgE responses and chemokines such as MCP-4 and TARC. Integration of multi-modal datasets further revealed correlated immune and transcriptomic patterns distinguishing children with and without AD.

Overall, the study applies explainable machine learning and multi-omics integration to characterize environmental and immune features associated with early-childhood AD in an underrepresented population. The findings will be potentially impactful.

Major comments:

1.Please clarify whether imputation, scaling, and feature selection were performed within each training fold. Was nested cross-validation used during hyperparameter tuning? Were folds stratified by outcome? Without this clarification, the reported mean CV accuracy of 0.81 cannot be reliably interpreted.

2.The manuscript reports that 78 models were trained and the best-performing model (M52) selected based on mean CV accuracy. Selecting the best model from multiple tuned candidates using the same validation structure introduces optimistic bias. Furthermore, only accuracy is reported. Even with near-balanced classes, accuracy alone might be insufficient.

Please provide the following as much as possible/ appropriate: AUC (ROC), Sensitivity and specificity, Balanced error rate, Confusion matrix, Clarification on whether model selection was nested. If no independent validation cohort is available, the predictive claims should be framed as exploratory.

3.The rural-urban contrast underpins several biological interpretations, including "protective environmental clusters." However, the Methods do not describe adjustment for: Age within the 12-36 month range, Sex, Socioeconomic status, Batch effects (for RNA-Seq and cytokines). Given structural differences between rural and urban environments, unadjusted comparisons may reflect confounding rather than biological protection. Please comment

4.RNA-Seq analysis using edgeR is appropriate; however, it is unclear whether batch, age, sex, or other covariates were included. Additionally, PBMC transcriptomes are sensitive to cell composition shifts. No mention is made of cell-type deconvolution or adjustment. Without accounting for cellular heterogeneity, differential expression may reflect altered immune cell proportions rather than transcriptional regulation.

5.The structure of missing data across modalities has not been sufficiently described. The study includes differing sample sizes for clinical (n=217), antibody (n=152), RNA-Seq (n=149), and cytokine (n=159) datasets, but it is unclear how these discrepancies were handled during integration. The authors state that "In running the ML pipeline, missing data were initially imputed using the miceforest package." However, it is not stated whether DIABLO analyses were restricted to overlapping samples with complete data across all modalities, how partially missing data blocks were treated, or whether a complete-case approach was applied. Clarification is needed to understand the effective sample size for integrated analyses and the potential impact on statistical power and bias.

Minor comments:

1.The introduction section is method-heavy. The central research gap and hypothesis are not sharply articulated. The introduction would benefit from clearer conceptual framing, stronger logical flow, and less emphasis on specific analytical tools.

2.Random seed specification and package versions should be reported for reproducibility.

3.I suggest that the authors address major concerns. Minor limitations can be discussed in a well-developed limitation sub-section under their discussion section.

This study has several notable strengths. The cohort is well characterized, with dermatologist-confirmed moderate-to-severe atopic dermatitis in a clearly defined pediatric age group, enhancing diagnostic reliability. The integration of multiple data domains—including environmental questionnaire data, allergen-specific and total IgE measurements, plasma cytokines, and PBMC transcriptomics—reflects a comprehensive systems-level approach that is well aligned with the complex pathophysiology of AD. The authors demonstrate commendable transparency through public availability of sequencing data and analysis code, supporting reproducibility. In addition, the deliberate exclusion of disease-defining variables such as SCORAD from predictive modeling reduces circularity and strengthens the validity of the classification framework. Importantly, the focus on AmaXhosa children in South Africa addresses a significant gap in multi-omics research in underrepresented African populations. Collectively, these strengths enhance the potential impact of the study, provided that the methodological concerns outlined above are adequately addressed.

Reviewer #3: This is a follow on study on material collected and reported in a previous study (PMID PMID: 34086351), where the authors described an association between AD and elevated levels of specific IgE to food and environmental antigens (i.e. atopy), eosinophilia, and a suite of serum cytokines. In the current study, the authors have analysed the dataset, including many of the same readouts, but supplemented this with bulk RNAseq data from PBMCs using a complex ML strategy. Despite these efforts to identify correlations, overall, the associations appear to be in line with their previous publication. In its current form, based on the analysis presented, the results amount to a small incremental advance on their previous publication. At a minimum, the authors should clarify what has been done previously with the same dataset, and any new insights from the current manuscript that were not reported previously. It is possible that the major advance is methodological (see comment 6).

Major comments

1. The description of the cohort lacks sufficient detail to determine potential for bias. Although the inclusion criteria is stated (a dermatologist's diagnosis of AD), how was the cohort assembled? In other words, from what population of individuals meeting the criterion of a dermatologist's diagnosis was the study sample drawn? How were cases identified, and what proportion of cases meeting inclusion criteria were included, and what were the reasons for exclusion?

2. Did the healthy control population contain atopic individuals. If not, how was the atopic status of cases and controls determined?

3. Did any of the cases or controls come from the same family, or the same household?

4. Sunlight exposure was reported in the analysis. This is a notoriously difficult variate to ascertain accurately. How was it determined in the current study?

5. The analysis focusses on RNAseq dataset from bulk PBMCs. One of the confounders of bulk RNAseq is differences in abundance of cell subsets in the PBMC compartment, and although the study is billed as a multi-omics analysis, no information is provided about variation in the cellular composition of the PBMCs in patients.

6. The most striking finding from the RNAseq data was the effect of location. This prompted a machine learning approach to identify signals that might be associated with AD. Although some signals were identified from the models developed, none of these were verified with biological studies. The majority of the analysis is a description of the ML strategy, which is probably outside of the interests of a general medical audience and would probably be better suited to a bio-informatics journal. At a minimum, the authors should improve the labelling of their figures to assist the reader in interpreting how the results in one figure relate to those presented elsewhere. In particular, features should be labelled appropriately, rather than with codes.

7. It is possible that the model arrived at based on the ML strategy is informative, but this would need to be confirmed in an independent cohort.

Any attachments provided with reviews can be seen via the following link: [LINK]

--------------------------------------------------------- ---

General editorial requests:

(Note: not all will apply to your paper, but please check each item carefully)

*We ask every co-author listed on the manuscript to fill in a contributing author statement, making sure to declare all competing interests. If any of the co-authors have not filled in the statement, we will remind them to do so when the paper is revised. If all statements are not completed in a timely fashion this could hold up the re-review process. If new competing interests are declared later in the revision process, this may also hold up the submission. Should there be a problem getting one of your co-authors to fill in a statement we will be in contact. Please do not add or remove authors without first discussing this with the handling editor. You can see our competing interests policy here: http://journals.plos.org/plosmedicine/s/competing-interests.

*Please upload any figures associated with your paper as individual TIF or EPS files with 300dpi resolution at resubmission; please read our figure guidelines for more information on our requirements: http://journals.plos.org/plosmedicine/s/figures. While revising your submission, we strongly recommend that you use PLOS's NAAS tool (https://ngplosjournals.pagemajik.ai/artanalysis) to test your figure files. NAAS can convert your figure files to the TIFF file type and meet basic requirements (such as print size, resolution), or provide you with a report on issues that do not meet our requirements and that NAAS cannot fix.

After uploading your figures to PLOS's NAAS tool - https://ngplosjournals.pagemajik.ai/artanalysis, NAAS will process the files provided and display the results in the "Uploaded Files" section of the page as the processing is complete.

If the uploaded figures meet our requirements (or NAAS is able to fix the files to meet our requirements), the figure will be marked as "fixed" above. If NAAS is unable to fix the files, a red "failed" label will appear above.

When NAAS has confirmed that the figure files meet our requirements, please download the file via the download option, and include these NAAS processed figure files when submitting your revised manuscript.

*Please ensure that the paper adheres to the PLOS Data Availability Policy (see http://journals.plos.org/plosmedicine/s/data-availability), which requires that all data underlying the study's findings be provided in a repository or as Supporting Information. For data residing with a third party, authors are required to provide instructions with contact information (web or email address) for obtaining the data. Please note that a study author cannot be the contact person for the data. PLOS journals do not allow statements supported by "data not shown" or "unpublished results." For such statements, authors must provide supporting data or cite public sources that include it.

*We expect all researchers with submissions to PLOS in which author-generated code underpins the findings in the manuscript to make all author-generated code available without restrictions upon publication of the work. In cases where code is central to the manuscript, we may require the code to be made available as a condition of publication. Authors are responsible for ensuring that the code is reusable and well documented. Please make any custom code available, either as part of your data deposition or as a supplementary file. Please add a sentence to your data availability statement regarding any code used in the study, e.g. "The code used in the analysis is available from Github [URL] and archived in Zenodo [DOI link]" Please review our guidelines at https://journals.plos.org/plosmedicine/s/materials-software-and-code-sharing and ensure that your code is shared in a way that follows best practice and facilitates reproducibility and reuse. Because Github depositions can be readily changed or deleted, we encourage you to make a permanent DOI'd copy (e.g. in Zenodo) and provide the URL.

*Please ensure that the study is reported according to the [XXXX] guideline and include the completed [XXXX] checklist as Supporting Information. When completing the checklist, please use section and paragraph numbers, rather than page numbers. Please add the following statement, or similar, to the Methods: "This study is reported as per [XXXX] guideline (S1 Checklist)."

FORMATTING - GENERAL

*Abstract: Please structure your abstract using the PLOS Medicine headings (Background, Methods and Findings, Conclusions). Please combine the Methods and Findings sections into one section.

*At this stage, we ask that you include a short, non-technical Author Summary of your research to make findings accessible to a wide audience that includes both scientists and non-scientists. The Author Summary should immediately follow the Abstract in your revised manuscript. This text is subject to editorial change and should be distinct from the scientific abstract. Ideally each sub-heading should contain 2-3 single sentence, concise bullet points containing the most salient points from your study. In the final bullet point of 'What Do These Findings Mean?', please include the main limitations of the study in non-technical language. Please see our author guidelines for more information: https://journals.plos.org/plosmedicine/s/revising-your-manuscript#loc-author-summary.

*Please express the main results with 95% CIs as well as p values. When reporting p values please report as p<0.001 and where higher as the exact p value p=0.002, for example. Throughout, suggest reporting statistical information as follows to improve clarity for the reader "22% (95% CI [13%,28%]; p</=)". Please be sure to define all numerical values at first use.

*Please include page numbers and line numbers in the manuscript file. Use continuous line numbers (do not restart the numbering on each page).

*Please cite the reference numbers in square brackets. Citations should precede punctuation.

FIGURES AND TABLES

*Please provide titles and legends for all figures and tables (including those in Supporting Information files).

*Please define all abbreviations used in each figure/table (including those in Supporting Information files).

*Please consider avoiding the use of red and green in order to make your figure more accessible to those with color blindness.

SUPPLEMENTARY MATERIAL

*Please note that supplementary material will be posted as supplied by the authors. Therefore, please amend it according to the relevant comments outlined here.

*Please cite your Supporting Information as outlined here: https://journals.plos.org/plosmedicine/s/supporting-information

REFERENCES

*PLOS uses the numbered citation (citation-sequence) method and first six authors, et al.

*Please ensure that journal name abbreviations match those found in the National Center for Biotechnology Information (NCBI) databases (http://www.ncbi.nlm.nih.gov/nlmcatalog/journals), and are appropriately formatted and capitalised.

*Where website addresses are cited, please include the complete URL and specify the date of access (e.g. [accessed: 12/06/2023]).

*Please also see https://journals.plos.org/plosmedicine/s/submission-guidelines#loc-references for further details on reference formatting.

STUDY TYPE-SPECIFIC REQUESTS - please note not all will apply:

OBSERVATIONAL STUDIES

*Abstract: Please include the study design, population and setting, number of participants, years during which the study took place (enrollment and follow up), length of follow up, and main outcome measures.

*Please ensure that the study is reported according to the STROBE (or appropriate STOBE extension) guideline (available from: https://www.equator-network.org/reporting-guidelines/strobe) and include the completed STROBE (or STROBE extension) checklist as Supporting Information. Please add the following statement, or similar, to the Methods: "This study is reported as per the Strengthening the Reporting of Observational Studies in Epidemiology (STROBE) guideline (S1 Checklist)." When completing the checklist, please use section and paragraph numbers, rather than page numbers.

*[FOR POPULATION HEALTH/REGISTRY STUDIES] Please ensure that the study is reported according to the RECORD guideline (available from https://www.record-statement.org) and include the completed checklist as Supporting Information. Please add the following statement, or similar, to the Methods: "This study is reported as per the Reporting of Studies Conducted using Observational Routinely-Collected Data (RECORD) guideline (S1 Checklist)." When completing the checklist, please use section and paragraph numbers, rather than page numbers.

*[FOR POPULATION HEALTH ESTIMATES] Please ensure that the study is reported according to the GATHER statement (available from https://www.equator-network.org/reporting-guidelines/gather-statement) and include the completed checklist as Supporting Information. Please add the following statement, or similar, to the Methods: "This study is reported as per the Guidelines for Accurate and Transparent Health Estimates Reporting (GATHER) statement (S1 Checklist)." When completing the checklist, please use section and paragraph numbers, rather than page numbers.

*[FOR MEDIATION ANALYSES] We recommend that the study is reported according to the AGReMA statement (https://agrema-statement.org/#:~:text=AGReMA%20is%20an%20evidence%2D%20and,randomised%20trials%20and%20observational%20studies) and include the completed checklist as Supporting Information. Please add the following statement, or similar, to the Methods: "This study is reported as per the Guideline for Reporting Mediation Analyses (AGReMA) statement (S1 Checklist)." When completing the checklist, please use section and paragraph numbers, rather than page numbers.

*For all observational studies, in the manuscript text, please indicate: (1) the specific hypotheses you intended to test, (2) the analytical methods by which you planned to test them, (3) the analyses you actually performed, and (4) when reported analyses differ from those that were planned, transparent explanations for differences that affect the reliability of the study's results. If a reported analysis was performed based on an interesting but unanticipated pattern in the data, please be clear that the analysis was data driven.

*Please state in the Methods section whether the study had a prospective protocol or analysis plan. If a prospective analysis plan (from your funding proposal, IRB or other ethics committee submission, study protocol, or other planning document written before analyzing the data) was used in designing the study, please include the relevant document(s) with your revised manuscript as a Supporting Information file to be published alongside your study and cite it in the Methods section. A legend for this file should be included at the end of your manuscript. If no such document exists, please make sure that the Methods section transparently describes when analyses were planned, and when/why any data-driven changes to analyses took place. Changes in the analysis, including those made in response to peer review comments, should be identified as such in the Methods section of the paper, with rationale.

MODELLING STUDIES

The following list is derived from Geoffrey P Garnett, Simon Cousens, Timothy B Hallett, Richard Steketee, Neff Walker. Mathematical models in the evaluation of health programmes. (2011) Lancet DOI:10.1016/S0140-6736(10)61505-X:

*If pertinent, please provide a diagram that shows the model structure, including how the natural history of the disease is represented, the process and determinants of disease acquisition, and how the putative intervention could affect the system.

*Please provide a complete list of model parameters, including clear and precise descriptions of the meaning of each parameter, together with the values or ranges for each, with justification or the primary source cited and important caveats about the use of these values noted.

*Please provide a clear statement about how the model was fitted to the data, including goodness-of-fit measure, the numerical algorithm used, which parameter varied, constraints imposed on parameter values, and starting conditions.

*For uncertainty analyses, please state the sources of uncertainties quantified and not quantified [can include parameter, data, and model structure].

*Please provide sensitivity analyses to identify which parameter values are most important in the model. Uncertainty estimates seek to derive a range of credible results on the basis of an exploration of the range of reasonable parameter values. The choice of method should be presented and justified.

*Please discuss the scientific rationale for the choice of model structure and identify points where this choice could influence conclusions drawn. Please also describe the strength of the scientific basis underlying the key model assumptions.

*For studies that develop a prediction model or evaluate its performance, please ensure that the study is reported according to the TRIPOD statement (https://www.equator-network.org/reporting-guidelines/tripod-statement) and include the completed checklist as Supporting Information. Please add the following statement, or similar, to the Methods: "This study is reported as per the Transparent Reporting of a Multivariable Prediction Model for Individual Prognosis Or Diagnosis (TRIPOD) statement (S1 Checklist)." For studies using machine learning, please use the TRIPOD-AI checklist. When completing the checklist, please use section and paragraph numbers, rather than page numbers.

# Author comment: Author response to Decision Letter 2

**DOI:** 10.1371/journal.pmed.1004917.r003

27 Apr 2026

Attachment Submitted filename: Response_to_Reviewers.pdf

# Peer review: Decision Letter 2

**DOI:** 10.1371/journal.pmed.1004917.r004

**Authors:** Katarzyna Starowicz

23 Jun 2026

Dear Dr. Baerenfaller,

Thank you very much for re-submitting your manuscript "Protective and Susceptibility Clusters of Environmental Factors, Gene Expression, Antibody Responses, and Cytokines in Paediatric Atopic Dermatitis: Insights from Multi-Modal Data Integration" (PMEDICINE-D-26-00121R2) for review by PLOS Medicine.

On behalf of my colleague, Dr. Starowicz, who has discussed the paper with our colleagues and the academic editor, and it was also seen again by two of the original reviewers, I am pleased to say that provided the remaining editorial and production issues are dealt with we are planning to accept the paper for publication in the journal.

The remaining issues that need to be addressed are listed at the end of this email. Any accompanying reviewer attachments can be seen via the link below. Please take these into account before resubmitting your manuscript:

[LINK]

***Please note while forming your response, if your article is accepted, you may have the opportunity to make the peer review history publicly available. The record will include editor decision letters (with reviews) and your responses to reviewer comments. If eligible, we will contact you to opt in or out.***

In revising the manuscript for further consideration here, please ensure you address the specific points made by each reviewer and the editors. In your rebuttal letter you should indicate your response to the reviewers' and editors' comments and the changes you have made in the manuscript. Please submit a clean version of the paper as the main article file. A version with changes marked must also be uploaded as a marked up manuscript file.

Please also check the guidelines for revised papers at http://journals.plos.org/plosmedicine/s/revising-your-manuscript for any that apply to your paper. If you haven't already, we ask that you provide a short, non-technical Author Summary of your research to make findings accessible to a wide audience that includes both scientists and non-scientists. The Author Summary should immediately follow the Abstract in your revised manuscript. This text is subject to editorial change and should be distinct from the scientific abstract.

In addition to these revisions, you may need to complete some formatting changes, which you will receive in a follow up email. A member of our team will be in touch with a set of requests shortly. If you do not receive a separate email within a few days, please assume that checks have been completed, and no additional changes are required.

We expect to receive your revised manuscript within 1 week. Please email us (plosmedicine@plos.org) if you have any questions or concerns.

We ask every co-author listed on the manuscript to fill in a contributing author statement. If any of the co-authors have not filled in the statement, we will remind them to do so when the paper is revised. If all statements are not completed in a timely fashion this could hold up the re-review process. Should there be a problem getting one of your co-authors to fill in a statement we will be in contact. YOU MUST NOT ADD OR REMOVE AUTHORS UNLESS YOU HAVE ALERTED THE EDITOR HANDLING THE MANUSCRIPT TO THE CHANGE AND THEY SPECIFICALLY HAVE AGREED TO IT.

Please ensure that the paper adheres to the PLOS Data Availability Policy (see http://journals.plos.org/plosmedicine/s/data-availability), which requires that all data underlying the study's findings be provided in a repository or as Supporting Information. For data residing with a third party, authors are required to provide instructions with contact information for obtaining the data. PLOS journals do not allow statements supported by "data not shown" or "unpublished results." For such statements, authors must provide supporting data or cite public sources that include it.

To enhance the reproducibility of your results, we recommend that you deposit your laboratory protocols in protocols.io, where a protocol can be assigned its own identifier (DOI) such that it can be cited independently in the future. Additionally, PLOS ONE offers an option to publish peer-reviewed clinical study protocols. Read more information on sharing protocols at https://plos.org/protocols?utm_medium=editorial-email&utm_source=authorletters&utm_campaign=protocols

Please review your reference list to ensure that it is complete and correct. If you have cited papers that have been retracted, please include the rationale for doing so in the manuscript text, or remove these references and replace them with relevant current references. Any changes to the reference list should be mentioned in the rebuttal letter that accompanies your revised manuscript.

Please note, when your manuscript is accepted, an uncorrected proof of your manuscript will be published online ahead of the final version, unless you've already opted out via the online submission form. If, for any reason, you do not want an earlier version of your manuscript published online or are unsure if you have already indicated as such, please let the journal staff know immediately at plosmedicine@plos.org.

If you have any questions in the meantime, please contact me or the journal staff on plosmedicine@plos.org.

We look forward to receiving the revised manuscript by Jun 30 2026 11:59PM.

Sincerely,

Alison Farrell, PhD

Senior Editor

PLOS Medicine

plosmedicine.org

------------------------------------------------------------

Requests from Editors:

Specific requests:

**The Abstract and Author Summary provide no explanation for why this study was performed in AmaXhosa children. Only in the Introduction does the reader understand that studies in predominantly white populations may not translate to African children. Please revise and reframe.

In the Background of the Abstract, please explain in which population you are addressing this question and why.

Please also clarify what gap this research fills, given that there are known AD susceptibility features. Explain further why you embarked on this study.

The first sentence of the Methods and findings does not explain why you used existing datasets, or how you used them. Please revise.

Please use the active voice throughout the Abstract. For example, the second sentence of the Methods and findings requires revision.

The Methods and findings should explain the primary outcomes, not the key findings. Please revise.

The Abstract lacks discrete data. Please revise.

The use of 'various' in the first sentence of the Conclusions section ot the Abstract is vague. Please revise.

Please provide specific conclusions related to your data and temper/avoid any extrapolations.

Author summary:

* First bullet point should explain WHY this study was done. The second bullet point can be deleted. The third bullet point should explain why this type of analysis might generate new findings not previously identified.

* Please do not repeat statements in the first 2 subsections of the Author Summary.

* The Author Summary should be understandable by a lay audience. Please reframe reference to SHAP analysis and GeneSelectR.

In the "What do these findings mean", please revise conclusions for a general audience. The first bullet point can be removed.

Please explain how this study provided new insight into AD and what these insights are.

Introduction:

Please clarify use of AmaXhose and Xhosa.

The Introduction suggests that the data on which this study is based is only derived from the authors. Please confirm to editor if this is correct. Please revise statement if not.

Please break the Introduction into 2 paragraphs.

Results:

Please provide an explanation of the study cohort and number of participants, before discussing specific results.

Please clarify results so that subject matter experts (dermatologists, immunologists, other clinicians) who are not expert in AI studies will find the results accessible.

Discussion:

Please provide 2 introductory sentences to the first paragraph of the Discussion before restating results. Please abide by the above requests regarding accessibility.

The Discussion should re-explain the novel, biological findings, explain their potential clinical relevance, specify the study limitations, and discuss next steps to validate or implement these findings.

GENERAL EDITORIAL REQUESTS

* Please confirm that your title complies with PLOS Medicine's style. Your title must be nondeclarative and not a question. It should begin with main concept if possible. "Effect of" should be used only if causality can be inferred, i.e., for an RCT. Please place the study design ("A randomized controlled trial," "A retrospective study," "A modelling study," etc.) in the subtitle (ie, after a colon). Your title needs to be revised.

* Please ensure that the Introduction ends with a clear description of the study question or hypothesis.

* Please ensure that all abbreviations are defined at first use throughout the text.

* Please confirm that all numbers presented in the abstract are present and identical to numbers presented in the main manuscript text.

GENERAL

* Please include a study protocol.

* If your study involved a prespecified statistical analysis plan, please include as supplementary information.

* Please disclose any use of AI tools and technologies to the study or article content at the end of the Methods. Please specify the name(s) and version(s) of any tool(s) used and a description of their use, including how the AI-assisted or generated content was evaluated by authors, and a clear statement of which aspects of the study, article contents, data, or supporting files were affected/generated by AI tool usage. Please also include a statement specifying who retains responsibility for the final manuscript. See our guidelines for more information: https://journals.plos.org/plosmedicine/s/ethical-publishing-practice#loc-artificial-intelligence-tools-and-technologies.

* Statistical reporting: Please revise throughout the manuscript, including tables and figures.

- Please report statistical information as follows to improve clarity for the reader "22% (95% CI [13,28]; p</=)".

- Please separate upper and lower bounds with commas instead of hyphens as the latter can be confused with reporting of negative values.

- Please repeat statistical definitions (HR, CI etc.) for each set of parentheses.

* In the last sentence of the Abstract Methods and Findings section, please describe the main limitation(s) of the study's methodology.

* In the abstract, please include the important dependent variables that are adjusted for in the analyses.

* In the author summary, in the final bullet point of 'What Do These Findings Mean?', please include the main limitations of the study in non-technical language.

FUNDING STATEMENT

* The funding statement should include: specific grant numbers, initials of authors who received each award, URLs to sponsors’ websites. Also, please state whether any sponsors or funders (other than the named authors) played any role in study design, data collection and analysis, the decision to publish, or preparation of the manuscript. If they had no role in the research, include this sentence: “The funders had no role in study design, data collection and analysis, decision to publish, or preparation of the manuscript.”

COMPETING INTERESTS STATEMENT

* All authors must declare their relevant competing interests per the PLOS policy, which can be seen here: https://journals.plos.org/plosmedicine/s/competing-interests For authors with ties to industry, please indicate whether any of the interests has a financial stake in the results of the current study.

*Please note that funding information should go to the relevant section and include URLs and should not be included in the Acknowledgements.

DATA AVAILABILITY

* The Data Availability Statement (DAS) requires revision. For each data source used in your study:

a) If the data are freely or publicly available, note this and state the location of the data: within the paper, in Supporting Information files, or in a public repository (include the DOI or accession number).

b) If the data are owned by a third party but freely available upon request, please note this and state the owner of the data set and contact information for data requests (web or email address). Note that a study author cannot be the contact person for the data.

c) If the data are not freely available, please describe briefly the ethical, legal, or contractual restriction that prevents you from sharing it. Please also include an appropriate contact (web or email address) for inquiries (again, this cannot be a study author).

* Please include the statement on code availability in the data availability statement. Please see guidance here: https://journals.plos.org/plosmedicine/s/code-sharing-guidance#loc-data-availability-statement-examples

ETHICS AND CONSENT

*Please clarify how consent was obtained and in what language. For parents or caregivers with illiteracy, please state how consent was obtained in those instances.

FIGURES

* Please provide titles and legends for all figures and tables (including those in Supporting Information files). Please define all acronyms used in each figure or table in its corresponding legend.

* Please avoid the use of red and green in order to make your figure more accessible. In particular we ask you to revise the heat map colours in Figure 2. Please also revise Figure 1 and any supplementary figures with similar red-green combinations that may not be accessible to persons with colour blindness.

INCLUSIVITY IN GLOBAL RESEARCH

PLOS has a 'Inclusivity in Global Research' policy which aims to promote collaboration and inclusivity in global health research. Please complete PLOS’ questionnaire on inclusivity in global research and submit it with your revised paper. The policy and questionnaire can be found at https://journals.plos.org/plosone/s/best-practices-in-research-reporting.

ACKNOWLEDGMENTS

* Please include an acknowledgment of study participants.

* Please include in the Acknowledgments recognition of individuals who played a role in data collection or participant care or involvement

AUTHOR CONTRIBUTION

* Please provide an author contribution statement.

Comments from Reviewers:

Reviewer #1: We thank the authors for addressing our previous comments.

Reviewer #2: The authors have sufficiently addressed my comments and concerns. Thanks.

Any attachments provided with reviews can be seen via the following link:

[LINK]

# Editor report: Decision Letter 3

**DOI:** 10.1371/journal.pmed.1004917.r005

**Authors:** Katarzyna Starowicz

24 Jul 2026

Dear Dr Baerenfaller,

On behalf of my colleagues and the Academic Editor, Zulfiqar Bhutta, I am pleased to inform you that we have agreed to publish your manuscript "Multimodal protective and susceptibility clusters in paediatric atopic dermatitis: insights from a machine learning-based, data-driven observational study " (PMEDICINE-D-26-00121R3) in PLOS Medicine.

Before your manuscript can be formally accepted you will need to complete some formatting changes, which you will receive in a follow up email. Please be aware that it may take several days for you to receive this email; during this time no action is required by you. Once you have received these formatting requests, please note that your manuscript will not be scheduled for publication until you have made the required changes.

Please also note the following issues that require your attention:

Please revise the title to: "Multimodal protective and susceptibility clusters in paediatric atopic dermatitis: A machine learning-based, data-driven observational study"

Please add the funders' URL(s) to the funding statement.

In the legends of Figures 1 and 2 (and supplementary figure legends, if relevant) please correct the reference to the colour gradient (where you changed, for example, the red-green colour gradient in Fig. 2, the legend still refers to a red-green gradient). Please also check the Fig. 3 legend.

Please provide a copy of the questionnaire as part of the supplementary information as this is behind a firewall of a previous publication.

Note that co-authors CA and ML have not completed the COI questionnaire. Please follow-up.

In the meantime, please log into Editorial Manager at http://www.editorialmanager.com/pmedicine/, click the "Update My Information" link at the top of the page, and update your user information to ensure an efficient production process.

PRESS

We frequently collaborate with press offices. If your institution or institutions have a press office, please notify them about your upcoming paper at this point, to enable them to help maximise its impact. If the press office is planning to promote your findings, we would be grateful if they could coordinate with medicinepress@plos.org. If you have not yet opted out of the early version process, we ask that you notify us immediately of any press plans so that we may do so on your behalf.

We also ask that you take this opportunity to read our Embargo Policy regarding the discussion, promotion and media coverage of work that is yet to be published by PLOS. As your manuscript is not yet published, it is bound by the conditions of our Embargo Policy. Please be aware that this policy is in place both to ensure that any press coverage of your article is fully substantiated and to provide a direct link between such coverage and the published work. For full details of our Embargo Policy, please visit http://www.plos.org/about/media-inquiries/embargo-policy/.

To enhance the reproducibility of your results, we recommend that you deposit your laboratory protocols in protocols.io, where a protocol can be assigned its own identifier (DOI) such that it can be cited independently in the future. Additionally, PLOS ONE offers an option to publish peer-reviewed clinical study protocols. Read more information on sharing protocols at https://plos.org/protocols?utm_medium=editorial-email&utm_source=authorletters&utm_campaign=protocols

Thank you again for submitting to PLOS Medicine. We look forward to publishing your paper.

Sincerely,

Katarzyna Starowicz, PhD

Associate Editor

PLOS Medicine
